# Supplementary material for: In-Host Flat-like Quasispecies: Characterization Methods and Clinical Implications
Source: Microorganisms. 2024 May 17;12(5):1011. doi: 10.3390/microorganisms12051011 (PMC11124460; doi:10.3390/microorganisms12051011)
Supplement: Supplementary file 1 [file microorganisms-12-01011-s001.zip › Supplementary-File S1/FlatQuasispecies_Supplementary - File S1.pdf]

In-host flat-like quasispecies, methods and clinical implications

Supplementary material

Josep Gregori[1], Sergi Colomer-Castell[1,2,3], Marta Ibañez Lligoña [1,2,4], Damir Garcia-Cehic[1,2], Carolina Campos[1,2,3], Maria Buti[1,2,4], Mar Riveiro-Barciela[1,2,4], Cristina Andrés[5,6], Maria Piñana[5,6], Alejandra Gonzalez-Sánchez[5,6], Francisco Rodríguez-Frías[2,7], Maria Francesca Cortese[2,6,7], David Tabernero[1,2,6], Tomás Pumarola[3,5,6,7], Juan Ignacio Esteban[1,2,4], Andrés Antón[5,6], and Josep Quer[1,2,3,4]

[1] Liver Diseases-Viral Hepatitis, Liver Unit, Vall d'Hebron Institut de Recerca (VHIR), Vall d'Hebron Hospital Universitari, Vall d'Hebron Barcelona Hospital Campus, Passeig Vall d'Hebron 119-129, 08035 Barcelona, Spain

[2] Centro de Investigación Biomédica en Red de Enfermedades Hepáticas y Digestivas (CIBERehd), Instituto de Salud Carlos III, Av. Monforte de Lemos, 3-5, 28029 Madrid, Spain

[3] Biochemistry and Molecular Biology Department, UAB, Bellaterra, Spain

[4] Medicine Department, Universitat Autònoma de Barcelona (UAB), Campus de la UAB, Plaça Cívica, 08193 Bellaterra, Spain

[5] Centro de Investigación Biomédica en Red de Enfermedades Infecciosas (CIBERINFEC), Instituto de Salud Carlos III, Av. Monforte de Lemos, 3-5, 28029 Madrid, Spain

[6] Microbiology Department, Vall d'Hebron Institut de Recerca (VHIR), Vall d'Hebron Hospital Universitari, Vall d'Hebron Barcelona Hospital Campus, Passeig Vall d'Hebron 119-129, 08035 Barcelona, Spain

[7] Biochemistry Department, Vall d'Hebron Institut de Recerca (VHIR), Vall d'Hebron Hospital Universitari, Vall d'Hebron Barcelona Hospital Campus, Passeig Vall d'Hebron 119-129, 08035 Barcelona, Spain

Corresponding authors:

Josep Gregori (josep.gregori@gmail.com) and Josep Quer (josep.quer@vhir.org)

1 Diversities, evenness and sampling size

Higher evenness values are observed for the samples S18 and S19, than for the corresponding replicates at approximately four-fold coverage, S16 and S17 (Supplementary Figures S5 and S6). On the other hand, in Supplementary Figure S3 we see lower values for ${}^0D$ in S18 and S19 than in S16 and S17, but approximately the same values for ${}^\infty D$. These results suggest that lower coverages will result in higher evenness values, and the contrary, that higher coverages will result in smoothed evenness values. To confirm this hypothesis, samples S16 and S17 were taken as reference quasispecies populations and were submitted to repeated rarefaction (subsampling without replacement) at coverage fractions 0.5, 0.25, 0.1, and 0.05. The subsampling was repeated 500 times for each sample and fraction, and the Hill numbers of orders 0, 1, 2, 3, and infinity were computed each time. The averages of the 500 values for each sample, fraction, and Hill number are shown in Supplementary Figure S10 and Supplementary Table S12. The corresponding evenness values are shown in Supplementary Figure S11 and Supplementary Table S13. The two samples in both strands show the same results, a significant drop in ${}^0D$ as we reduce the fraction of sampled reads, drop which is reduced as q increases, and which is practically null at ${}^\infty D$. The same behavior as observed in Supplementary Figure 3 with the pairs S16-S18 and S17-S19. The rationale behind these results is that the sample size has stronger effect on the number of observed haplotypes than in the observed frequency of the master haplotype (see Supplementary Table S12). This is a direct consequence of the different sensitivity of the Hill numbers to sampling effort; at higher coverages the number of observed haplotypes is also higher, but the master sequence, that is the haplotype with the highest frequency, is observed at approximately the same frequency in both cases. Since it has highest frequency, it has the lowest sampling error. These results confirm the above stated hypothesis that given two different sample sizes, the resulting evenness index will be higher for the smaller sample.

The sample size effect on QFF, HNP and RLEP is normalized by rarefaction to 194,000 reads, and the results shown in Supplementary Figures S12, S13 and S14, and Supplementary Tables S14, S15 and S16. Note that S18 and S19 were excluded of the rarefaction because of their low coverage. Finally, Supplementary Figure S15 shows the impact of rarefaction on evenness values for each sample to the reference size of 194,000 reads. This is a scatter-plot of the relative difference (rarefied-raw)/rarefied versus the original sample size, for each sample; ${}^1E$, ${}^2E$, ${}^\infty E$ values are represented. Beyond of a confirmation of what observed in subsampling S16 and S17 at different fractions of the original size, this plot shows a linear behaviour to rarefaction for ${}^1E$, ${}^2E$, ${}^\infty E$, except for the ${}^1E$ values of flat-like quasispecies (S10 and above).

2 Supplementary tables

Ampl.Nm	Genebank	Region	Primer.FW	Primer.RV	5' pos	3' pos	ALen
CoV-HKU1	MH940245.1	Spike	ATGCAGATGTAGGTATGCCTACCACT	ATTCTACGTTCCCAATTAAGAGGT	23495	23900	354
CoV-NL63	ON554022.1	Spike	CTGCAAATTTTCTTGATGATAATGT	TCAGACCAAGTAACATACAAAGCA	21794	22220	376
CoV-OC43	PP187318.1	Spike	GCAGGTTTAATCCTTCTACTTGGAAT	CAGACCAACCCAAAAATGCTTGT	24973	25359	336
EV-A71	OR800939.1	VP1	ATGCGYTTTGAYGCAGAGTTCAC	CTGACGTGCTTCATCCTCAT	2844	3213	325
HCV-1B	AF054249	E2	ACTGGTTCGGCTGTACATGGATGA	TGCACGTCCACGATGTTCTGATG	1985	2441	408
HMPV-B	MZ504966.1	Glycop.	ATAGACATGTTCAAAGCAAAGATGA	TGGCTTTTTCTCTGTGGTTGT	6206	6634	381
RSV-A	PP151405.1	Glycop.	TGCTGTAACAGAATTGCAGTTGCT	TTGATATGCTGCAGCTTTGCTT	5924	6306	335
RSV-B	PP135061.1	Glycop.	CATGTTAACAAACAGTGAGTTACTATCAT	AGGCTGACTTCACTTGGTAATGT	6415	6807	339
SARS-CoV-2	MN908947.3	Spike	CAGTGTTATAACACCAGGAACAAAT	ATTTGTGGGTATGGCAATAGAGTTA	23347	23713	315
HCV-G1g	AF166600.1	NS5B	CCACATCAACTCCGTGTGG	CCGACGTACARTCTCTCRGTGAG	7952	8389	394
HCV-G4a	Y11604.1	NS5B	CCACATCARCTCCGTGTGG	CCCACRTAGAGTCTYTCTGTGAG	7952	8389	394
HEV-ORF2	LC770331.1	Core	GTCGTCTCAGCCAATGGCGAGCC	CASARAANGTCTTNGARTACTGCT	6373	6784	363

Table S1: Specific primers and genome positions for each virus. ALen: amplicon length after trimming primers.

ID	Virus	Strand	Reads	Master	Rd1	Rd5
S01	CoV-HKU1	FW	276,201	82.531	1.630	2.130
S01	CoV-HKU1	RV	286,514	82.826	1.638	2.101
S02	CoV-NL63	FW	211,999	75.754	3.145	4.030
S02	CoV-NL63	RV	207,402	73.846	3.647	4.587
S03	CoV-OC43	FW	368,214	70.946	3.971	5.038
S03	CoV-OC43	RV	356,914	70.393	4.143	5.181
S04	EV-A71	FW	344,096	63.891	5.285	7.585
S04	EV-A71	RV	338,345	63.577	5.478	7.812
S05	HCV-1B	FW	293,124	31.413	8.968	12.672
S05	HCV-1B	RV	271,492	30.749	9.353	13.311
S06	HMPV-B	FW	363,464	66.945	4.644	6.361
S06	HMPV-B	RV	356,692	68.835	4.135	5.649
S07	RSV-A	FW	272,451	79.638	2.093	2.730
S07	RSV-A	RV	280,640	79.846	2.047	2.665
S08	RSV-B	FW	347,637	81.978	1.728	2.116
S08	RSV-B	RV	340,815	82.235	1.621	1.998
S09	SARS-CoV-2	FW	263,457	82.432	1.490	1.987
S09	SARS-CoV-2	RV	272,136	82.228	1.544	2.029
S10	gHEV	FW	304,313	8.163	37.749	51.980
S10	gHEV	RV	260,770	7.921	39.661	53.431
S11	gHEV	FW	293,327	8.817	40.674	53.821
S11	gHEV	RV	244,153	8.695	42.434	55.286
S12	gHEV	FW	242,066	5.182	29.919	42.547
S12	gHEV	RV	194,130	5.118	32.255	45.029
S13	gHEV	FW	265,554	0.415	78.417	90.883
S13	gHEV	RV	206,668	0.391	81.062	92.081
S14	gHEV	FW	248,738	2.094	48.906	63.858
S14	gHEV	RV	194,750	1.931	52.105	66.400
S15	gHEV	FW	275,257	1.149	70.070	83.153
S15	gHEV	RV	223,492	1.100	72.495	84.654
S16	gHEV	FW	278,336	0.190	81.824	92.865
S16	gHEV	RV	227,619	0.194	83.477	93.237
S17	gHEV	FW	304,874	0.293	73.428	86.347
S17	gHEV	RV	239,837	0.289	75.126	87.060
S18	gHEV	FW	75,673	0.214	89.954	96.136
S18	gHEV	RV	59,128	0.203	91.009	96.470
S19	gHEV	FW	73,326	0.274	82.694	90.750
S19	gHEV	RV	58,260	0.283	84.301	91.794

Table S2: Total reads per amplicon and strand, frequency of the master haplotype, fraction of reads for singletons, and fraction of reads for haplotypes supported by up to 5 reads. Frequency and fractions expressed as %.

ID	Virus	Reads	Singl	UnqsSingl	AllUnqs
S01	CoV-HKU1	562715	1.634	1.533	1.668
S02	CoV-NL63	419401	3.393	3.106	3.458
S03	CoV-OC43	725128	4.055	3.516	4.107
S04	EV-A71	682441	5.381	4.391	5.409
S05	HCV-1B	564616	9.153	7.984	9.788
S06	HMPV-B	720156	4.392	3.809	4.581
S07	RSV-A	553091	2.069	1.883	2.136
S08	RSV-B	688452	1.675	1.563	1.688
S09	SARS-CoV-2	535593	1.518	1.409	1.536
S10	gHEV	565083	38.631	35.237	41.983
S11	gHEV	537480	41.473	38.364	45.004
S12	gHEV	436196	30.958	28.191	35.540
S13	gHEV	472222	79.575	76.801	83.887
S14	gHEV	443488	50.311	46.566	53.297
S15	gHEV	498749	71.157	67.797	74.099
S16	gHEV	505955	82.568	79.844	85.953
S17	gHEV	544711	74.175	70.937	77.806
S18	gHEV	134801	90.417	88.229	90.790
S19	gHEV	131586	83.406	80.585	84.277

Table S3: Total reads per amplicon, and fractions for singletons, singletons unique to one strand, and reads of haplotypes unique to one strand. Fractions expressed as percentages.

ID	Strand	Master	Emerging	0.1–1%	< 0.1%
CoV-HKU1.S01	FW	82.531	0.000	1.594	15.875
CoV-HKU1.S01	RV	82.826	0.000	1.726	15.448
CoV-NL63.S02	FW	75.754	0.000	4.717	19.529
CoV-NL63.S02	RV	73.846	0.000	5.366	20.788
CoV-OC43.S03	FW	70.946	0.000	6.041	23.013
CoV-OC43.S03	RV	70.393	0.000	6.276	23.331
EV-A71.S04	FW	63.891	0.000	13.629	22.479
EV-A71.S04	RV	63.577	0.000	14.155	22.268
HCV-1B.S05	FW	31.413	22.364	10.541	35.682
HCV-1B.S05	RV	30.749	21.892	11.571	35.787
HMPV-B.S06	FW	66.945	0.000	7.520	25.535
HMPV-B.S06	RV	68.835	0.000	6.489	24.676
RSV-A.S07	FW	79.638	0.000	3.103	17.259
RSV-A.S07	RV	79.846	0.000	2.990	17.164
RSV-B.S08	FW	81.978	0.000	0.927	17.095
RSV-B.S08	RV	82.235	0.000	0.969	16.796
SARS-CoV-2.S09	FW	82.432	0.000	2.200	15.368
SARS-CoV-2.S09	RV	82.228	0.000	2.125	15.647
gHEV.S10	FW	8.163	1.303	12.462	78.071
gHEV.S10	RV	7.921	1.364	12.019	78.696
gHEV.S11	FW	8.817	2.639	9.900	78.644
gHEV.S11	RV	8.695	2.773	9.415	79.116
gHEV.S12	FW	5.182	12.111	15.102	67.606
gHEV.S12	RV	5.118	12.102	15.063	67.718
gHEV.S13	FW	0.415	0.000	0.900	98.685
gHEV.S13	RV	0.391	0.000	0.786	98.822
gHEV.S14	FW	2.094	1.056	10.944	85.907
gHEV.S14	RV	1.931	0.000	12.383	85.686
gHEV.S15	FW	1.149	0.000	1.545	97.306
gHEV.S15	RV	1.100	0.000	1.519	97.381
gHEV.S16	FW	0.190	0.000	0.626	99.184
gHEV.S16	RV	0.194	0.000	0.640	99.166
gHEV.S17	FW	0.293	0.000	2.715	96.992
gHEV.S17	RV	0.289	0.000	2.384	97.327
gHEV.S18	FW	0.214	0.000	0.620	99.166
gHEV.S18	RV	0.203	0.000	0.641	99.156
gHEV.S19	FW	0.274	0.000	3.018	96.708
gHEV.S19	RV	0.283	0.000	2.403	97.314

Table S4: Quasispecies fitness fractions for each amplicon and strand, raw values. Master frequency, emerging as the fraction of reads for haplotypes above 1% and below the master, fraction of reads for haplotypes at frequencies between 0.1 and 1%, and fraction of reads for haplotypes below 0.1%. Frequency and fractions expressed as percentages.

ID	Strand	^0D	^1D	^2D	^3D	$^\infty D$
CoV-HKU1.S01	FW	5799	5.25	1.47	1.33	1.21
CoV-HKU1.S01	RV	5976	5.14	1.46	1.33	1.21
CoV-NL63.S02	FW	8205	9.61	1.74	1.52	1.32
CoV-NL63.S02	RV	9173	11.48	1.83	1.58	1.35
CoV-OC43.S03	FW	17293	15.02	1.99	1.67	1.41
CoV-OC43.S03	RV	17309	15.70	2.02	1.69	1.42
EV-A71.S04	FW	22453	27.33	2.45	1.96	1.57
EV-A71.S04	RV	22797	28.37	2.47	1.97	1.57
HCV-1B.S05	FW	32262	130.91	7.01	5.00	3.18
HCV-1B.S05	RV	31232	141.63	7.32	5.16	3.25
HMPV-B.S06	FW	20466	20.95	2.23	1.83	1.49
HMPV-B.S06	RV	17913	17.29	2.11	1.75	1.45
RSV-A.S07	FW	7134	6.59	1.58	1.41	1.26
RSV-A.S07	RV	7180	6.46	1.57	1.40	1.25
RSV-B.S08	FW	7354	5.52	1.49	1.35	1.22
RSV-B.S08	RV	6847	5.37	1.48	1.34	1.22
SARS-CoV-2.S09	FW	5101	5.09	1.47	1.34	1.21
SARS-CoV-2.S09	RV	5378	5.19	1.48	1.34	1.22
gHEV.S10	FW	135372	17182.36	137.10	42.71	12.25
gHEV.S10	RV	120512	16845.38	144.75	44.65	12.63
gHEV.S11	FW	137986	17288.94	116.44	37.99	11.34
gHEV.S11	RV	118804	16544.89	119.59	38.79	11.50
gHEV.S12	FW	87365	7322.95	175.42	71.36	19.30
gHEV.S12	RV	74691	7276.66	180.70	72.86	19.54
gHEV.S13	FW	223705	171133.00	20304.17	3050.23	241.19
gHEV.S13	RV	178141	141520.90	21099.08	3243.87	255.46
gHEV.S14	FW	139407	37824.52	1027.08	287.49	47.76
gHEV.S14	RV	114751	35234.88	1149.31	320.07	51.78
gHEV.S15	FW	209683	123361.70	4933.76	780.53	87.05
gHEV.S15	RV	174583	108243.20	5156.54	819.08	90.92
gHEV.S16	FW	242079	198571.20	41197.29	6478.57	526.16
gHEV.S16	RV	200428	167029.70	39280.69	6284.56	516.14
gHEV.S17	FW	242171	156931.40	13670.95	3238.91	341.02
gHEV.S17	RV	193412	128034.70	12975.67	3156.40	346.09
gHEV.S18	FW	70195	63564.13	29435.46	6141.02	467.12
gHEV.S18	RV	55289	50685.19	26348.03	5936.40	492.73
gHEV.S19	FW	63350	48778.72	11344.69	3100.79	364.81
gHEV.S19	RV	51094	40722.29	11740.75	3295.52	353.09

Table S5: Hill numbers for each amplicon and strand at q=0,1,2,3 and infinity. Raw values.

ID	Strand	^1E	^2E	eInf
CoV-HKU1.S01	FW	0.0009	0.0003	0.0222
CoV-HKU1.S01	RV	0.0009	0.0002	0.0217
CoV-NL63.S02	FW	0.0012	0.0002	0.0308
CoV-NL63.S02	RV	0.0013	0.0002	0.0332
CoV-OC43.S03	FW	0.0009	0.0001	0.0352
CoV-OC43.S03	RV	0.0009	0.0001	0.0360
EV-A71.S04	FW	0.0012	0.0001	0.0447
EV-A71.S04	RV	0.0012	0.0001	0.0451
HCV-1B.S05	FW	0.0041	0.0002	0.1115
HCV-1B.S05	RV	0.0045	0.0002	0.1140
HMPV-B.S06	FW	0.0010	0.0001	0.0404
HMPV-B.S06	RV	0.0010	0.0001	0.0381
RSV-A.S07	FW	0.0009	0.0002	0.0257
RSV-A.S07	RV	0.0009	0.0002	0.0253
RSV-B.S08	FW	0.0008	0.0002	0.0223
RSV-B.S08	RV	0.0008	0.0002	0.0221
SARS-CoV-2.S09	FW	0.0010	0.0003	0.0226
SARS-CoV-2.S09	RV	0.0010	0.0003	0.0228
gHEV.S10	FW	0.1269	0.0010	0.2121
gHEV.S10	RV	0.1398	0.0012	0.2167
gHEV.S11	FW	0.1253	0.0008	0.2052
gHEV.S11	RV	0.1393	0.0010	0.2090
gHEV.S12	FW	0.0838	0.0020	0.2602
gHEV.S12	RV	0.0974	0.0024	0.2649
gHEV.S13	FW	0.7650	0.0908	0.4453
gHEV.S13	RV	0.7944	0.1184	0.4585
gHEV.S14	FW	0.2713	0.0074	0.3264
gHEV.S14	RV	0.3071	0.0100	0.3388
gHEV.S15	FW	0.5883	0.0235	0.3645
gHEV.S15	RV	0.6200	0.0295	0.3737
gHEV.S16	FW	0.8203	0.1702	0.5054
gHEV.S16	RV	0.8334	0.1960	0.5117
gHEV.S17	FW	0.6480	0.0565	0.4704
gHEV.S17	RV	0.6620	0.0671	0.4803
gHEV.S18	FW	0.9055	0.4193	0.5508
gHEV.S18	RV	0.9167	0.4766	0.5677
gHEV.S19	FW	0.7700	0.1791	0.5336
gHEV.S19	RV	0.7970	0.2298	0.5411

Table S6: Evenness for each amplicon and strand, at q = 1 and 2, $^qE = {^qD}/{^0D}$, and $eInf = log({^\infty D})/log({^0D})$. Raw values.

	Flat	Other
Min.	0.0838	0.000751
1st Qu.	0.1400	0.000901
Median	0.6340	0.000966
Mean	0.5210	0.001350
3rd Qu.	0.7950	0.001210
Max.	0.9170	0.004530

Table S7: Summary values of Hill evenness at q 1, ${}^{1}E = {}^{1}D/{}^{0}D$, for flat-like versus regular quasispecies.

	Flat	Other
Min.	0.205	0.0217
1st Qu.	0.264	0.0227
Median	0.409	0.0320
Mean	0.392	0.0397
3rd Qu.	0.507	0.0399
Max.	0.568	0.1140

Table S8: Summary values of evenness at q infinity, $eInf = log({}^{\infty}D)/log({}^{0}D)$, for flat-like versus regular quasispecies.

ID	Strand	Top25	Ratio
CoV-HKU1.S01	FW	85.207	1.032
CoV-HKU1.S01	RV	85.528	1.033
CoV-NL63.S02	FW	79.002	1.043
CoV-NL63.S02	RV	77.230	1.046
CoV-OC43.S03	FW	74.356	1.048
CoV-OC43.S03	RV	73.995	1.051
EV-A71.S04	FW	68.690	1.075
EV-A71.S04	RV	68.378	1.076
HCV-1B.S05	FW	60.026	1.911
HCV-1B.S05	RV	59.451	1.933
HMPV-B.S06	FW	72.480	1.083
HMPV-B.S06	RV	74.398	1.081
RSV-A.S07	FW	83.122	1.044
RSV-A.S07	RV	83.303	1.043
RSV-B.S08	FW	84.349	1.029
RSV-B.S08	RV	84.524	1.028
SARS-CoV-2.S09	FW	85.721	1.040
SARS-CoV-2.S09	RV	85.406	1.039
gHEV.S10	FW	16.670	2.042
gHEV.S10	RV	16.414	2.072
gHEV.S11	FW	19.128	2.169
gHEV.S11	RV	18.801	2.162
gHEV.S12	FW	25.118	4.848
gHEV.S12	RV	24.592	4.805
gHEV.S13	FW	2.177	5.250
gHEV.S13	RV	2.147	5.486
gHEV.S14	FW	10.932	5.221
gHEV.S14	RV	10.317	5.342
gHEV.S15	FW	3.911	3.404
gHEV.S15	RV	3.761	3.420
gHEV.S16	FW	1.541	8.106
gHEV.S16	RV	1.502	7.751
gHEV.S17	FW	3.384	11.539
gHEV.S17	RV	3.327	11.514
gHEV.S18	FW	1.530	7.148
gHEV.S18	RV	1.497	7.375
gHEV.S19	FW	3.579	13.055
gHEV.S19	RV	3.249	11.473

Table S9: Fraction of reads of the 25 most abundant haplotypes in each amplicon and strand, expressed as percentage, and ratio of this fraction to the corresponding master frequency.

	Flat	Other
Min.	2.042	1.028
1st Qu.	3.416	1.039
Median	5.296	1.045
Mean	6.209	1.146
3rd Qu.	7.840	1.076
Max.	13.055	1.933

Table S10: Summary values of the ratio of top 25 haplotypes versus master haplotype.

ID	FW	RV
CoV-HKU1.S01	0.181	0.179
CoV-NL63.S02	0.275	0.297
CoV-OC43.S03	0.332	0.339
EV-A71.S04	0.441	0.448
HCV-1B.S05	0.985	1.006
HMPV-B.S06	0.388	0.359
RSV-A.S07	0.213	0.210
RSV-B.S08	0.198	0.194
SARS-CoV-2.S09	0.158	0.162
gHEV.S10	2.520	2.553
gHEV.S11	2.420	2.459
gHEV.S12	4.200	4.226
gHEV.S13	5.490	5.542
gHEV.S14	4.418	4.518
gHEV.S15	5.669	5.765
gHEV.S16	7.528	7.607
gHEV.S17	6.753	6.821
gHEV.S18	7.554	7.213
gHEV.S19	6.709	6.847

Table S11: Average number of substitutions with respect to the master haplotype, per read, for each amplicon and strand. FW: forward strand, RV: reverse strand.

ID	Strand	q	Raw	0.5	0.25	0.1	0.05
gHEV.S16	FW	0	242079	125377	64368	26432	13413
gHEV.S16	FW	1	198571	108815	58346	24956	12925
gHEV.S16	FW	2	41197	35870	28577	17704	10804
gHEV.S16	FW	3	6479	6443	6401	6156	5568
gHEV.S16	FW	inf	526	526	526	512	488
gHEV.S16	RV	0	200428	103381	52950	21702	11006
gHEV.S16	RV	1	167030	90825	48455	20619	10657
gHEV.S16	RV	2	39281	33481	25924	15394	9192
gHEV.S16	RV	3	6285	6246	6188	5868	5214
gHEV.S16	RV	inf	516	515	510	489	470
gHEV.S17	FW	0	242171	126825	65779	27321	13969
gHEV.S17	FW	1	156931	90668	51019	23090	12421
gHEV.S17	FW	2	13671	13083	12053	9757	7401
gHEV.S17	FW	3	3239	3226	3204	3134	3012
gHEV.S17	FW	inf	341	341	342	344	348
gHEV.S17	RV	0	193412	100853	52135	21586	11020
gHEV.S17	RV	1	128035	73346	41005	18458	9901
gHEV.S17	RV	2	12976	12321	11164	8760	6420
gHEV.S17	RV	3	3156	3146	3113	3039	2870
gHEV.S17	RV	inf	346	347	347	348	342

Table S12: Hill numbers of different orders q, ${}^{q}D$, as observed (raw) and as computed at different subsampling fractions by rarefaction.

ID	Strand	Subsz	${}^{1}E$	${}^{2}E$	${}^{3}E$	${}^{\infty}E$	eInf
gHEV.S16	FW	Raw	0.8203	0.1702	0.0268	0.0022	0.5054
gHEV.S16	FW	0.5	0.8679	0.2861	0.0514	0.0042	0.5337
gHEV.S16	FW	0.25	0.9065	0.444	0.0994	0.0082	0.5659
gHEV.S16	FW	0.1	0.9442	0.6698	0.2329	0.0194	0.6127
gHEV.S16	FW	0.05	0.9636	0.8055	0.4151	0.0364	0.6514
gHEV.S16	RV	Raw	0.8334	0.196	0.0314	0.0026	0.5116
gHEV.S16	RV	0.5	0.8785	0.3239	0.0604	0.005	0.5408
gHEV.S16	RV	0.25	0.9151	0.4896	0.1169	0.0096	0.5731
gHEV.S16	RV	0.1	0.9501	0.7093	0.2704	0.0226	0.6202
gHEV.S16	RV	0.05	0.9683	0.8352	0.4737	0.0427	0.6612
gHEV.S17	FW	Raw	0.648	0.0565	0.0134	0.0014	0.4704
gHEV.S17	FW	0.5	0.7149	0.1032	0.0254	0.0027	0.4963
gHEV.S17	FW	0.25	0.7756	0.1832	0.0487	0.0052	0.5259
gHEV.S17	FW	0.1	0.8451	0.3571	0.1147	0.0126	0.5717
gHEV.S17	FW	0.05	0.8892	0.5298	0.2156	0.0249	0.6131
gHEV.S17	RV	Raw	0.662	0.0671	0.0163	0.0018	0.4803
gHEV.S17	RV	0.5	0.7273	0.1222	0.0312	0.0034	0.5076
gHEV.S17	RV	0.25	0.7865	0.2141	0.0597	0.0066	0.5384
gHEV.S17	RV	0.1	0.8551	0.4058	0.1408	0.0161	0.5863
gHEV.S17	RV	0.05	0.8985	0.5826	0.2604	0.0311	0.627

Table S13: Evenness of different orders q, ${}^{q}E = {}^{q}D/{}^{0}D$, and $eInf = log({}^{\infty}D)/log({}^{0}D)$, as observed (raw) and as computed at different subsampling fractions by rarefaction.

ID	Strand	Master	Emerging	0.1 − 1%	< 0.1%
CoV-HKU1.S01	FW	82.532	0.000	1.689	15.791
CoV-HKU1.S01	RV	82.826	0.000	1.717	15.467
CoV-NL63.S02	FW	75.754	0.000	4.637	19.589
CoV-NL63.S02	RV	73.846	0.000	5.459	20.704
CoV-OC43.S03	FW	70.943	0.000	5.992	23.073
CoV-OC43.S03	RV	70.384	0.000	6.386	23.229
EV-A71.S04	FW	63.898	0.000	13.693	22.407
EV-A71.S04	RV	63.577	0.000	14.016	22.411
HCV-1B.S05	FW	31.409	22.369	10.475	35.747
HCV-1B.S05	RV	30.747	21.891	11.472	35.889
HMPV-B.S06	FW	66.941	0.000	7.636	25.412
HMPV-B.S06	RV	68.837	0.000	6.504	24.652
RSV-A.S07	FW	79.639	0.000	3.039	17.305
RSV-A.S07	RV	79.846	0.000	2.876	17.295
RSV-B.S08	FW	81.977	0.000	1.018	17.029
RSV-B.S08	RV	82.230	0.000	0.964	16.837
SARS-CoV-2.S09	FW	82.435	0.000	2.292	15.288
SARS-CoV-2.S09	RV	82.225	0.000	2.125	15.650
gHEV.S10	FW	8.164	1.305	12.358	78.177
gHEV.S10	RV	7.921	1.364	12.035	78.674
gHEV.S11	FW	8.818	2.639	9.878	78.661
gHEV.S11	RV	8.695	2.772	9.364	79.168
gHEV.S12	FW	5.183	12.110	15.040	67.658
gHEV.S12	RV	5.118	12.102	15.063	67.718
gHEV.S13	FW	0.415	0.000	0.901	98.684
gHEV.S13	RV	0.391	0.000	0.787	98.822
gHEV.S14	FW	2.094	1.055	10.996	85.852
gHEV.S14	RV	1.931	0.000	12.383	85.686
gHEV.S15	FW	1.149	0.000	1.550	97.298
gHEV.S15	RV	1.100	0.000	1.519	97.381
gHEV.S16	FW	0.190	0.000	0.627	99.183
gHEV.S16	RV	0.194	0.000	0.640	99.166
gHEV.S17	FW	0.293	0.000	2.643	97.064
gHEV.S17	RV	0.289	0.000	2.380	97.331

Table S14: Quasispecies Fitness Fractions rarefied to 194,000 reads. Expressed as percentages.

ID	Strand	^0D	^1D	^2D	^3D	$^\infty D$
CoV-HKU1.S01	FW	4440	5.21	1.47	1.33	1.21
CoV-HKU1.S01	RV	4435	5.10	1.46	1.33	1.21
CoV-NL63.S02	FW	7643	9.58	1.74	1.52	1.32
CoV-NL63.S02	RV	8688	11.45	1.83	1.58	1.35
CoV-OC43.S03	FW	10058	14.58	1.99	1.67	1.41
CoV-OC43.S03	RV	10298	15.26	2.02	1.69	1.42
EV-A71.S04	FW	14010	26.36	2.45	1.96	1.57
EV-A71.S04	RV	14417	27.35	2.47	1.97	1.57
HCV-1B.S05	FW	23048	125.40	7.01	5.00	3.18
HCV-1B.S05	RV	23756	136.42	7.32	5.16	3.25
HMPV-B.S06	FW	12167	20.24	2.23	1.83	1.49
HMPV-B.S06	RV	10856	16.80	2.11	1.75	1.45
RSV-A.S07	FW	5459	6.53	1.58	1.41	1.26
RSV-A.S07	RV	5370	6.41	1.57	1.40	1.25
RSV-B.S08	FW	4636	5.45	1.49	1.35	1.22
RSV-B.S08	RV	4413	5.31	1.48	1.34	1.22
SARS-CoV-2.S09	FW	4050	5.06	1.47	1.34	1.21
SARS-CoV-2.S09	RV	4153	5.16	1.48	1.34	1.22
gHEV.S10	FW	92129	14155.20	137.18	42.74	12.26
gHEV.S10	RV	93428	14776.49	144.66	44.65	12.62
gHEV.S11	FW	96334	14313.91	116.41	37.99	11.34
gHEV.S11	RV	97213	14863.67	119.57	38.78	11.50
gHEV.S12	FW	72717	6790.57	175.40	71.37	19.30
gHEV.S12	RV	74691	7276.66	180.70	72.86	19.54
gHEV.S13	FW	166874	131998.40	19768.23	3053.16	241.46
gHEV.S13	RV	167949	134246.49	20961.80	3244.49	255.43
gHEV.S14	FW	112186	33082.49	1025.97	287.43	47.75
gHEV.S14	RV	114751	35234.88	1149.31	320.07	51.78
gHEV.S15	FW	151854	95004.68	4901.27	780.43	87.05
gHEV.S15	RV	153152	97214.24	5133.48	818.22	90.89
gHEV.S16	FW	172188	145705.60	38765.73	6476.78	526.10
gHEV.S16	RV	172311	145525.42	38160.41	6279.67	516.30
gHEV.S17	FW	159118	110153.38	13327.44	3233.41	340.58
gHEV.S17	RV	158740	108329.62	12808.58	3152.04	345.43

Table S15: Hill numbers ^qD at $q = 1, 2, 3$, and ∞, rarefied to 194,000 reads.

ID	Strand	$RLE_{0,1}$	$RLE_{0,2}$	$RLE_{0,3}$	$RLE_{0,\infty}$
CoV-HKU1.S01	FW	0.197	0.046	0.034	0.023
CoV-HKU1.S01	RV	0.194	0.045	0.034	0.022
CoV-NL63.S02	FW	0.253	0.062	0.047	0.031
CoV-NL63.S02	RV	0.269	0.067	0.050	0.033
CoV-OC43.S03	FW	0.291	0.074	0.056	0.037
CoV-OC43.S03	RV	0.295	0.076	0.057	0.038
EV-A71.S04	FW	0.343	0.094	0.070	0.047
EV-A71.S04	RV	0.346	0.095	0.071	0.047
HCV-1B.S05	FW	0.481	0.194	0.160	0.115
HCV-1B.S05	RV	0.488	0.198	0.163	0.117
HMPV-B.S06	FW	0.320	0.085	0.064	0.043
HMPV-B.S06	RV	0.304	0.080	0.060	0.040
RSV-A.S07	FW	0.218	0.053	0.040	0.026
RSV-A.S07	RV	0.216	0.052	0.039	0.026
RSV-B.S08	FW	0.201	0.047	0.035	0.024
RSV-B.S08	RV	0.199	0.047	0.035	0.023
SARS-CoV-2.S09	FW	0.195	0.046	0.035	0.023
SARS-CoV-2.S09	RV	0.197	0.047	0.035	0.023
gHEV.S10	FW	0.836	0.431	0.329	0.219
gHEV.S10	RV	0.839	0.435	0.332	0.222
gHEV.S11	FW	0.834	0.415	0.317	0.212
gHEV.S11	RV	0.836	0.417	0.319	0.213
gHEV.S12	FW	0.788	0.462	0.381	0.264
gHEV.S12	RV	0.792	0.463	0.382	0.265
gHEV.S13	FW	0.981	0.823	0.667	0.456
gHEV.S13	RV	0.981	0.827	0.672	0.461
gHEV.S14	FW	0.895	0.596	0.487	0.332
gHEV.S14	RV	0.899	0.605	0.495	0.339
gHEV.S15	FW	0.961	0.712	0.558	0.374
gHEV.S15	RV	0.962	0.716	0.562	0.378
gHEV.S16	FW	0.986	0.876	0.728	0.520
gHEV.S16	RV	0.986	0.875	0.725	0.518
gHEV.S17	FW	0.969	0.793	0.675	0.487
gHEV.S17	RV	0.968	0.790	0.673	0.488

Table S16: Relative logarithmic evenness, $RLE_{0,q} = log(^qD)/log(^0D)$, at $q = 1, 2, 3$, and ∞, rarefied to 194,000 reads.

3 Supplementary Figures

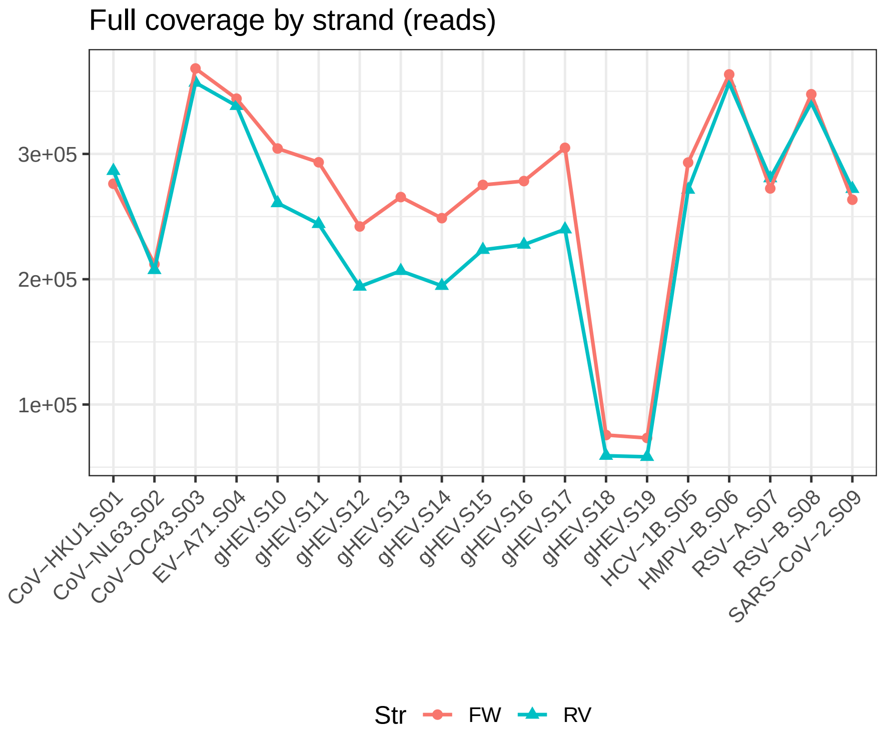

Figure S1: Coverage as number of reads per amplicon and strand. Orange: forward strand, turquoise: reverse strand.

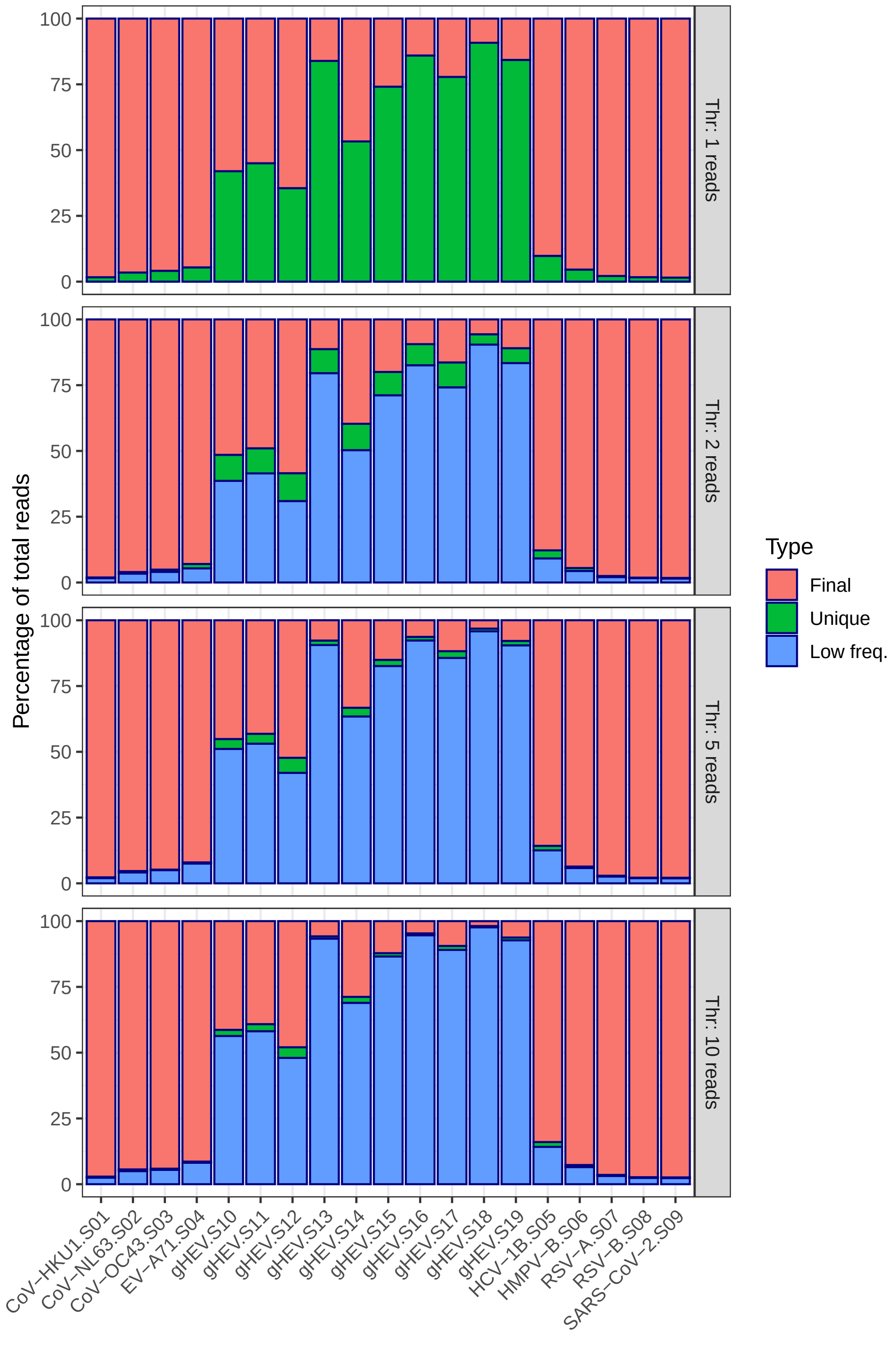

Figure S2: Results of previous abundance filter at a given number of reads per haplotype, followed by strand intersection. Final: fraction of reads resulting from the filter and intersection; Unique: fraction of reads corresponding to haplotypes passing the abundance filter but being unique to either strand; Low freq: fraction of reads corresponding to haplotypes not passing the abundance filter.

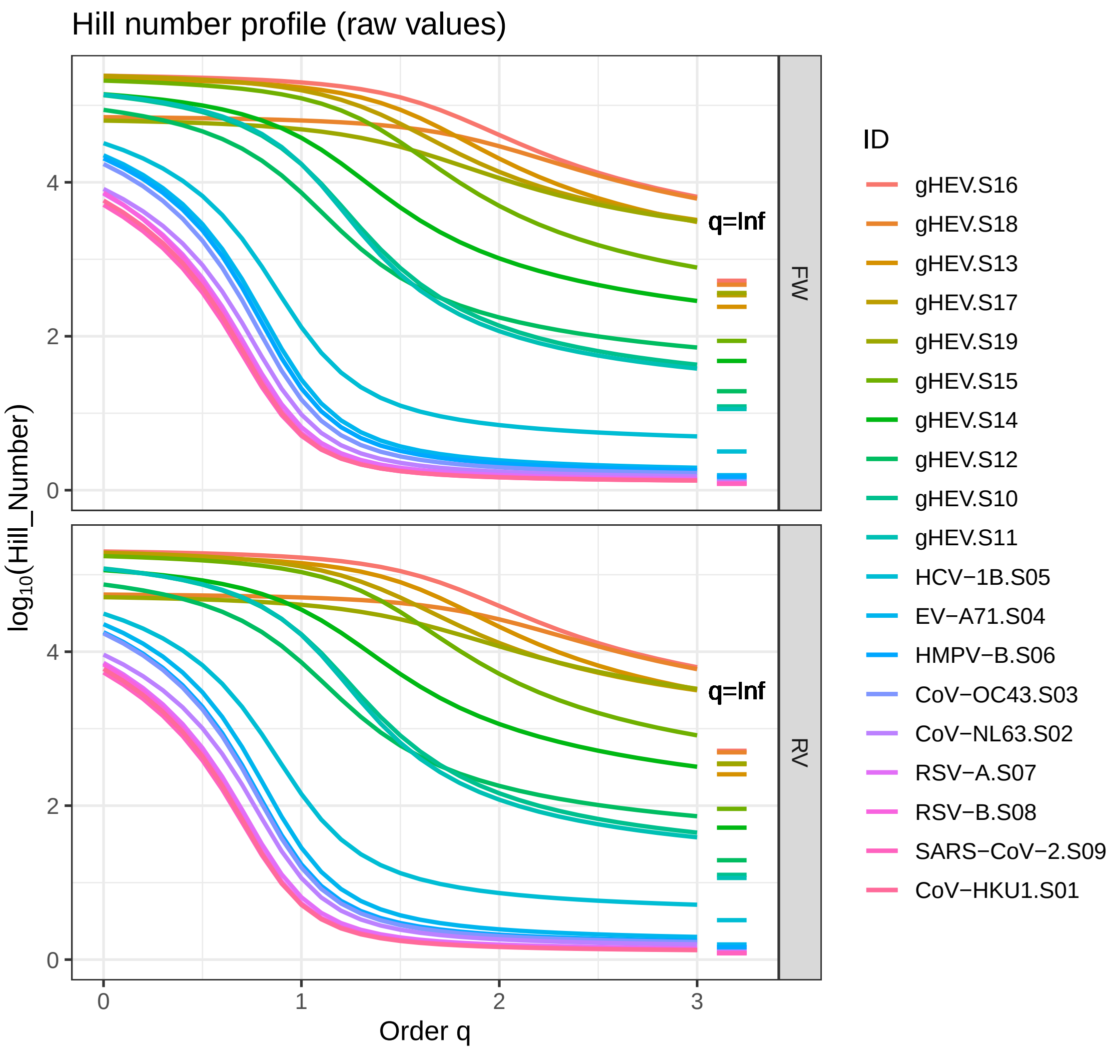

Figure S3: Hill numbers profile of flat-like vs. regular quasispecies. The legends are sorted in decreasing values at q=2.

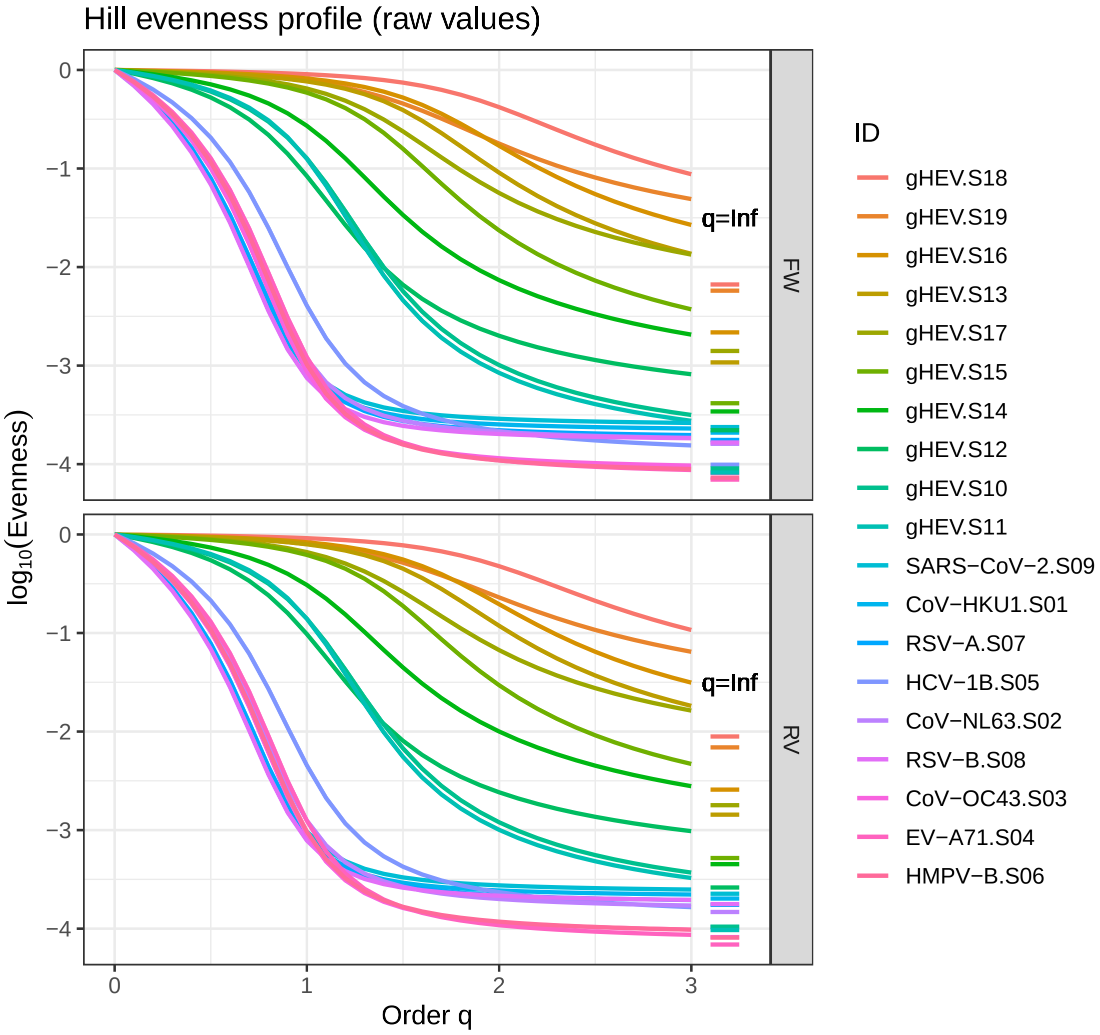

Figure S4: Hill evenness profile of flat-like vs. regular quasispecies. The legends are sorted in decreasing values at q=2.

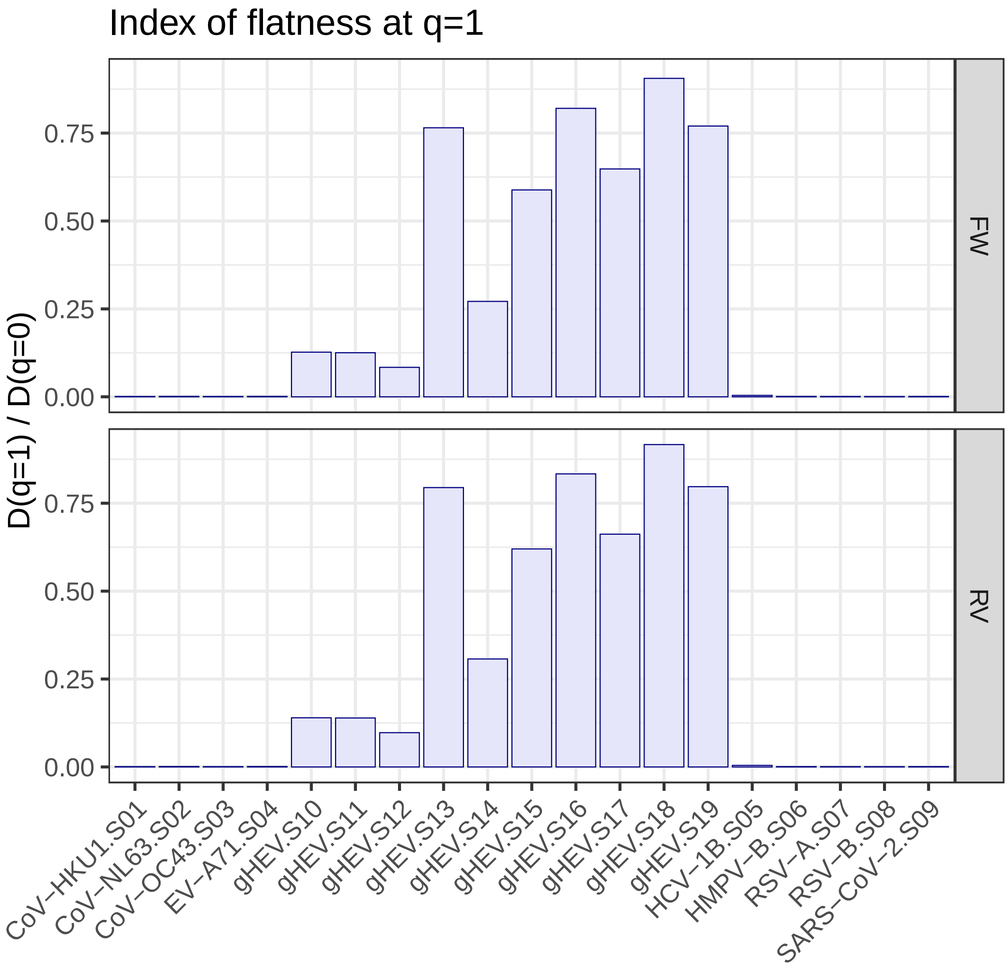

Figure S5: Index of evenness at q=1.

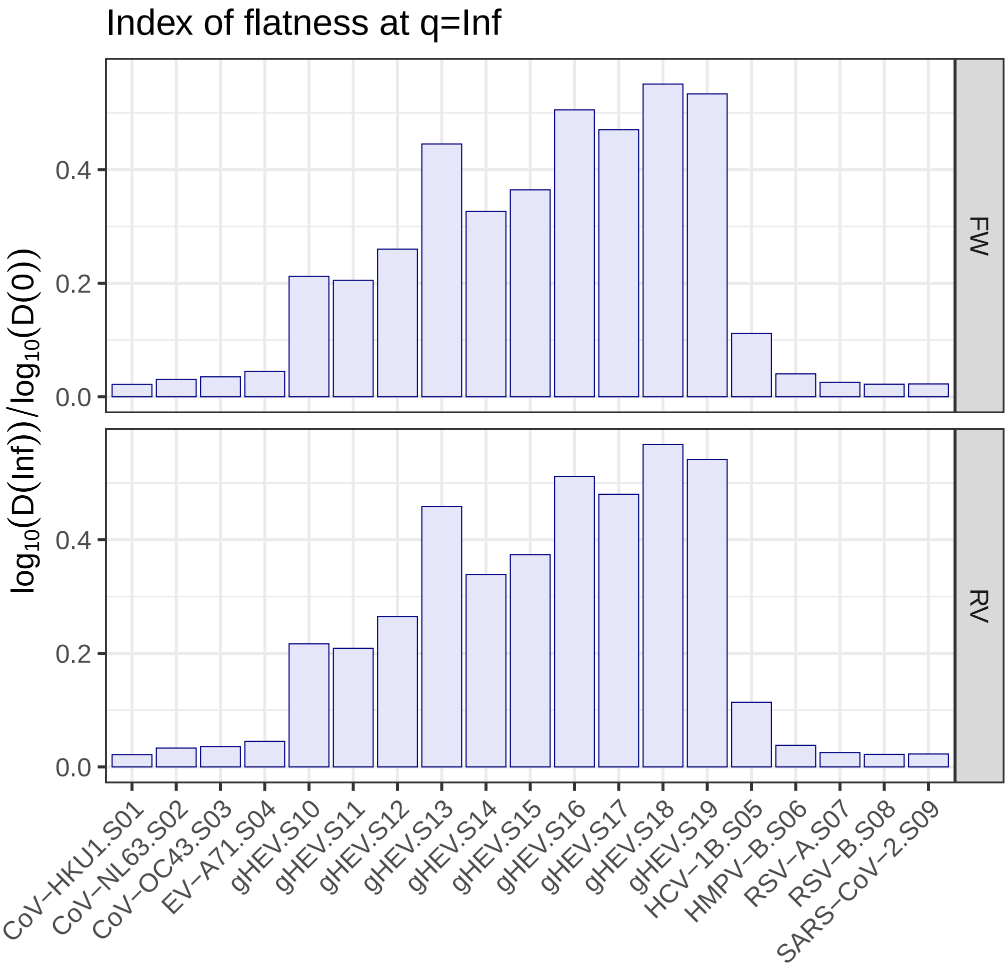

Figure S6: Index of evenness at q=infinity.

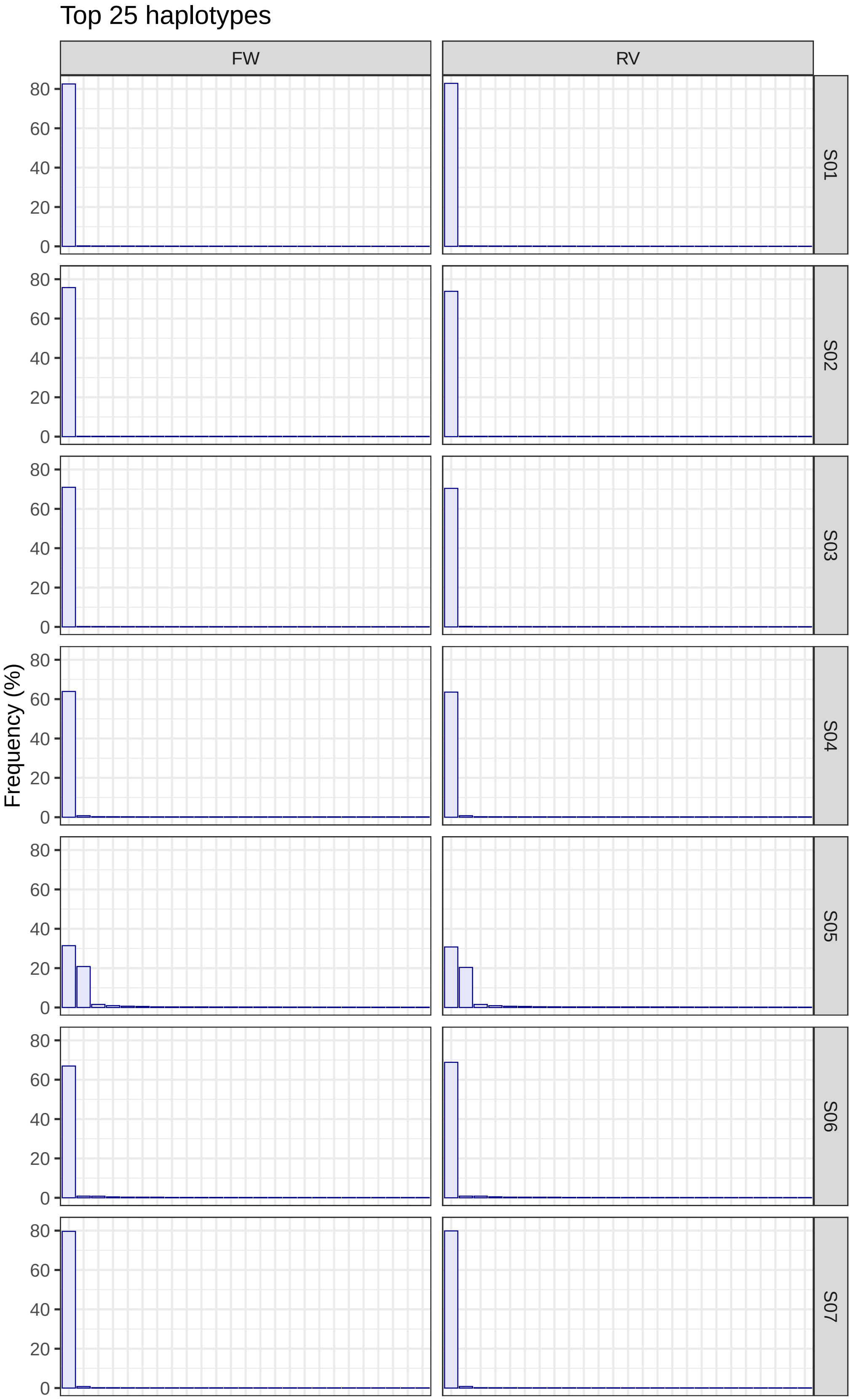
Top 25 haplotypes
FW
RV
S01
S02
S03
S04
S05
S06
S07
Frequency (%)
80
60
40
20
0

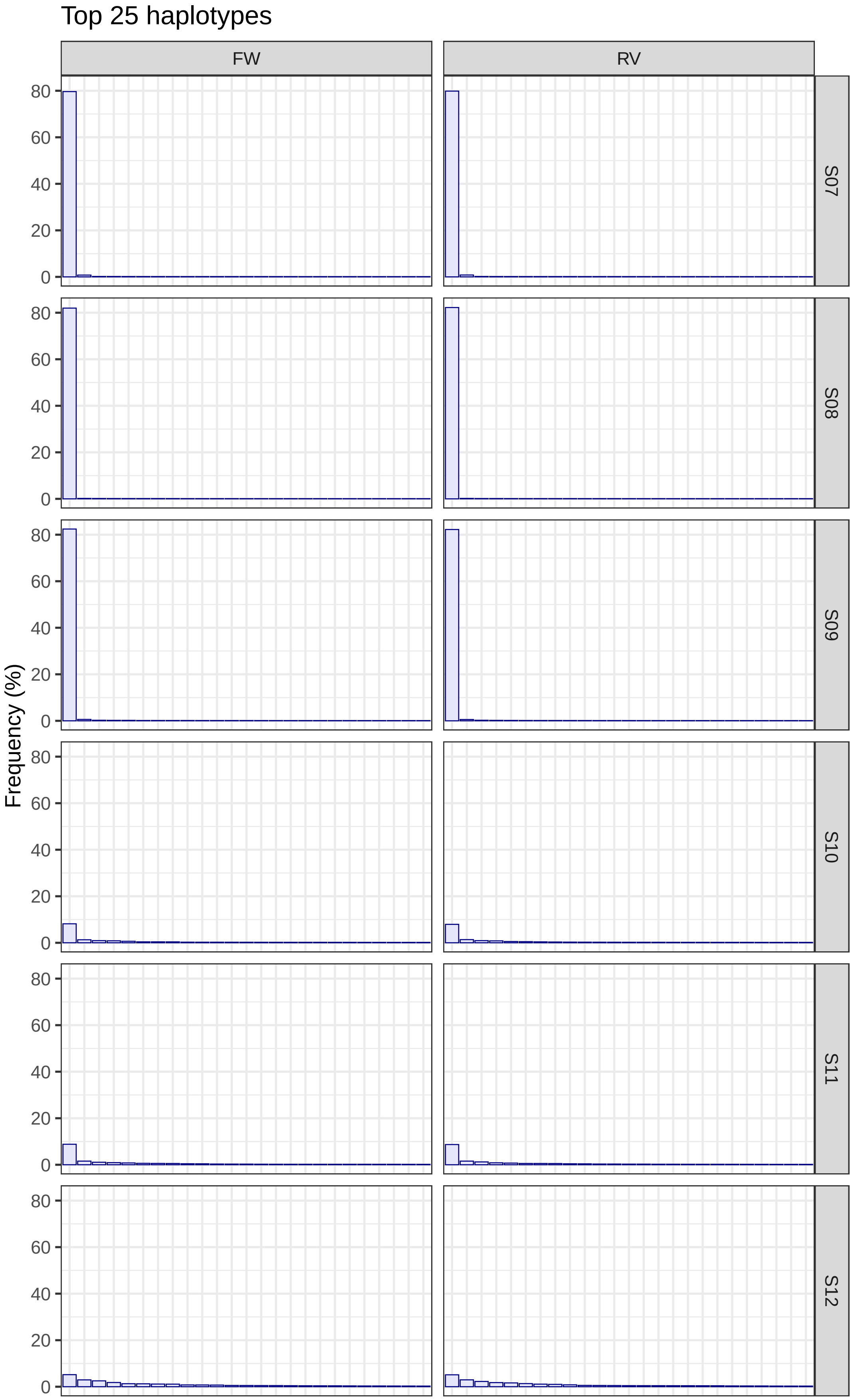
Top 25 haplotypes
FW
RV
S07
S08
S09
S10
S11
S12
Frequency (%)
0
20
40
60
80

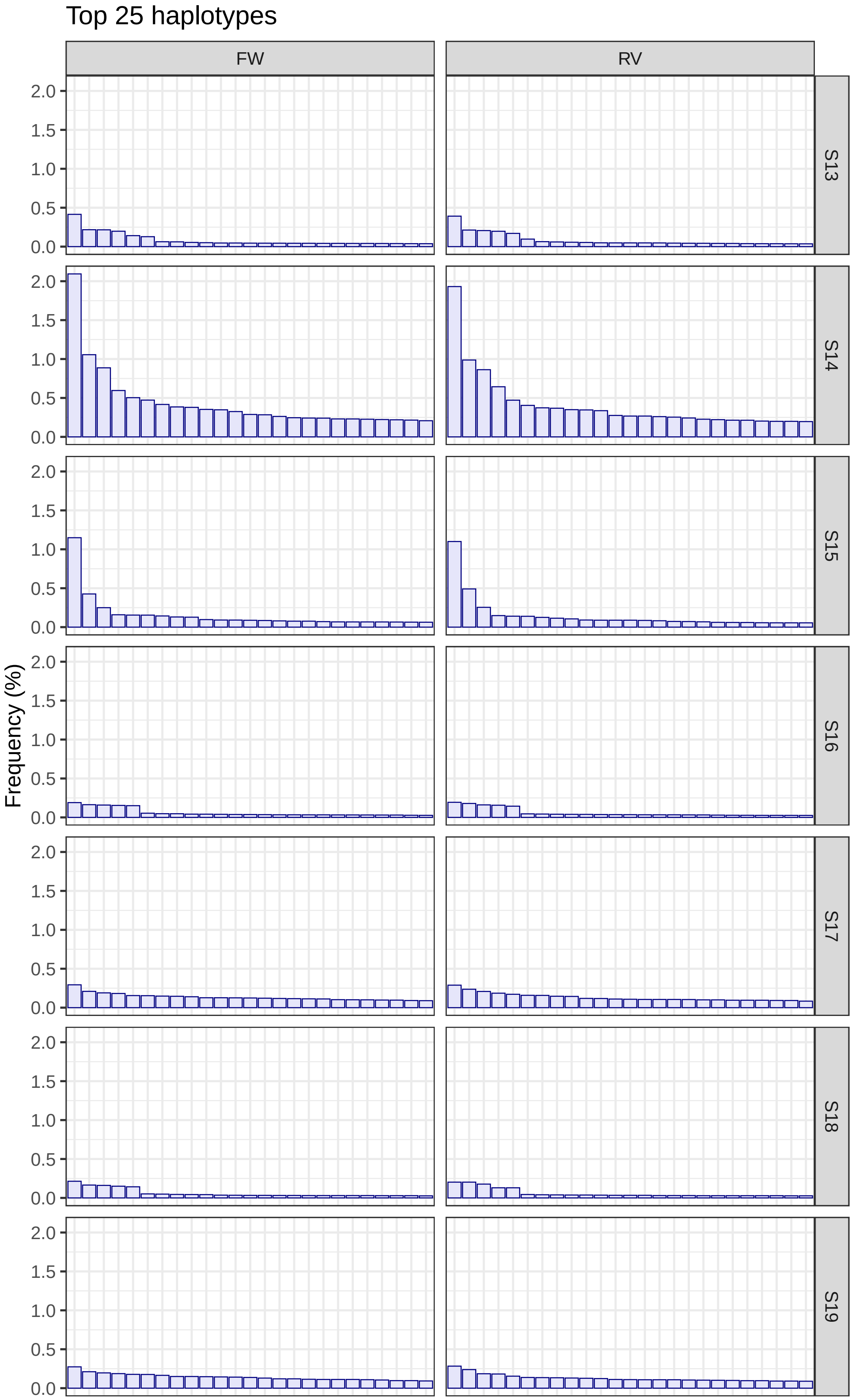

Figure S7: Frequencies of top 25 haplotypes in decreasing order. Note the scale change in the frequency axis, for samples S13 to S19, to improve readability.

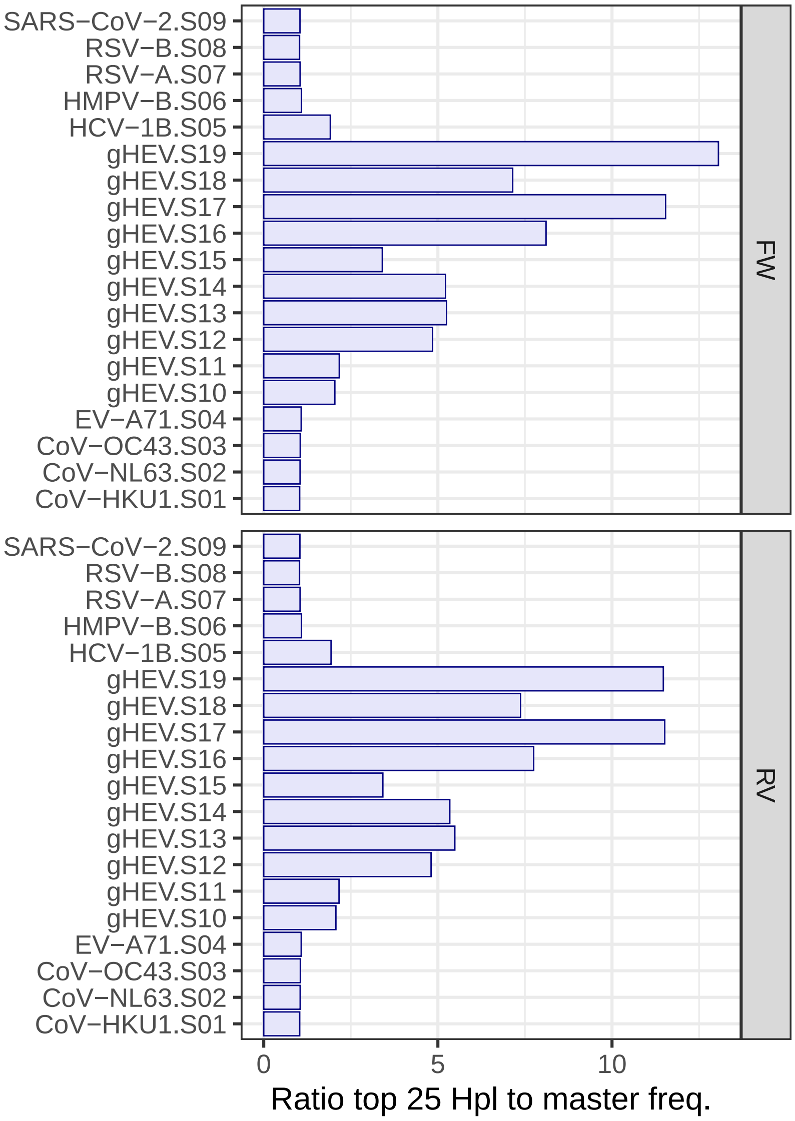

Figure S8: Ratio of the fraction of reads accounting for the top 25 most frequent haplotypes to the master haplotype.

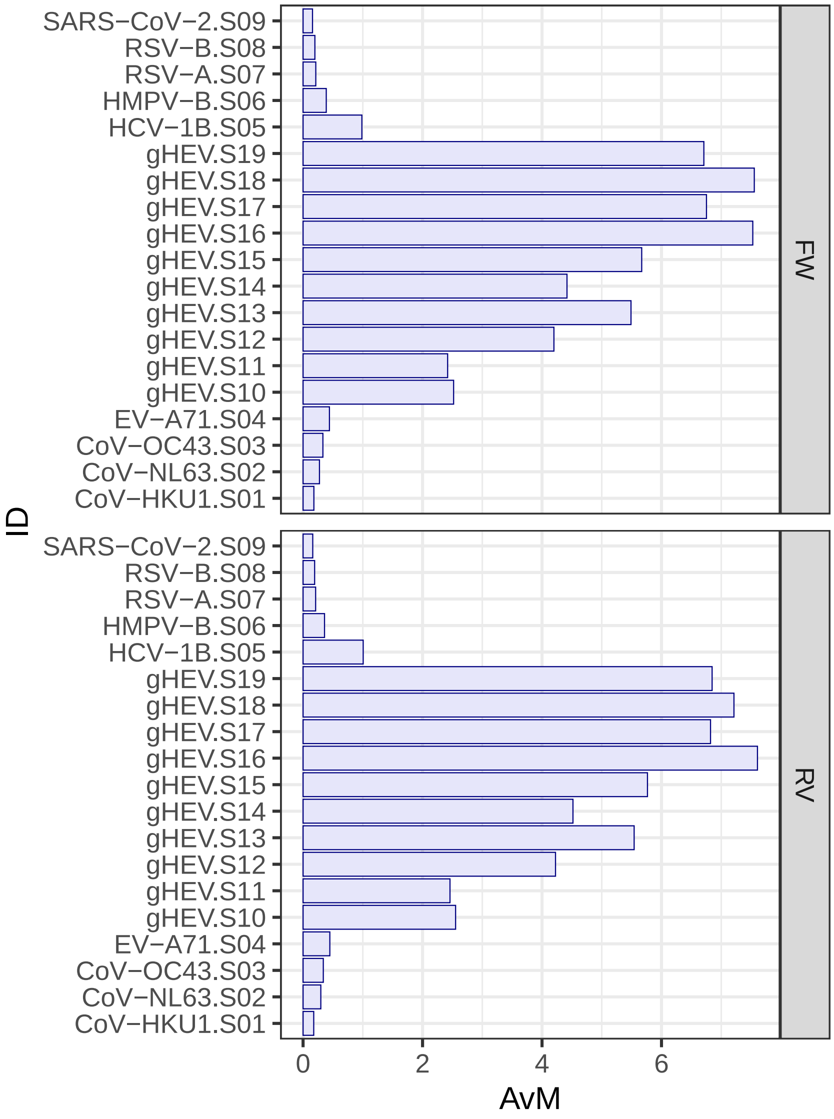

Figure S9: Average number of substitutions per read relative to the master haplotype.

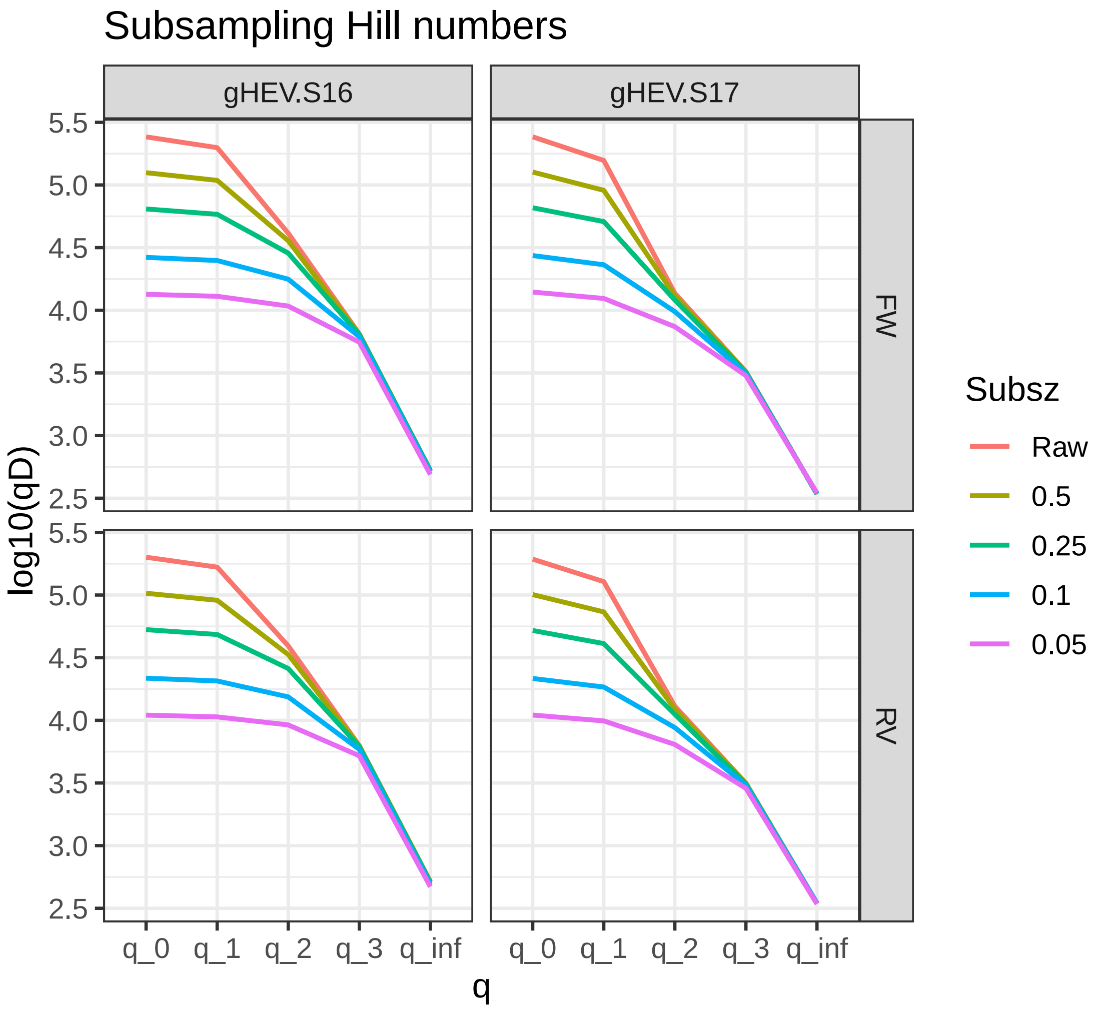

Figure S10: Effect of rarefaction on Hill numbers of different order q. The sensitivity of Hill numbers decreases as q increases, being q=0 the most sensitive, and with q over 3 almost insensitive. Subsz: fraction of subsampling.

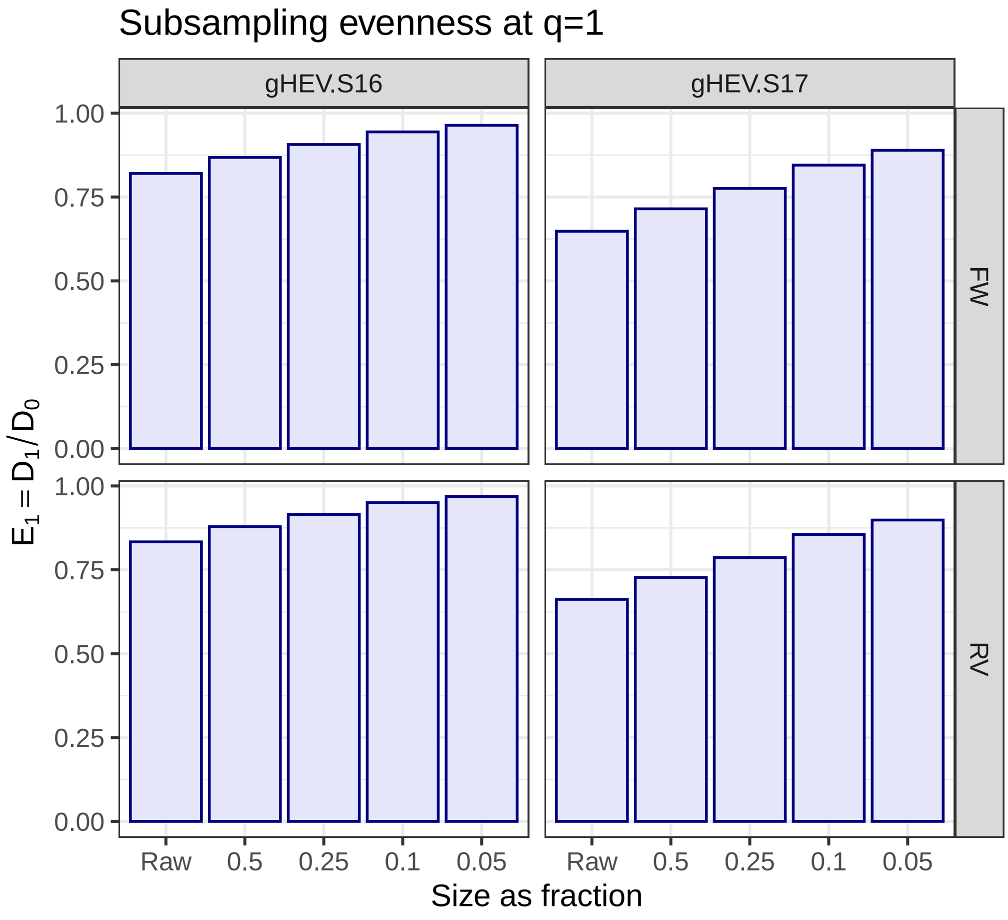

Figure S11: Effect of rarefaction on evenness ${}^1E = {}^1D/{}^0D$. Decreasing fractions result in increasing values of evenness.

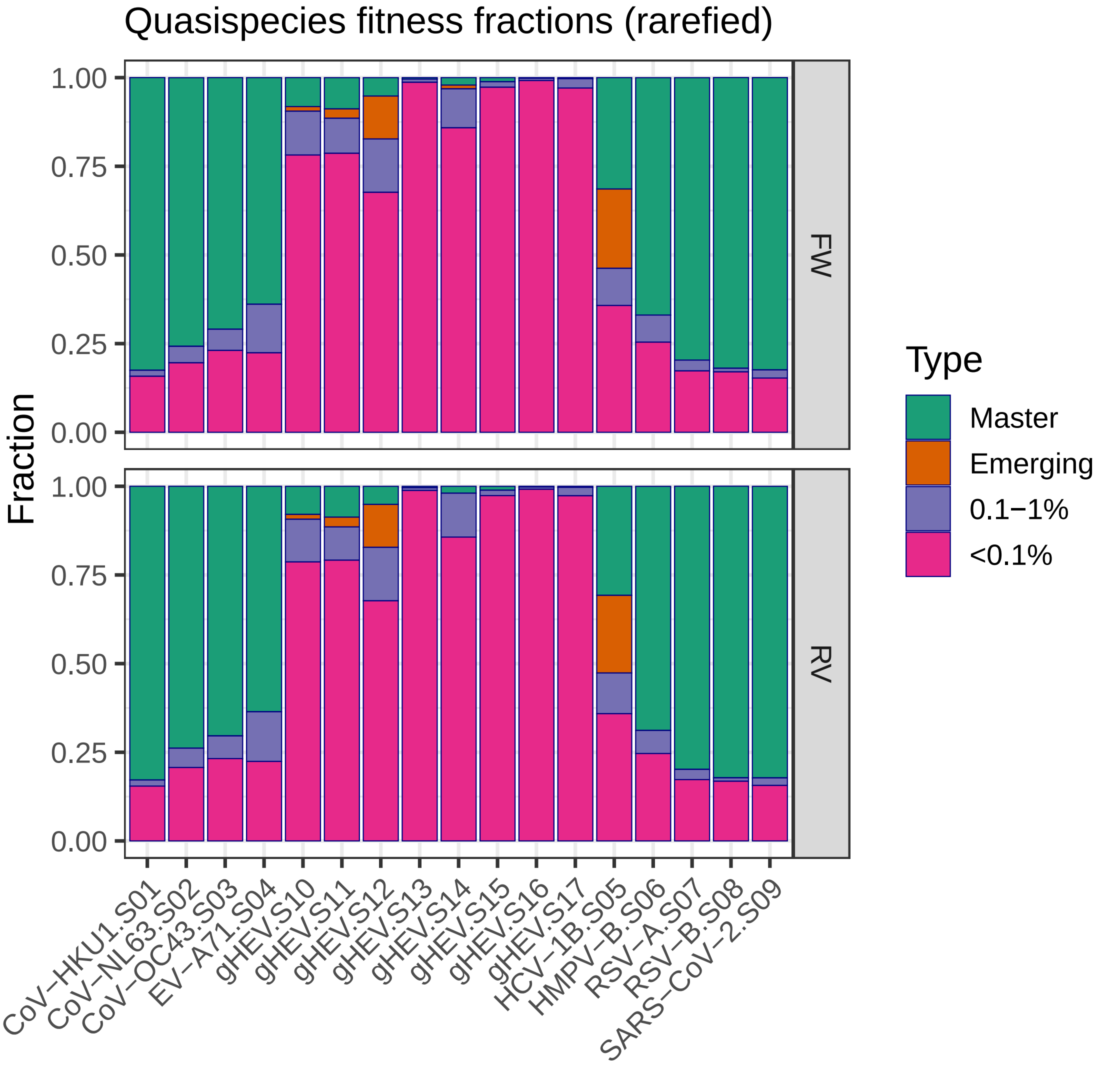

Figure S12: Quasispecies Fitness Fractions, rarefied to 194,000 reads.

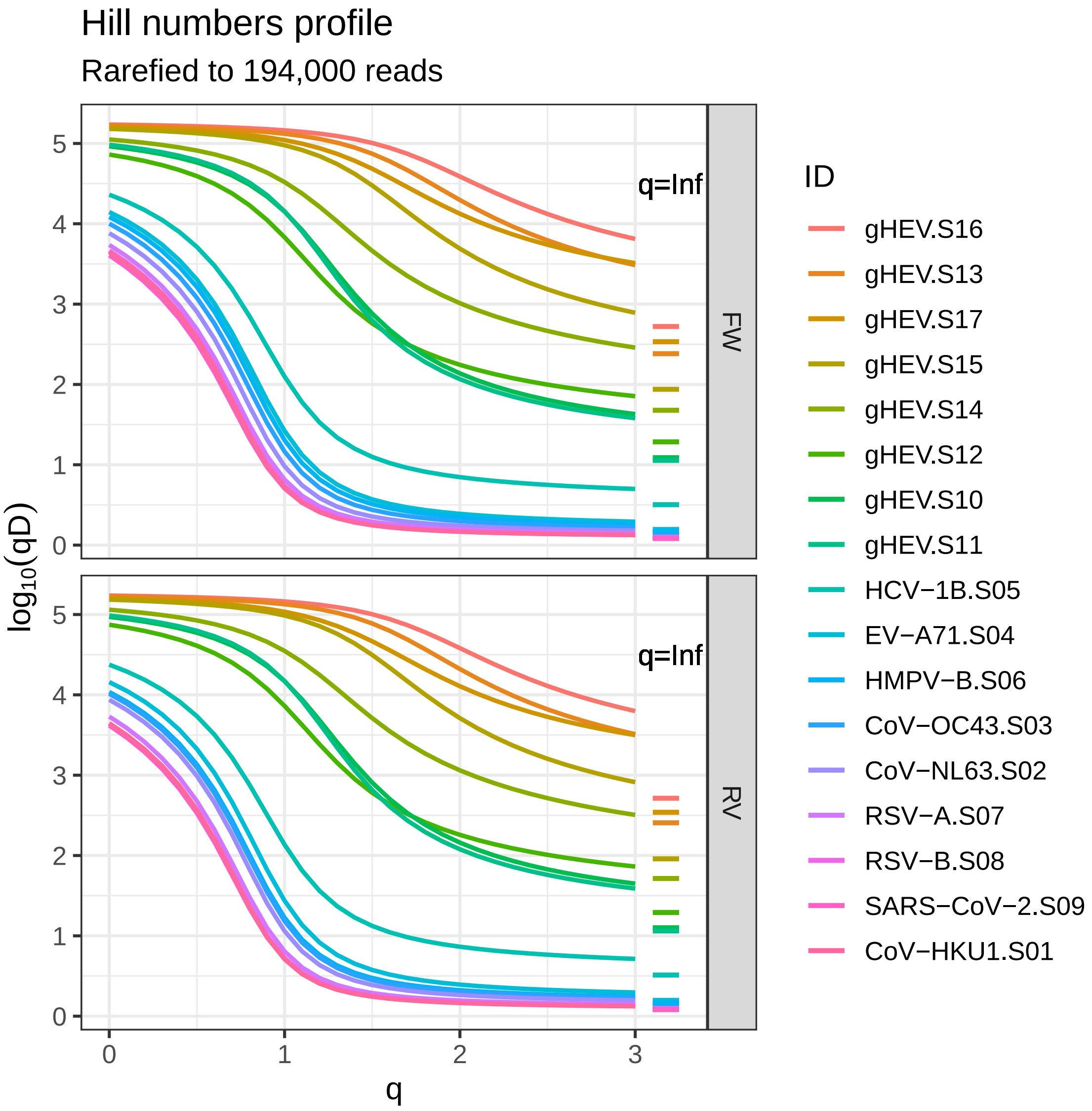

Figure S13: Rarefied Hill numbers profiles. Labels sorted in decreasing order of ${}^2D$ at $q = 2$.

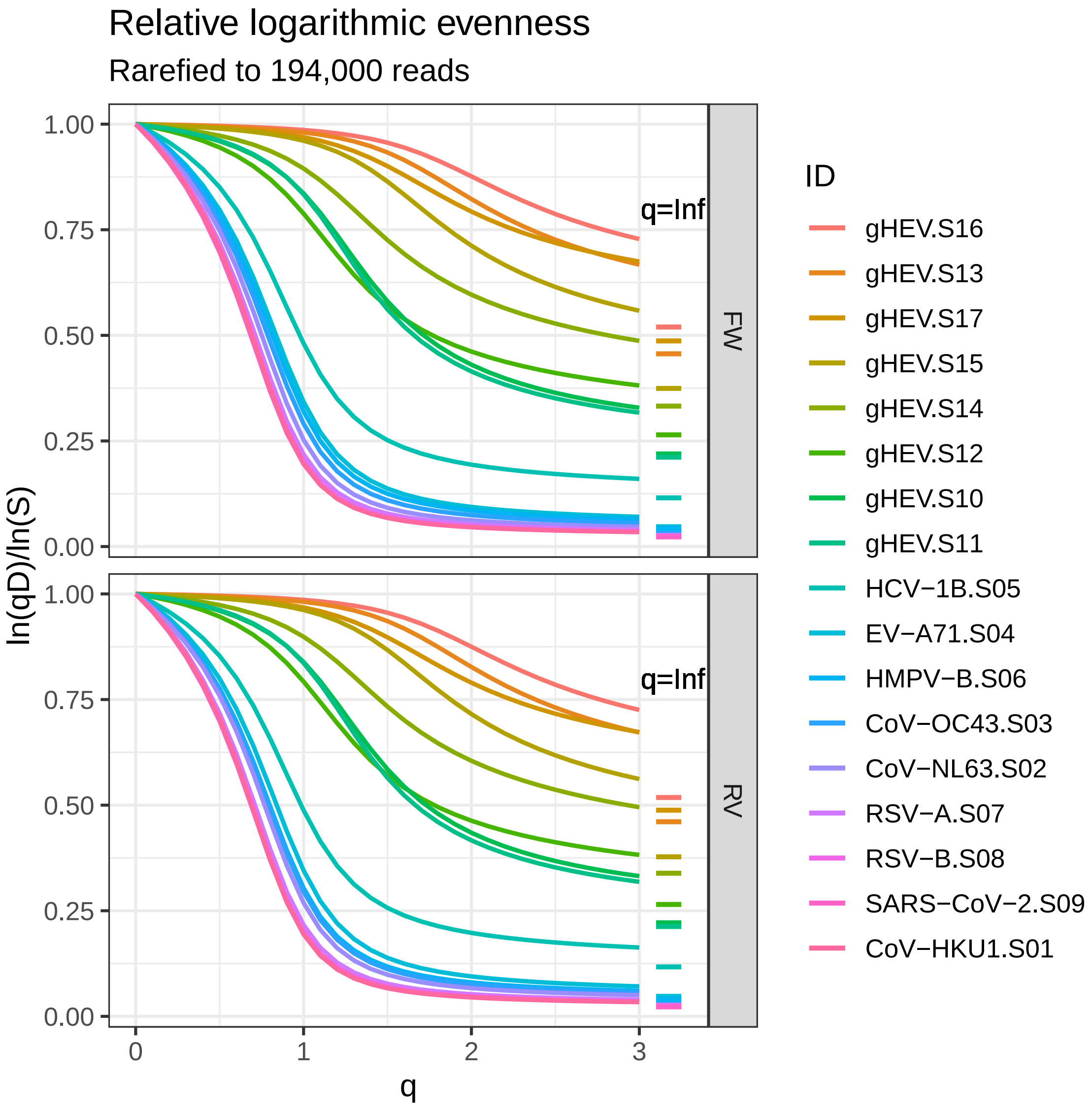

Figure S14: Rarefied relative logarithmic evennes profile. Labels sorted in decreasing order of ${}^2D$ at $q = 2$.

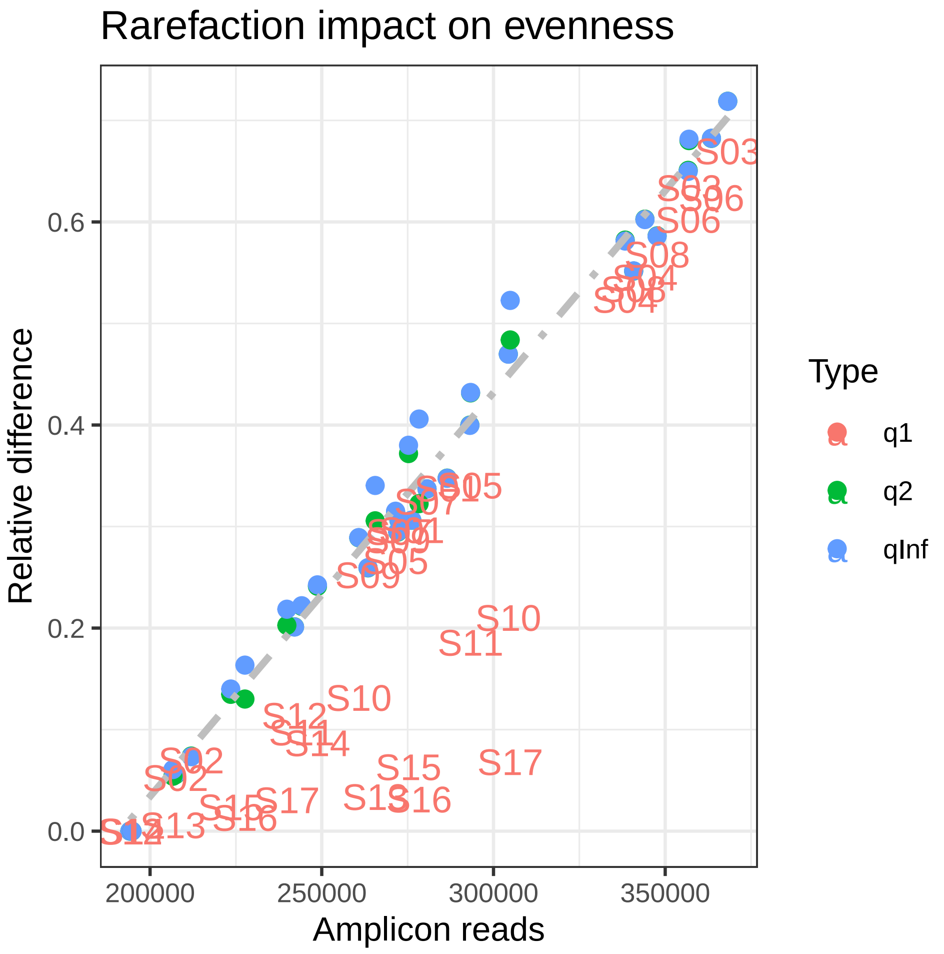

Figure S15: Impact of rarefaction of each sample to the reference size of 194,000 reads, on evenness values: scatter-plot of raw reads per amplicon to the relative difference: ((rare-raw)/raw.
q1: ${}^{1}E = {}^{1}D/{}^{0}D$; q2: ${}^{2}E = {}^{2}D/{}^{0}D$; qInf: ${}^{\infty}E = {}^{\infty}D/{}^{0}D$.
Labels are represented for q1 values.
Dash-dot line: linear regression of the ensemble of evenness values corresponding to q2 and qInf (${}^{2}E$ and ${}^{\infty}E$).
