# Supplementary material for: In-Host Flat-like Quasispecies: Characterization Methods and Clinical Implications
Source: Microorganisms. 2024 May 17;12(5):1011. doi: 10.3390/microorganisms12051011 (PMC11124460; doi:10.3390/microorganisms12051011)
Supplement: Supplementary file 1 [file microorganisms-12-01011-s001.zip › Supplementary-File S1/SupplFig11_NewID.NoRplc_SubsamplingEvenness.pdf]

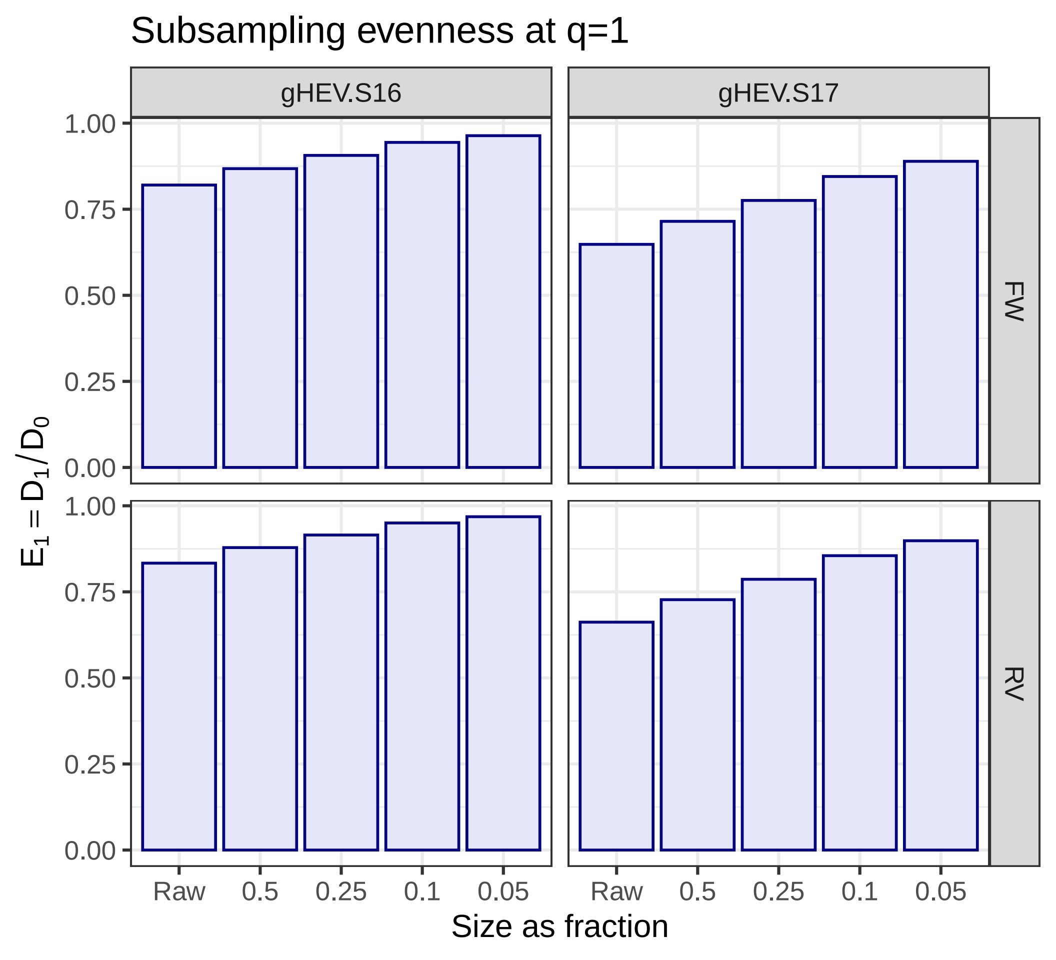
Subsampling evenness at q=1
gHEV.S16
gHEV.S17
FW
RV
1.00
0.75
0.50
0.25
0.00
$E_1 = D_1/D_0$
Raw
0.5
0.25
0.1
0.05
Size as fraction

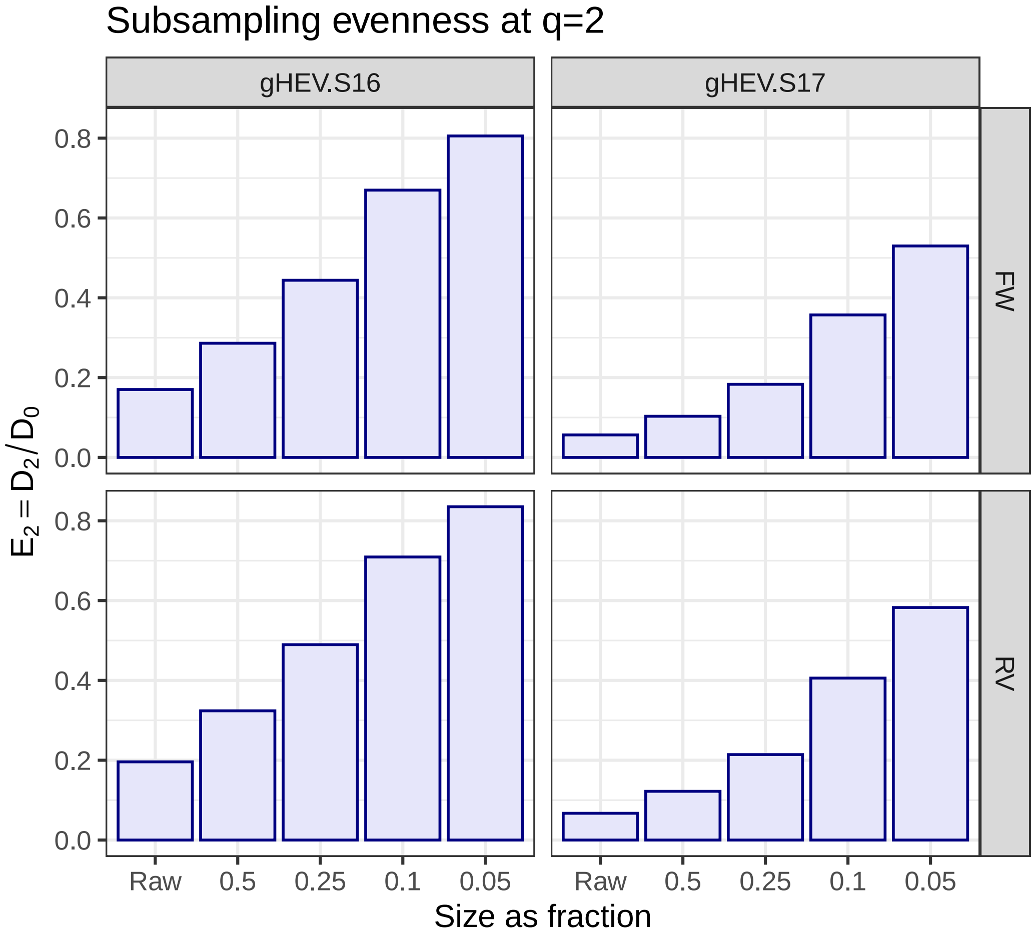

Subsampling evenness at q=2
gHEV.S16
gHEV.S17
FW
RV
0.0
0.2
0.4
0.6
0.8
$E_2 = D_2/D_0$
Raw
0.5
0.25
0.1
0.05
Size as fraction

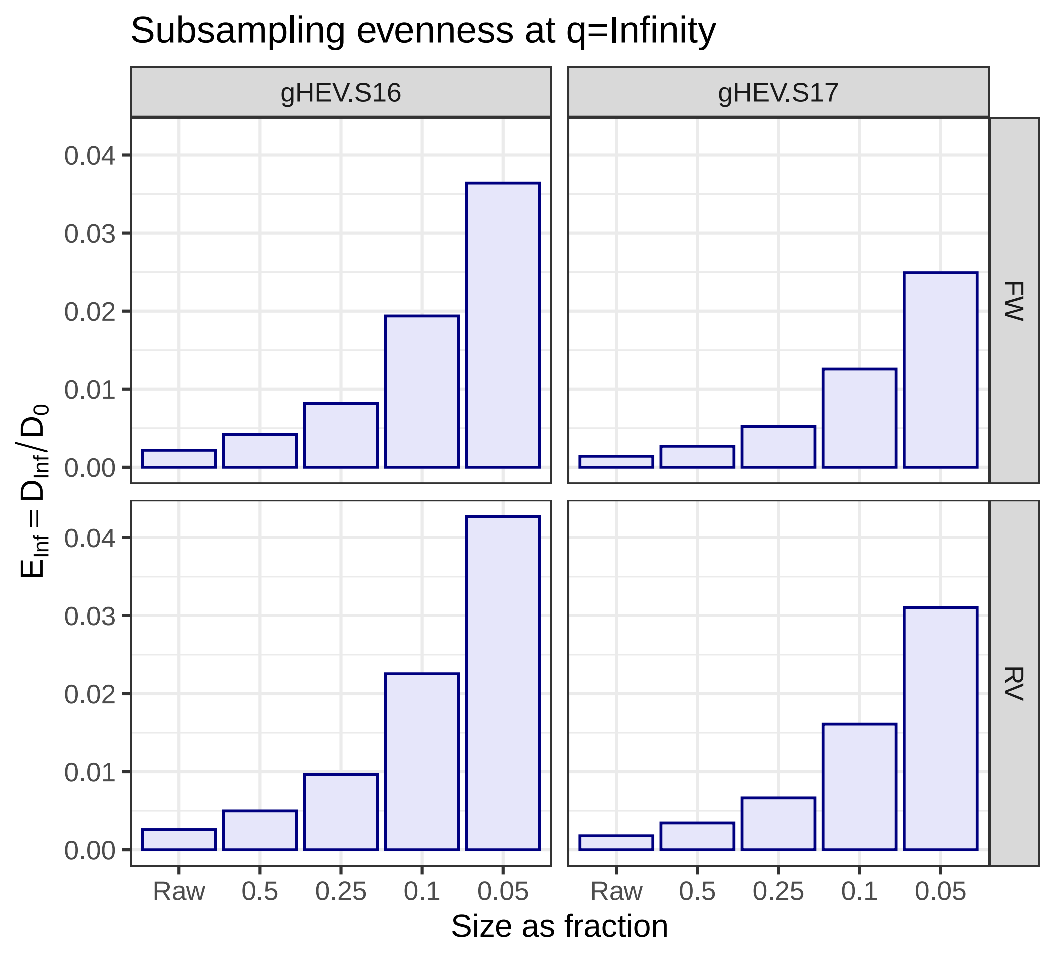

Subsampling evenness at q=Infinity
gHEV.S16
gHEV.S17
FW
RV
0.04
0.03
0.02
0.01
0.00
$E_{Inf} = D_{Inf}/D_0$
Raw
0.5
0.25
0.1
0.05
Size as fraction

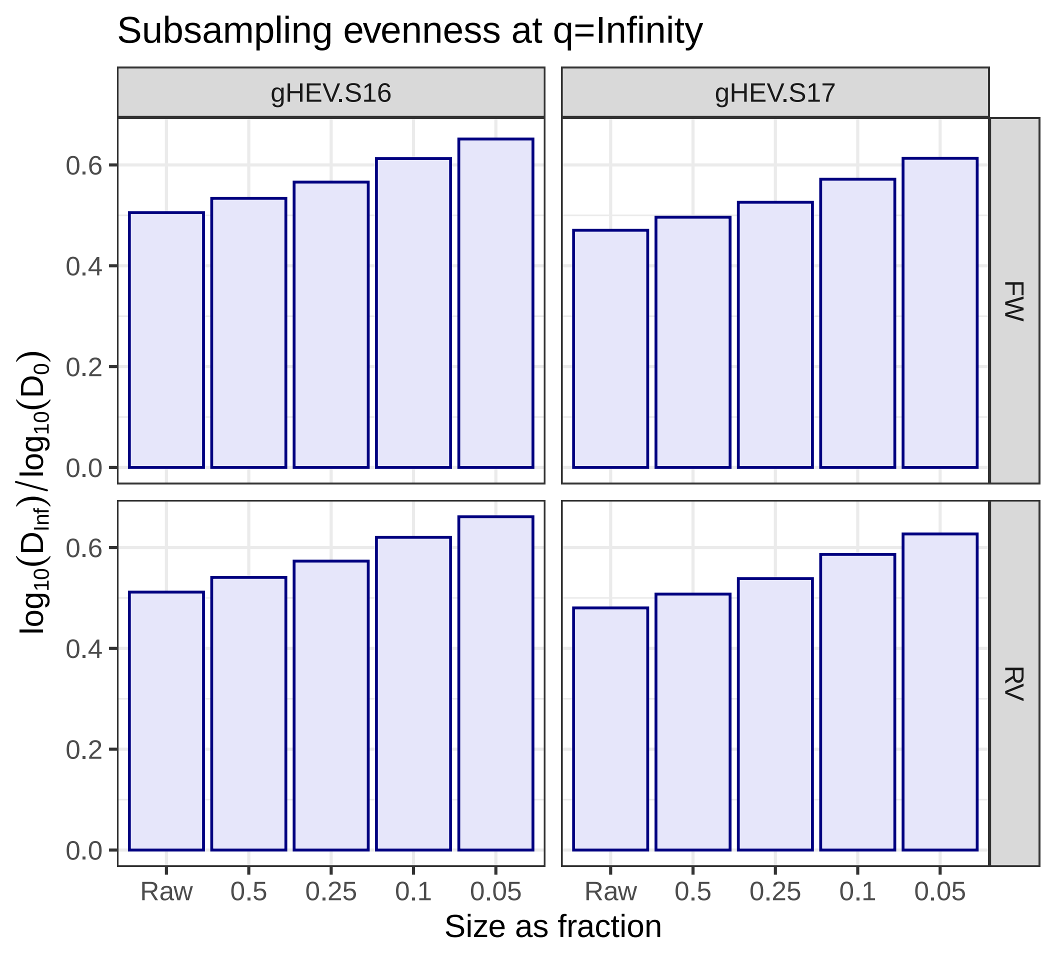

Subsampling evenness at q=Infinity
gHEV.S16
gHEV.S17
FW
RV
0.0
0.2
0.4
0.6
$\log_{10}(D_{Inf})/\log_{10}(D_0)$
Raw
0.5
0.25
0.1
0.05
Size as fraction
