# Supplementary material for: In-Host Flat-like Quasispecies: Characterization Methods and Clinical Implications
Source: Microorganisms. 2024 May 17;12(5):1011. doi: 10.3390/microorganisms12051011 (PMC11124460; doi:10.3390/microorganisms12051011)
Supplement: Supplementary file 1 [file microorganisms-12-01011-s001.zip › Supplementary-File S1/SupplFig14_FW&RV_Rarefied_RelativeLogarithmicEvennessProfile.pdf]

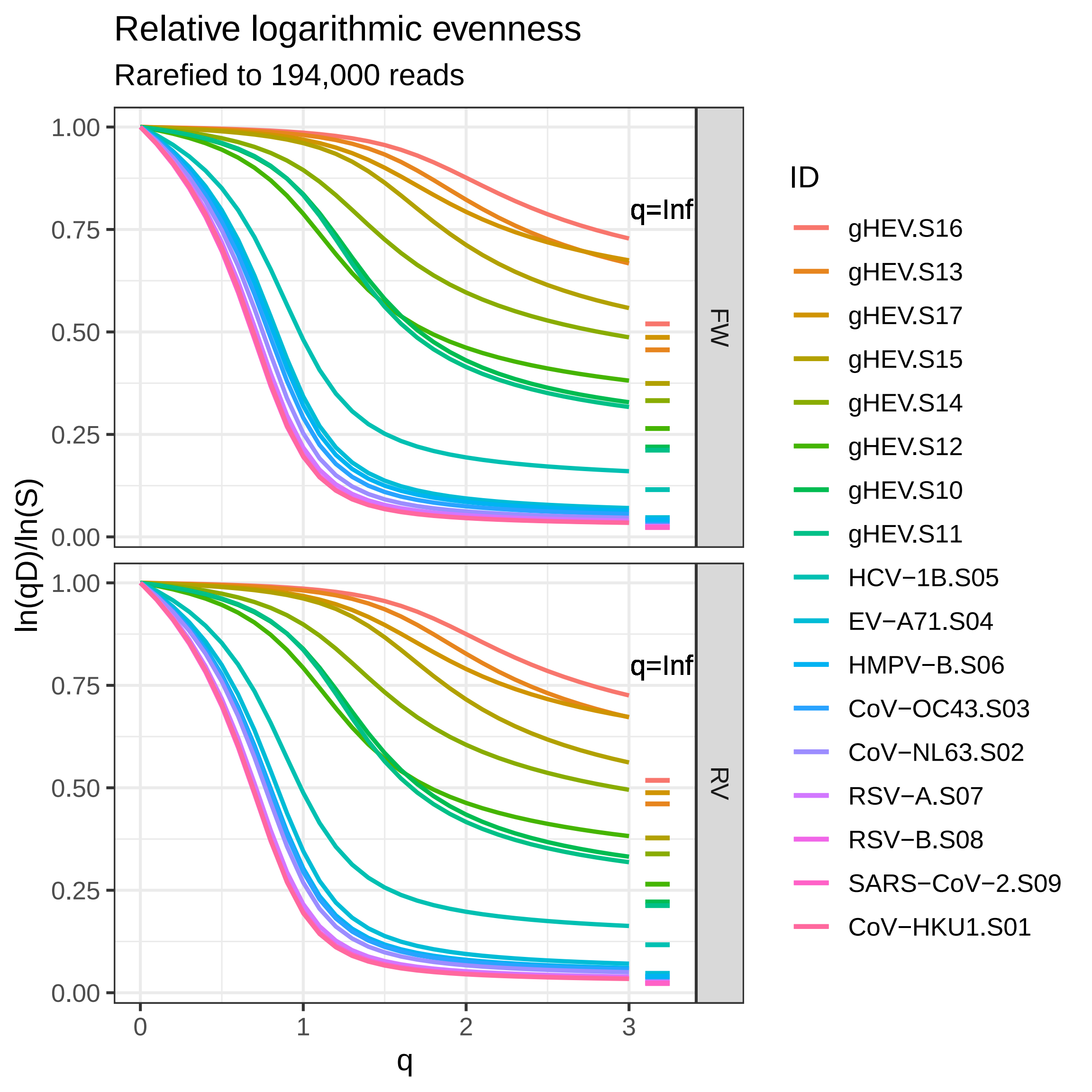

Relative logarithmic evenness
Rarefied to 194,000 reads
ln(qD)/ln(S)
q
q=Inf
FW
RV
1.00
0.75
0.50
0.25
0.00
0
1
2
3
ID
gHEV.S16
gHEV.S13
gHEV.S17
gHEV.S15
gHEV.S14
gHEV.S12
gHEV.S10
gHEV.S11
HCV−1B.S05
EV−A71.S04
HMPV−B.S06
CoV−OC43.S03
CoV−NL63.S02
RSV−A.S07
RSV−B.S08
SARS−CoV−2.S09
CoV−HKU1.S01
