# Supplementary material for: In-Host Flat-like Quasispecies: Characterization Methods and Clinical Implications
Source: Microorganisms. 2024 May 17;12(5):1011. doi: 10.3390/microorganisms12051011 (PMC11124460; doi:10.3390/microorganisms12051011)
Supplement: Supplementary file 1 [file microorganisms-12-01011-s001.zip › Supplementary-File S2/FlatQuasispecies_Supplementary-File S2.pdf]

In-host flat-like quasispecies, methods and clinical implications

Supplementary material - Near-flat quasispecies examples

Josep Gregori[1], Sergi Colomer-Castell[1,2,3], Marta Ibañez Lligoña [1,2,4], Damir Garcia-Cehic[1,2], Carolina Campos[1,2,3], Maria Buti[1,2,4], Mar Riveiro-Barciela[1,2,4], Cristina Andrés[5,6], Maria Piñana[5,6], Alejandra Gonzalez-Sánchez[5,6], Francisco Rodriguez-Frias[2,7], Maria Francesca Cortese[2,6,7], David Tabernero[1,2,7], Tomás Pumarola[3,5,6,7], Juan Ignacio Esteban[1,2,4], Andrés Antón[5,6], and Josep Quer[1,2,3,4]

[1] Liver Diseases-Viral Hepatitis, Liver Unit, Vall d'Hebron Institut de Recerca (VHIR), Vall d'Hebron Hospital Universitari, Vall d'Hebron Barcelona Hospital Campus, Passeig Vall d'Hebron 119-129, 08035 Barcelona, Spain

[2] Centro de Investigación Biomédica en Red de Enfermedades Hepáticas y Digestivas (CIBERehd), Instituto de Salud Carlos III, Av. Monforte de Lemos, 3-5, 28029 Madrid, Spain

[3] Biochemistry and Molecular Biology Department, UAB, Bellaterra, Spain

[4] Medicine Department, Universitat Autònoma de Barcelona (UAB), Campus de la UAB, Plaça Cívica, 08193 Bellaterra, Spain

[5] Centro de Investigación Biomédica en Red de Enfermedades Infecciosas (CIBERINFEC), Instituto de Salud Carlos III, Av. Monforte de Lemos, 3-5, 28029 Madrid, Spain

[6] Microbiology Department, Vall d'Hebron Institut de Recerca (VHIR), Vall d'Hebron Hospital Universitari, Vall d'Hebron Barcelona Hospital Campus, Passeig Vall d'Hebron 119-129, 08035 Barcelona, Spain

[7] Biochemistry Department, Vall d'Hebron Institut de Recerca (VHIR), Vall d'Hebron Hospital Universitari, Vall d'Hebron Barcelona Hospital Campus, Passeig Vall d'Hebron 119-129, 08035 Barcelona, Spain

Corresponding authors:
Josep Gregori (josep.gregori@gmail.com) and Josep Quer (josep.quer@vhir.org)

Near-flat quasispecies - HCV treatment failures

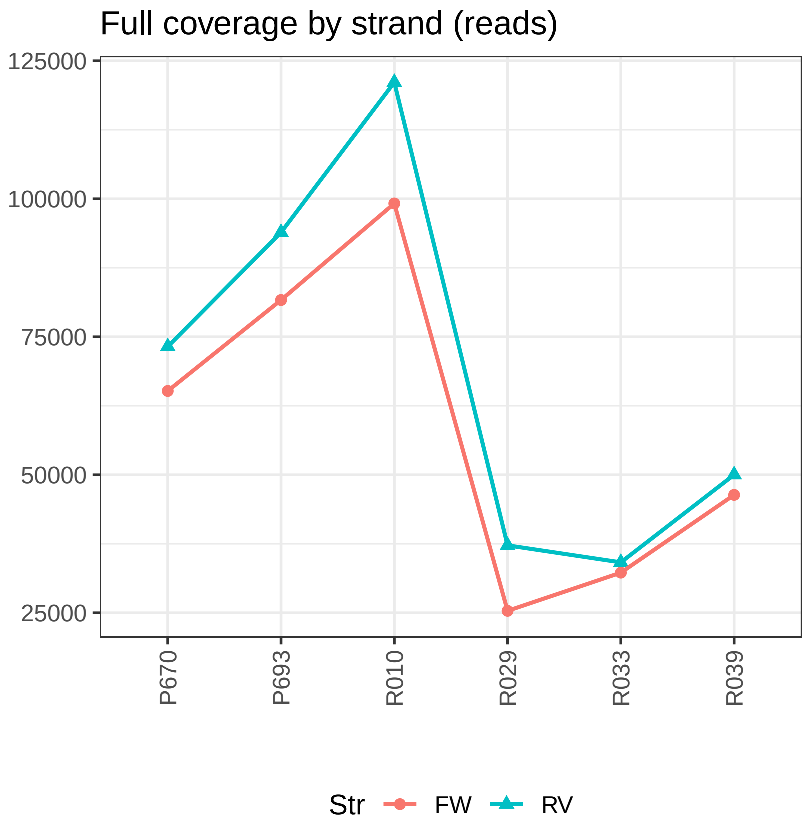

Figure NF-1: Coverage by amplicon and strand in read number.

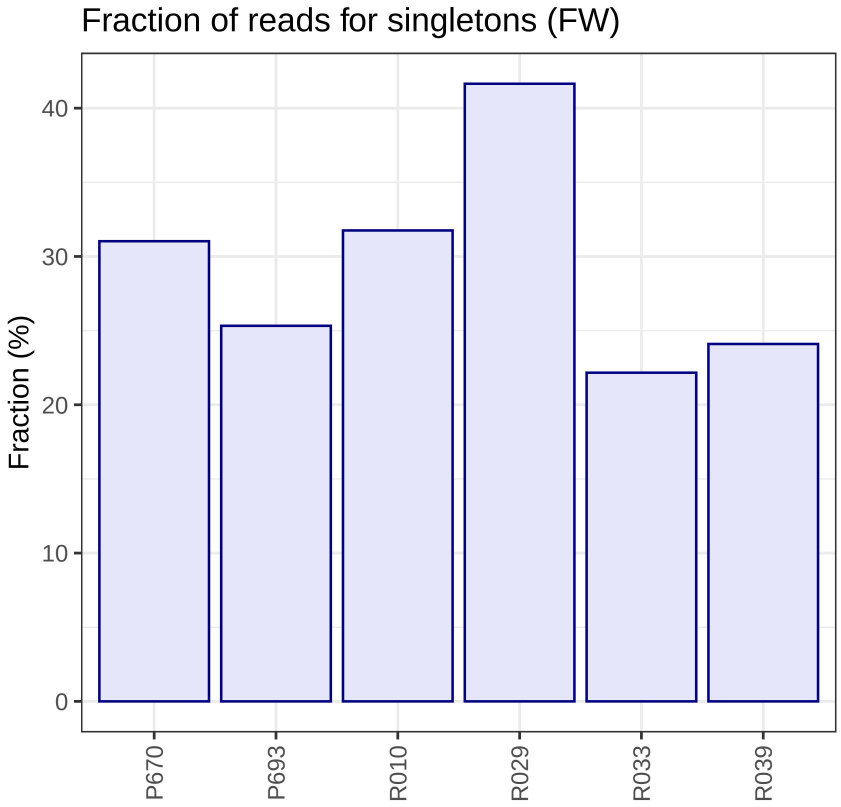

Figure NF-2: Fraction of reads for singletons observed with forward strand reads.

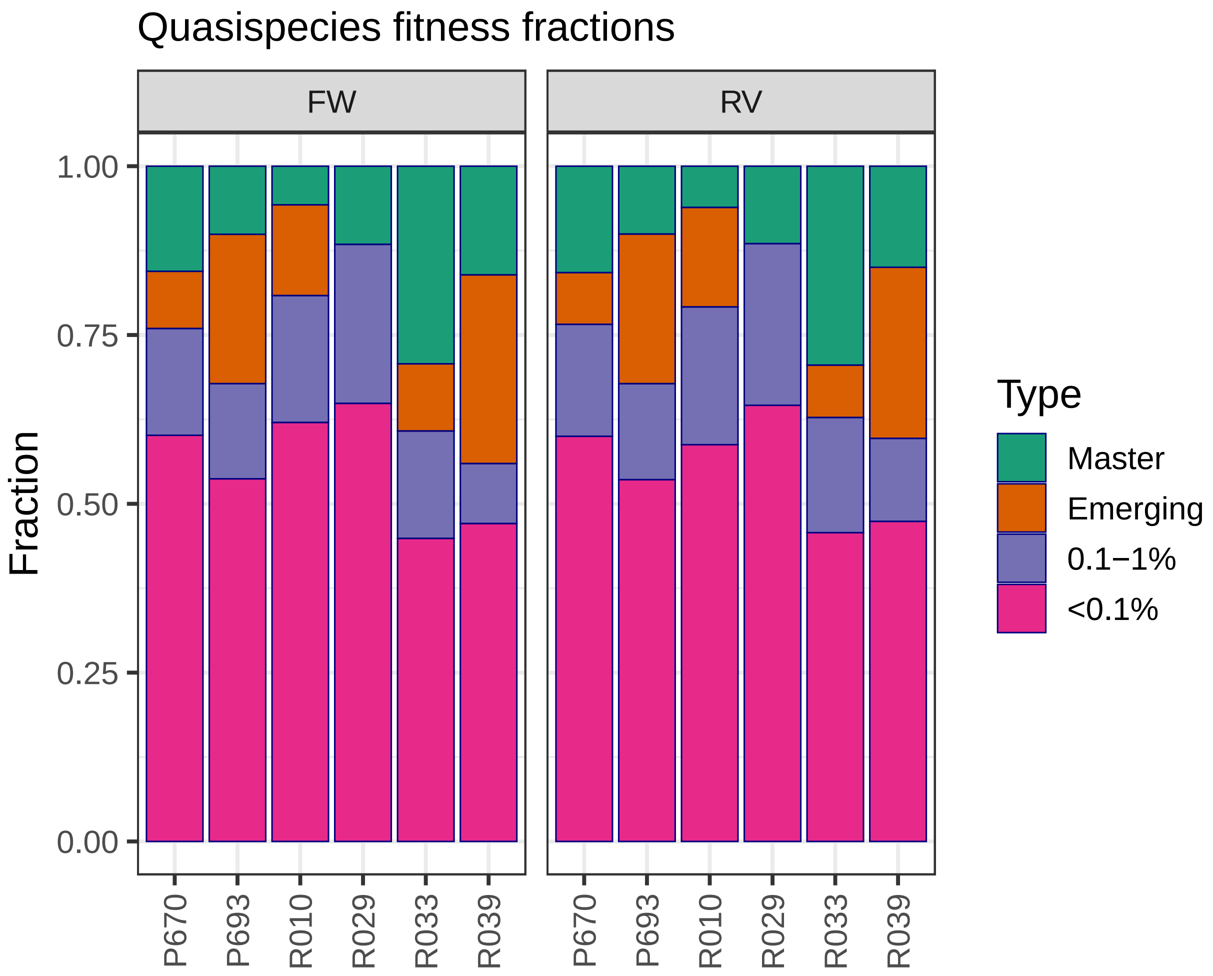

Figure NF-3: Quasispecies fitness fractions.

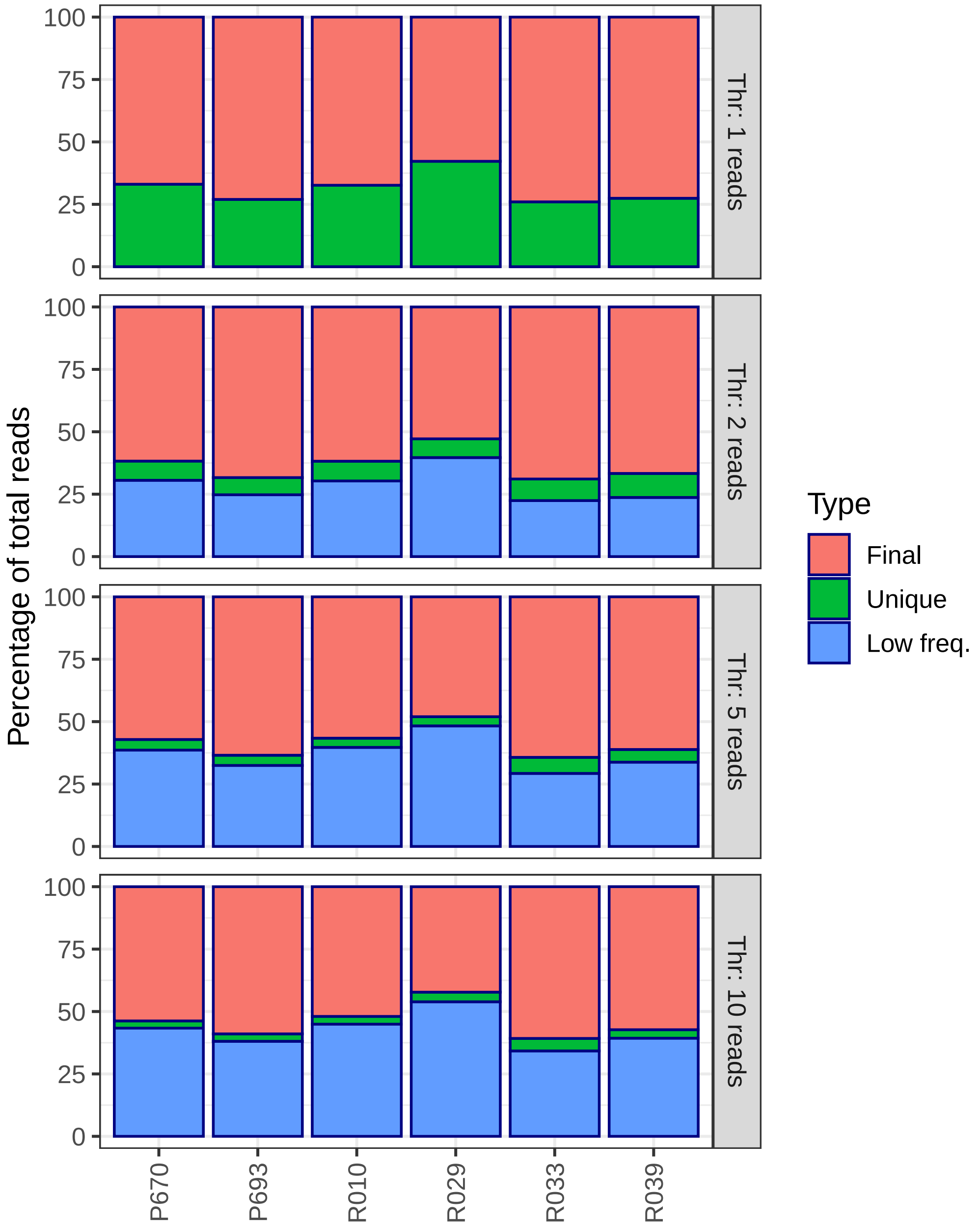

Figure NF-4: Impact of abundance filter followed by haplotype strand intersection. Abundance thresholds at 1, 2, 5 and 10 reads.

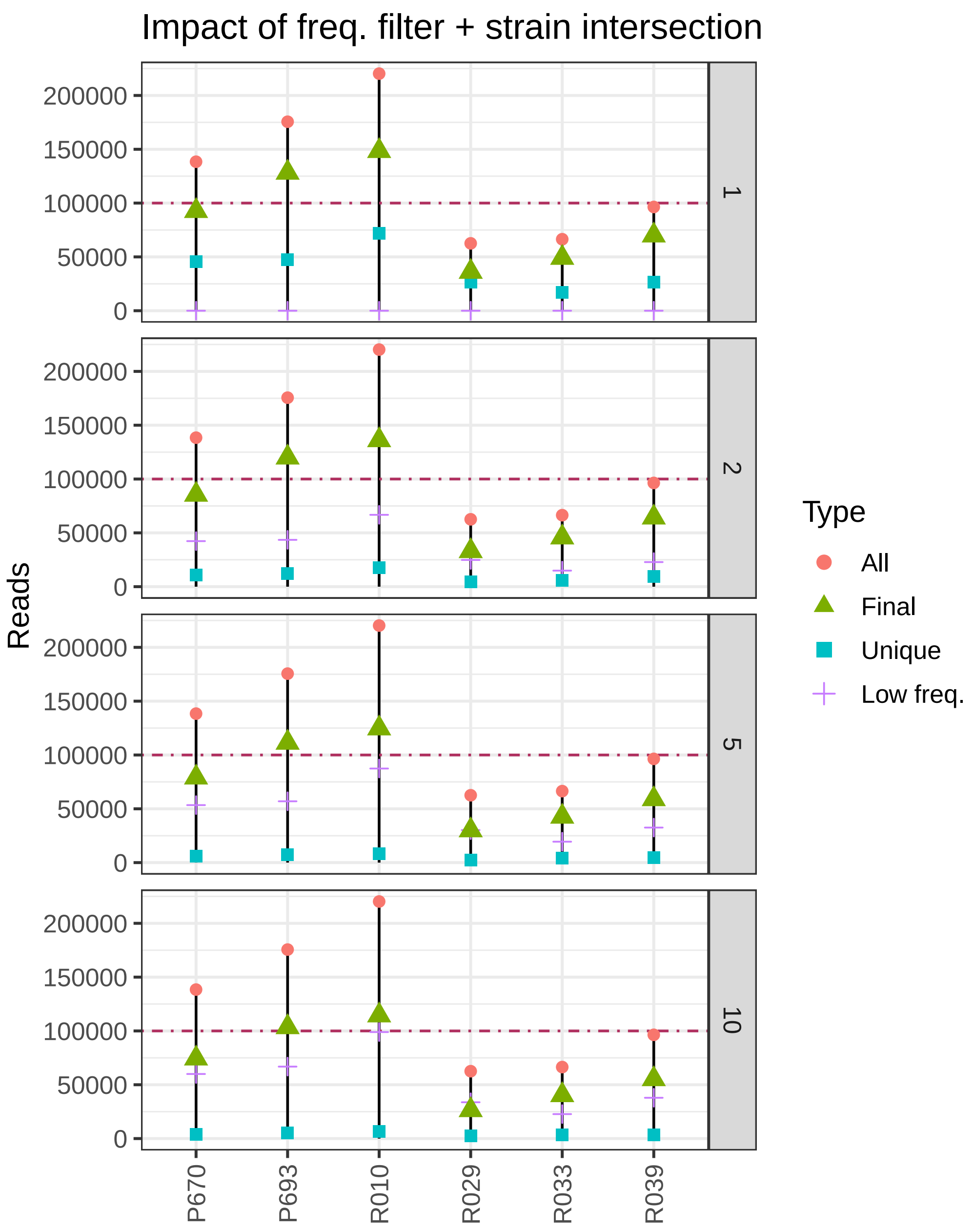

Figure NF-5: Impact of abundance filter followed by haplotype strand intersection. Abundance thresholds at 1, 2, 5 and 10 reads.

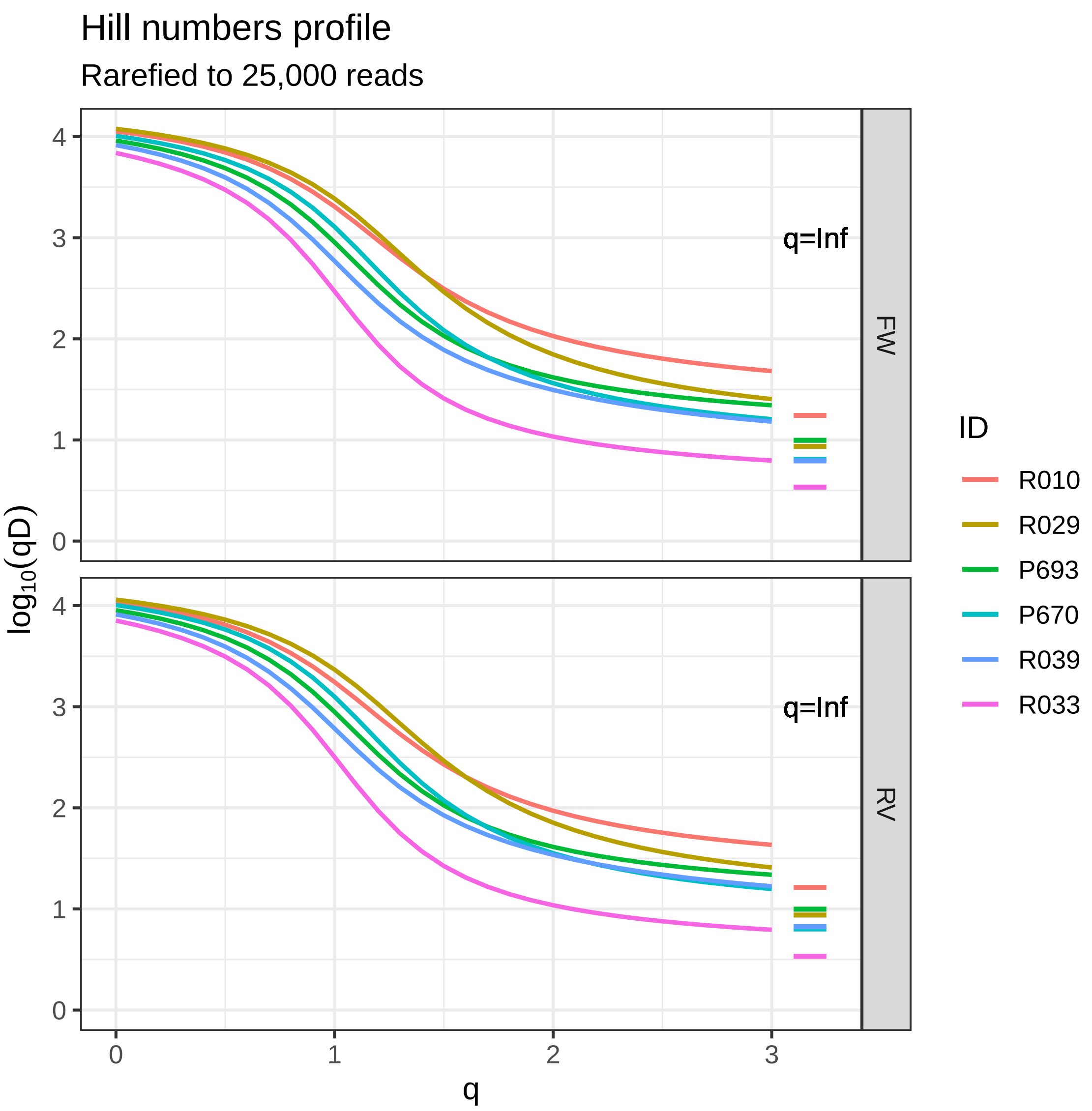

Figure NF-6: Hill numbers (HN) profiles rarefied to 25,000 reads.

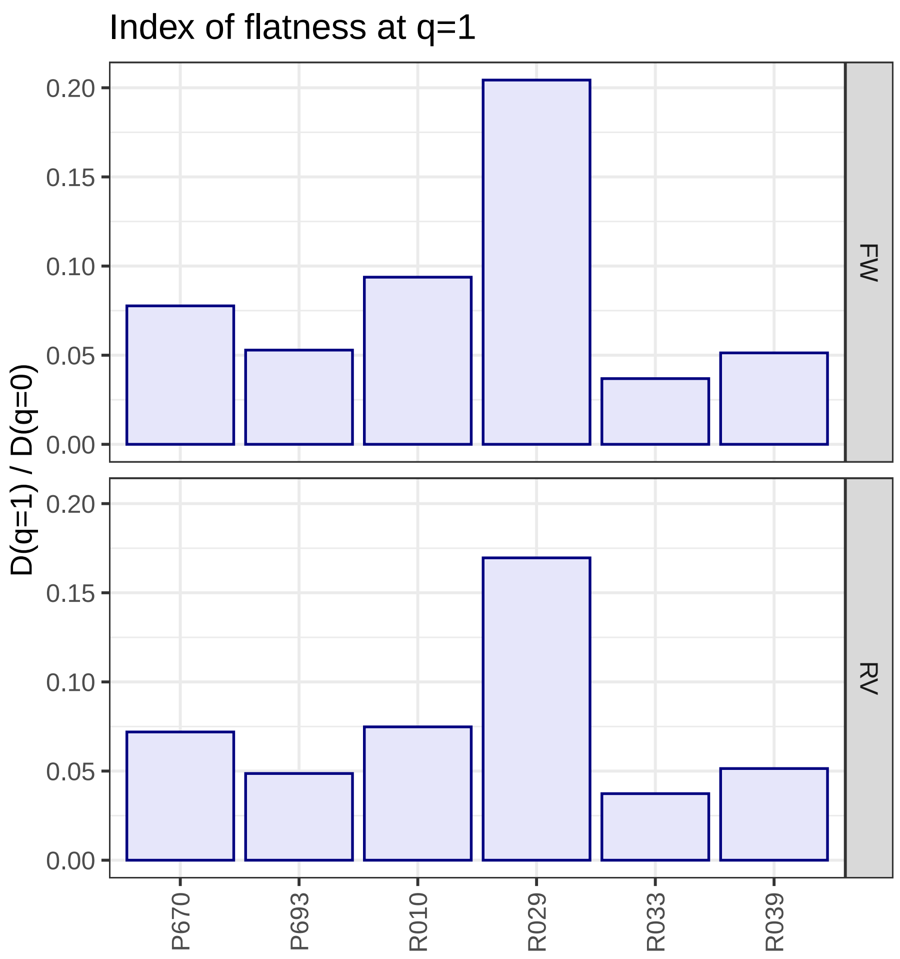

Figure NF-7: Evenness index ${}^1E = {}^1D/{}^0D$.

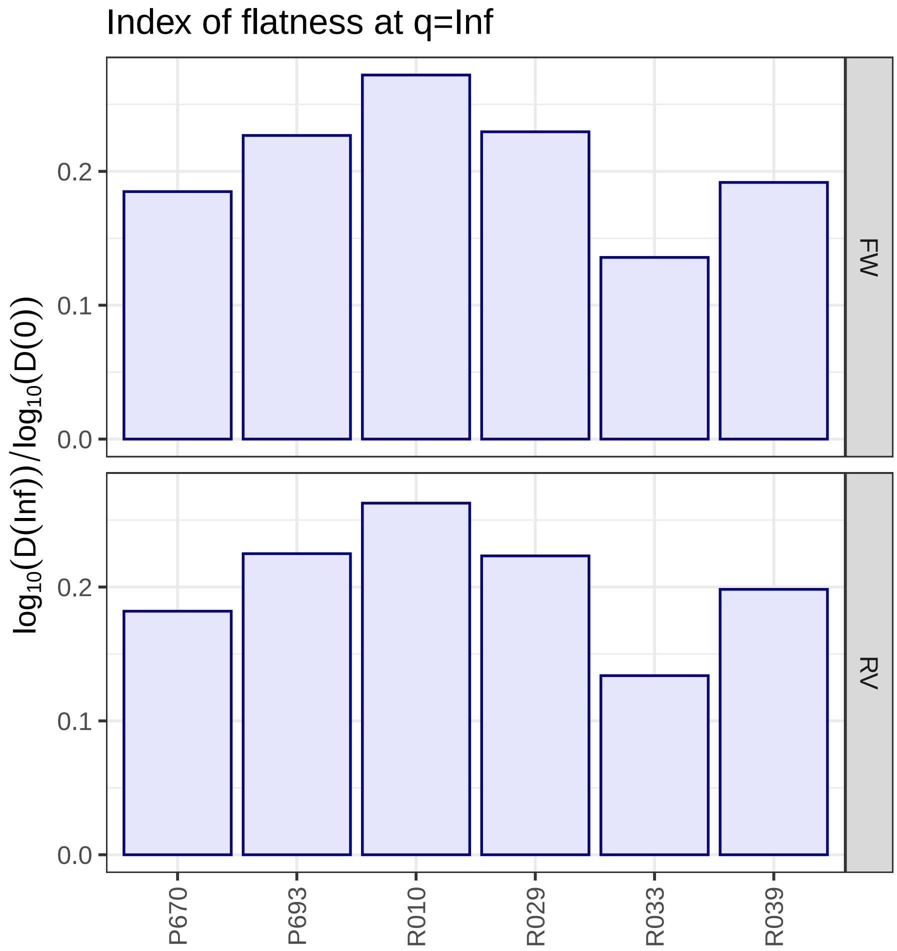

Figure NF-8: Evenness index eInf, $log_{10}(^{\infty}D) \,/\, log_{10}(^{0}D)$.

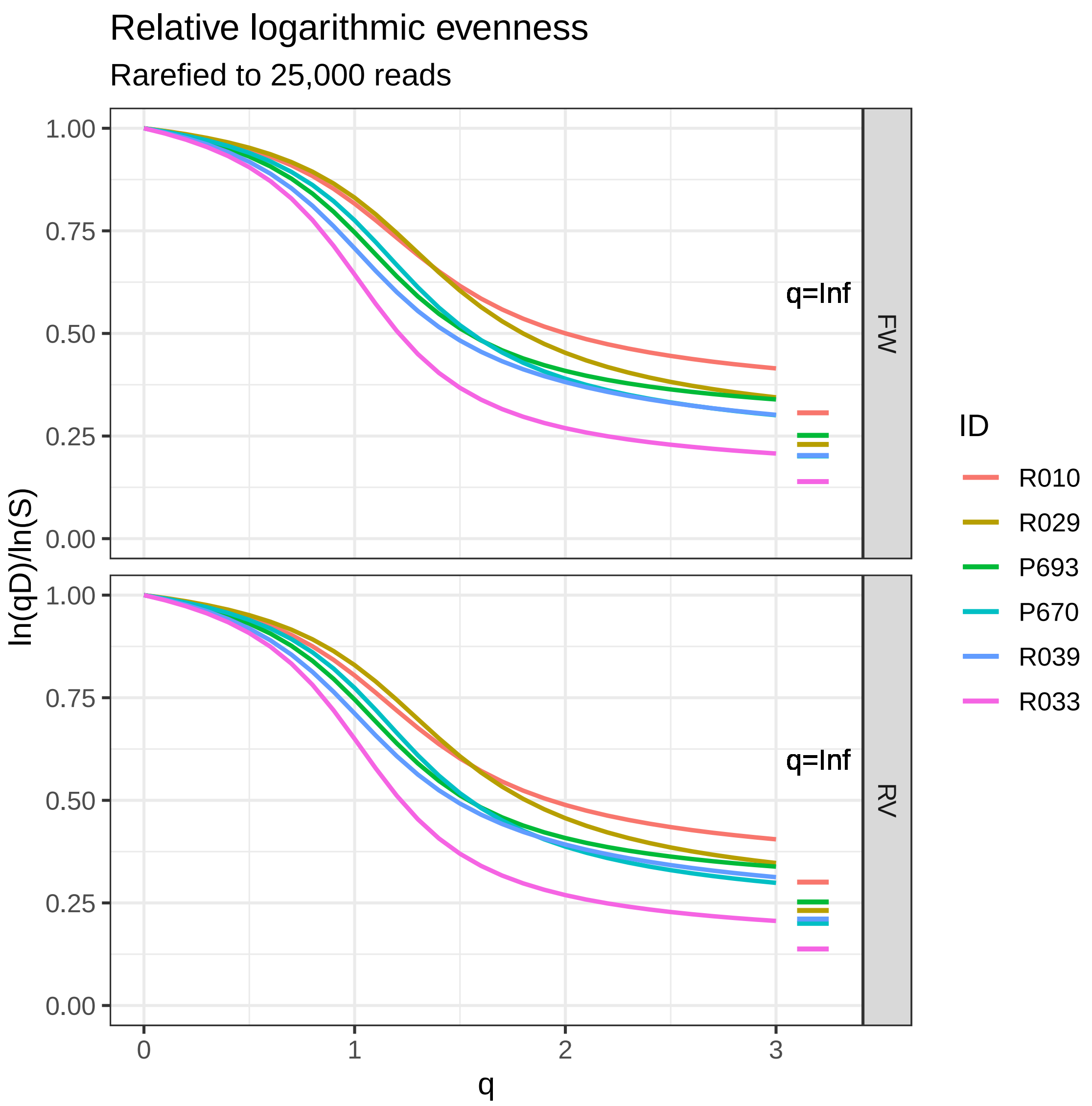

Figure NF-9: Relative logarithmic evenness (RLE) profiles rarefied to 25,000 reads.

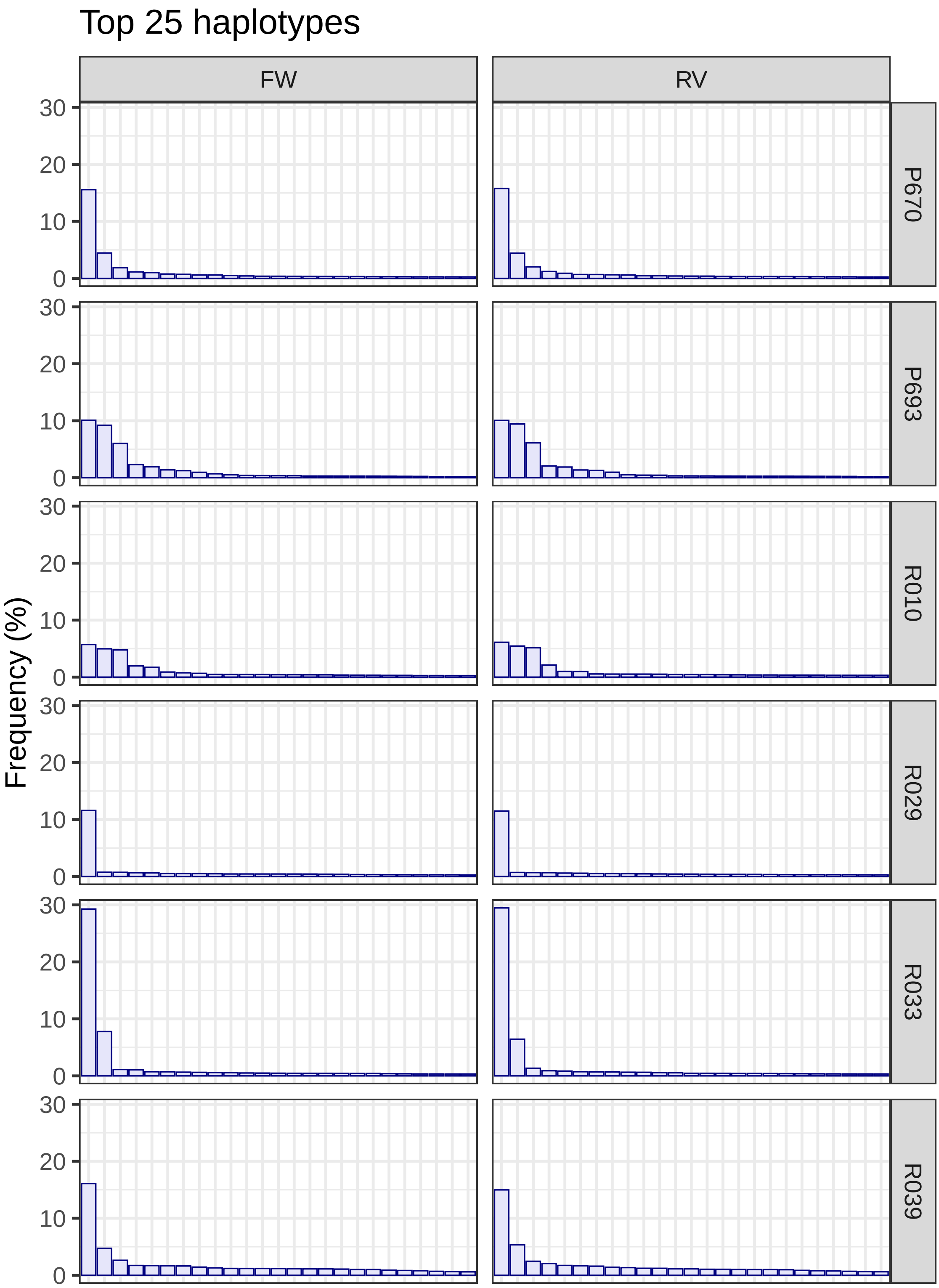

Figure NF-10: Frequencies of top 25 haplotypes.

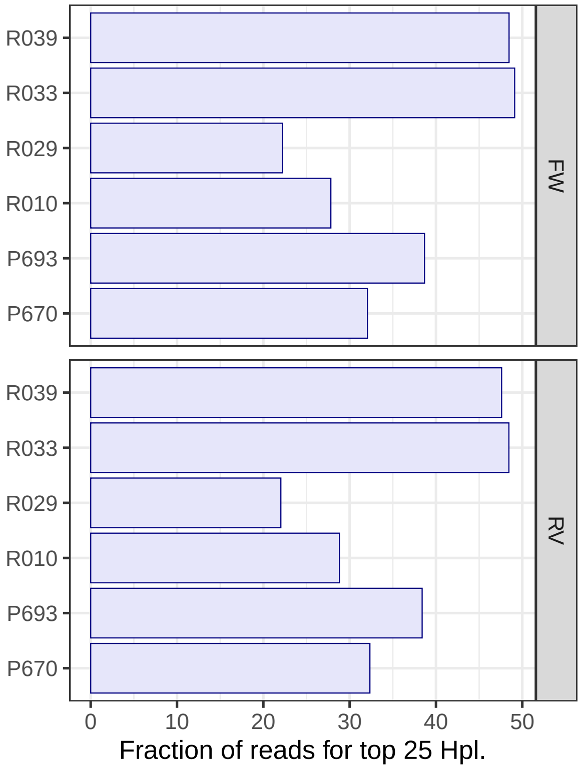

Figure NF-11: Fraction of reads for 25 top haplotypes.

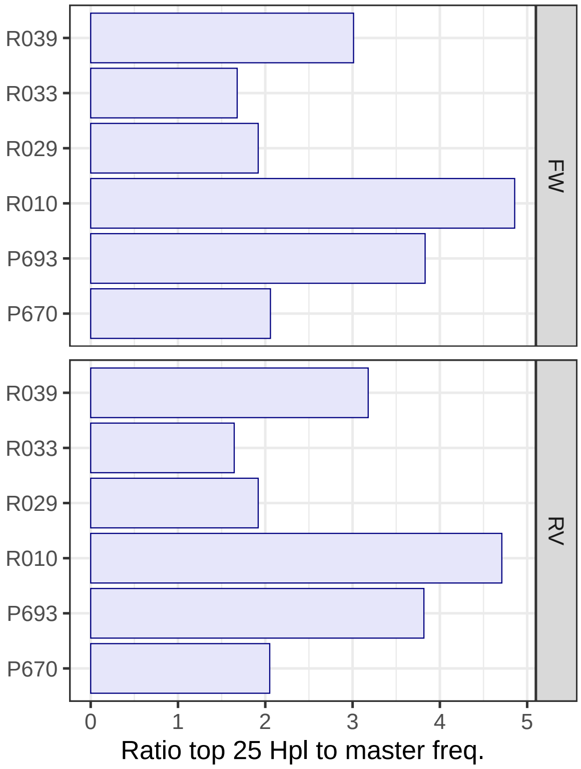

Figure NF-12: Ratio of reads for 25 top haplotypes to master haplotype.

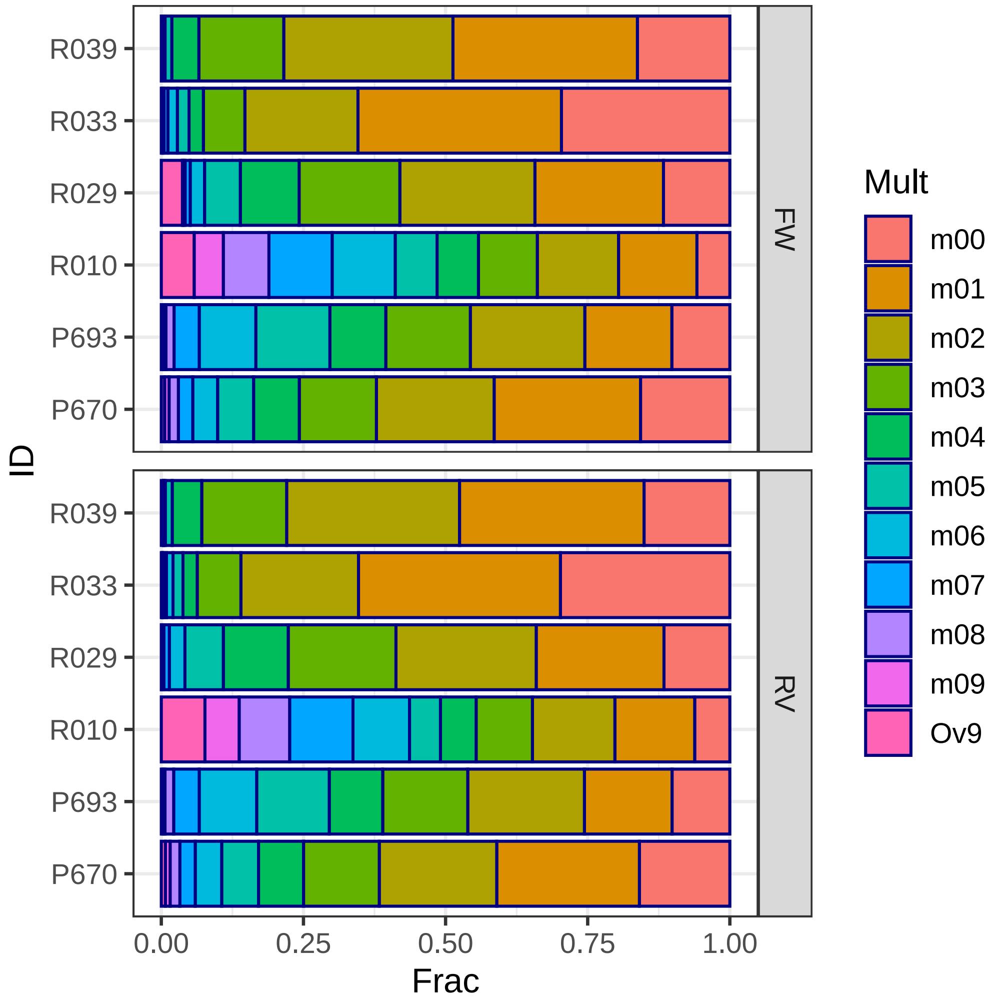

Figure NF-13: Fractions of reads with increasing number of substitutions with respect to the master haplotype in each sample. m00: fraction of reads for the master haplotype, m0x: fraction of reads for haplotypes showing x substitutions, Ov0: fraction of reads with over 9 substitutions.

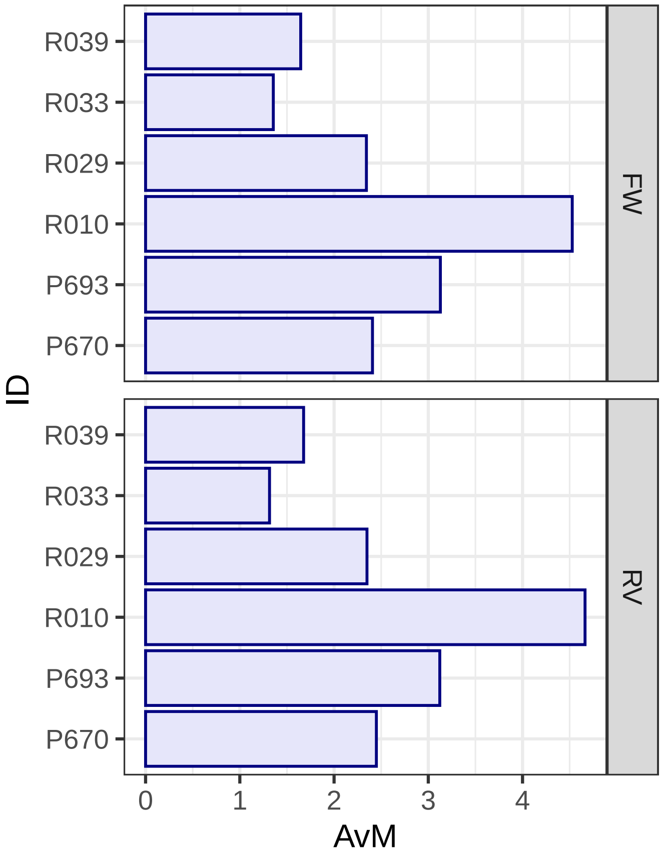

Figure NF-14: Mean number of substitutions per read with respect to the master haplotype in each amplicon and strand.
