# Supplementary figures and images for: In-Host Flat-like Quasispecies: Characterization Methods and Clinical Implications
Source: Microorganisms. 2024 May 17;12(5):1011. doi: 10.3390/microorganisms12051011 (PMC11124460; doi:10.3390/microorganisms12051011)

# Full coverage by strand (reads)

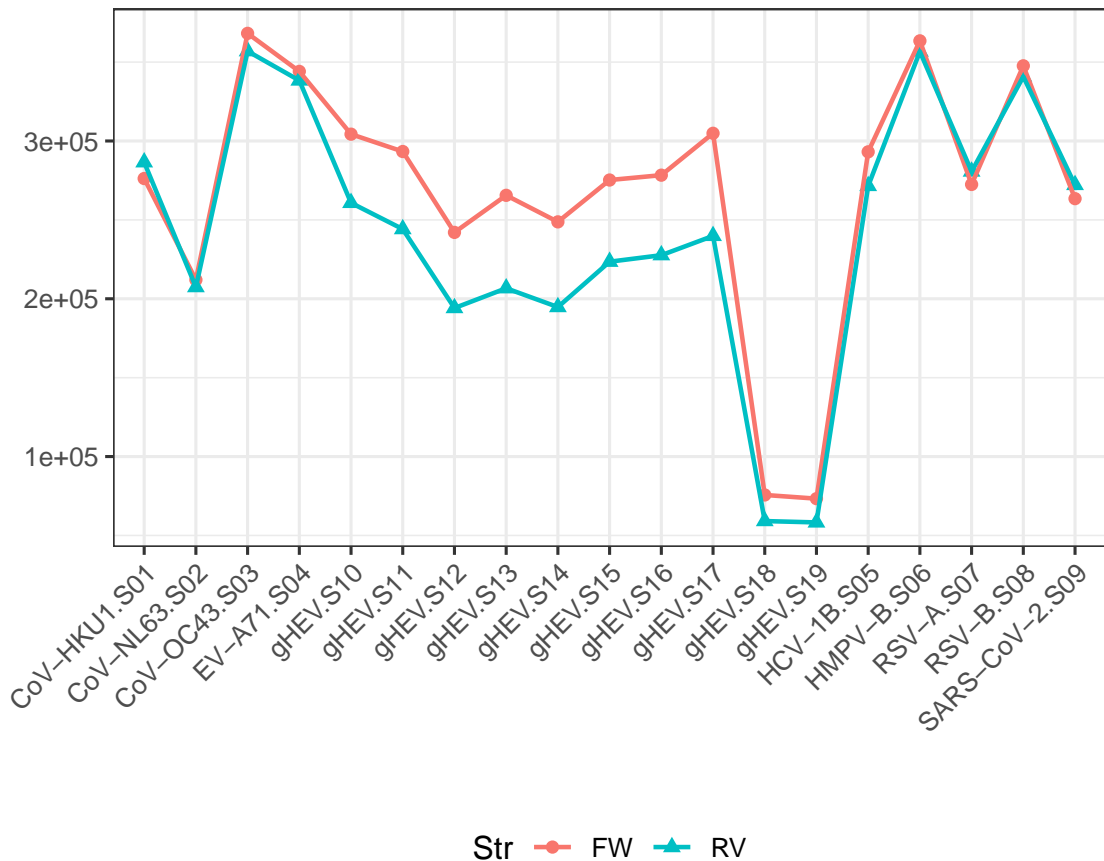

Supplement: Supplementary file 1 [file microorganisms-12-01011-s001.zip › Supplementary-File S1/SupplFig01_FullCoverageByStrand.pdf]

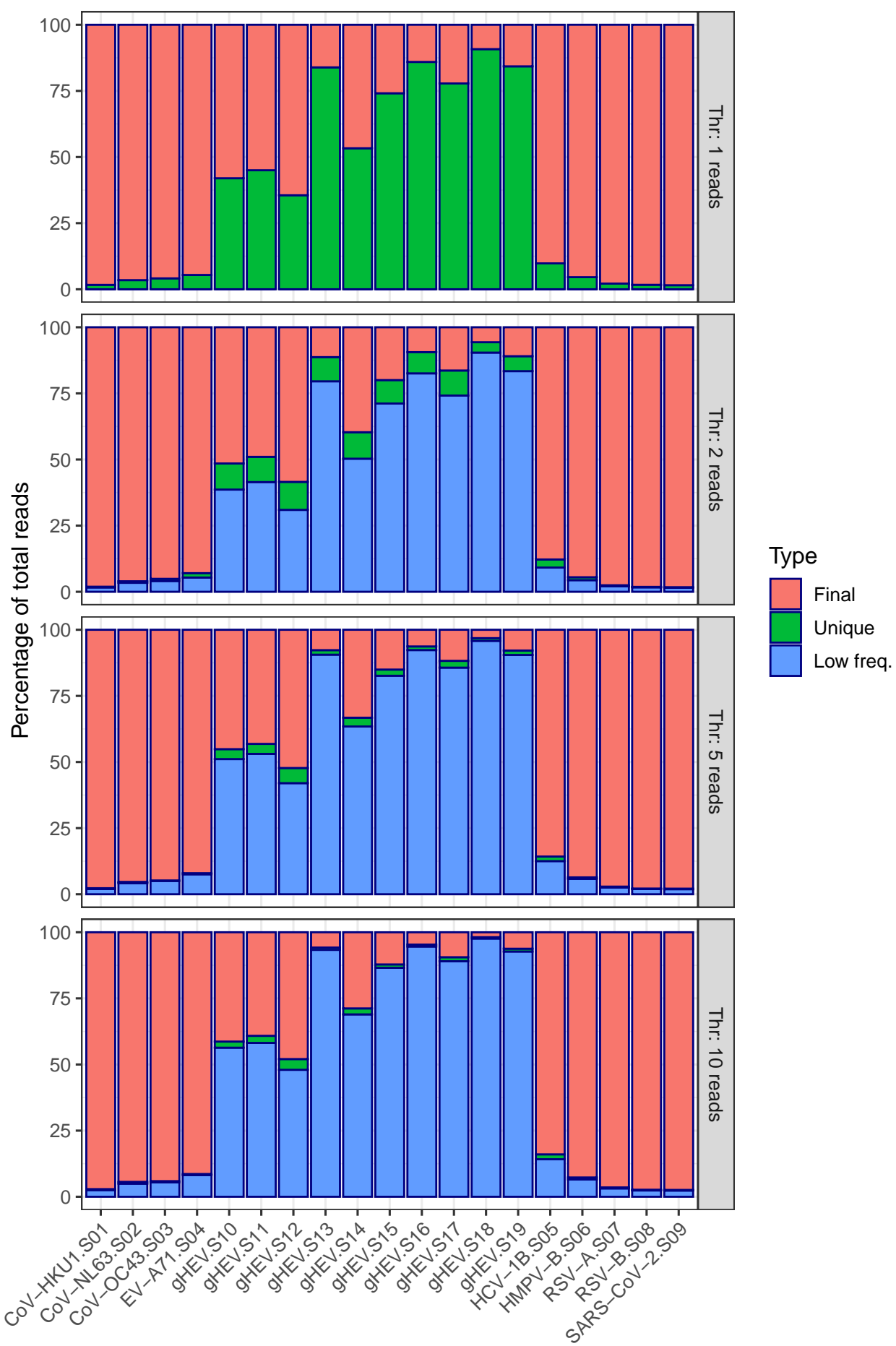

Supplement: Supplementary file 1 [file microorganisms-12-01011-s001.zip › Supplementary-File S1/SupplFig02_Intersects_ReadsImpact_AtThresholds.pdf]

# Hill number profile (raw values)

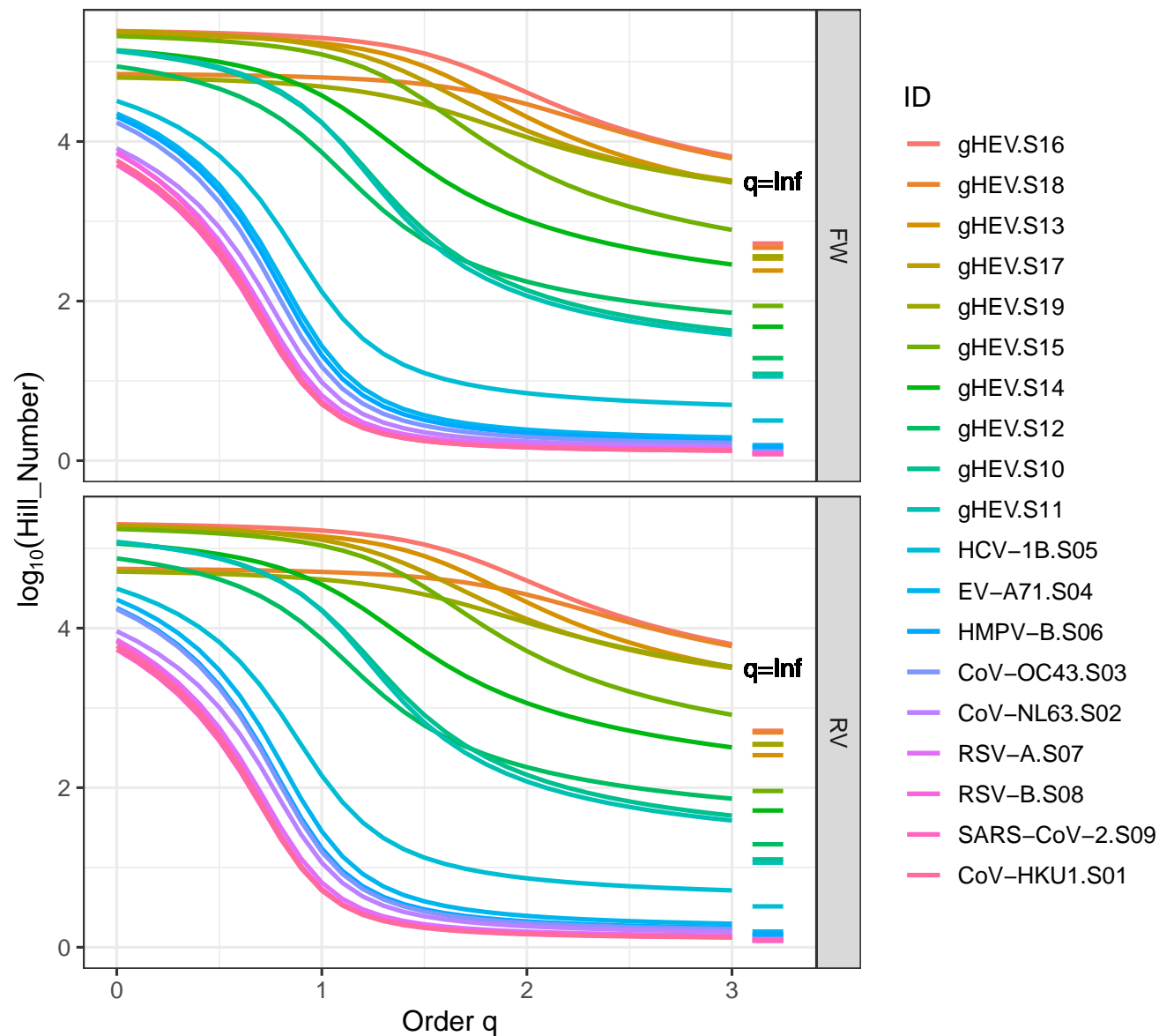

Supplement: Supplementary file 1 [file microorganisms-12-01011-s001.zip › Supplementary-File S1/SupplFig03_FW&RV_HillNumbersProfile_2.pdf]

# Hill evenness profile (raw values)

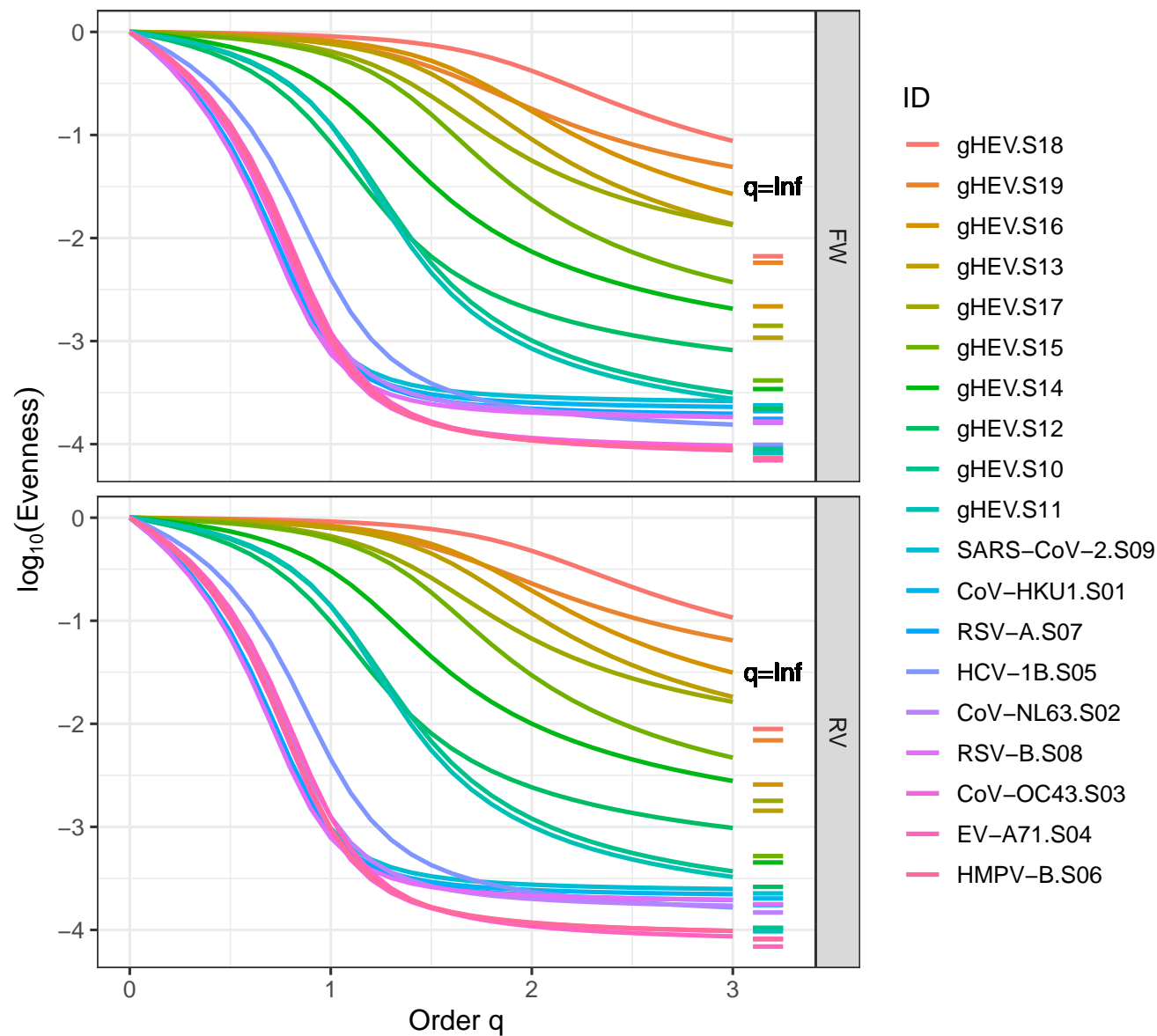

Supplement: Supplementary file 1 [file microorganisms-12-01011-s001.zip › Supplementary-File S1/SupplFig04_FW&RV_HillEvennessProfile.pdf]

# Index of flatness at $q=1$

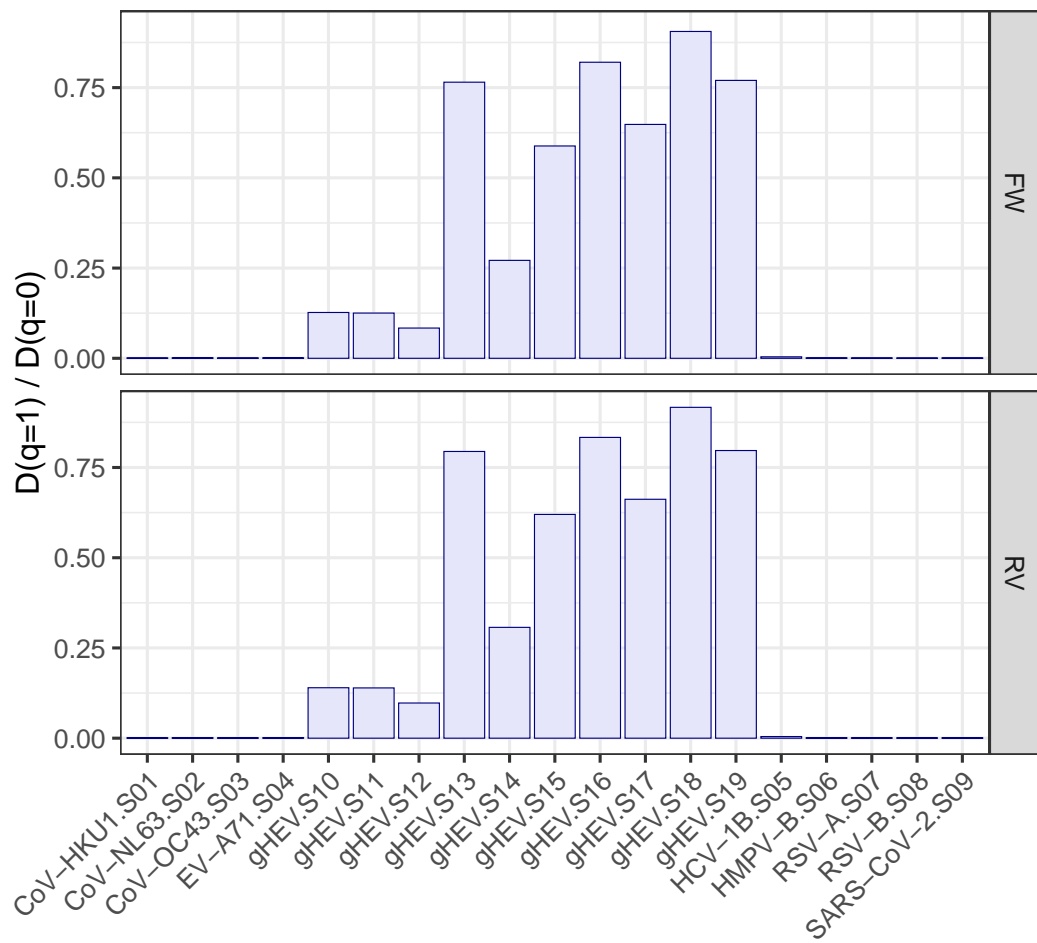

Supplement: Supplementary file 1 [file microorganisms-12-01011-s001.zip › Supplementary-File S1/SupplFig05_FW&RV_FlatnessIndex_q1.pdf]

# Index of flatness at q=Inf

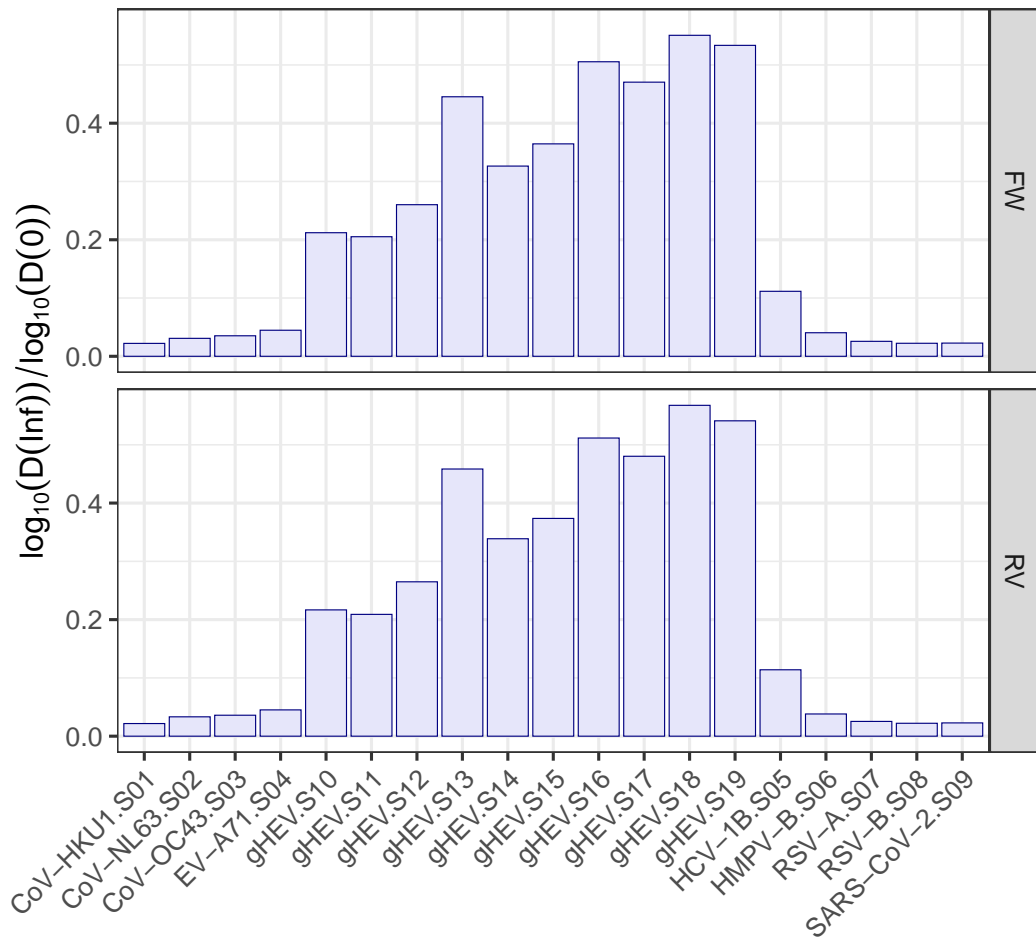

Supplement: Supplementary file 1 [file microorganisms-12-01011-s001.zip › Supplementary-File S1/SupplFig06_FW&RV_FlatnessIndex_Log.qInf.pdf]

## Top 25 haplotypes

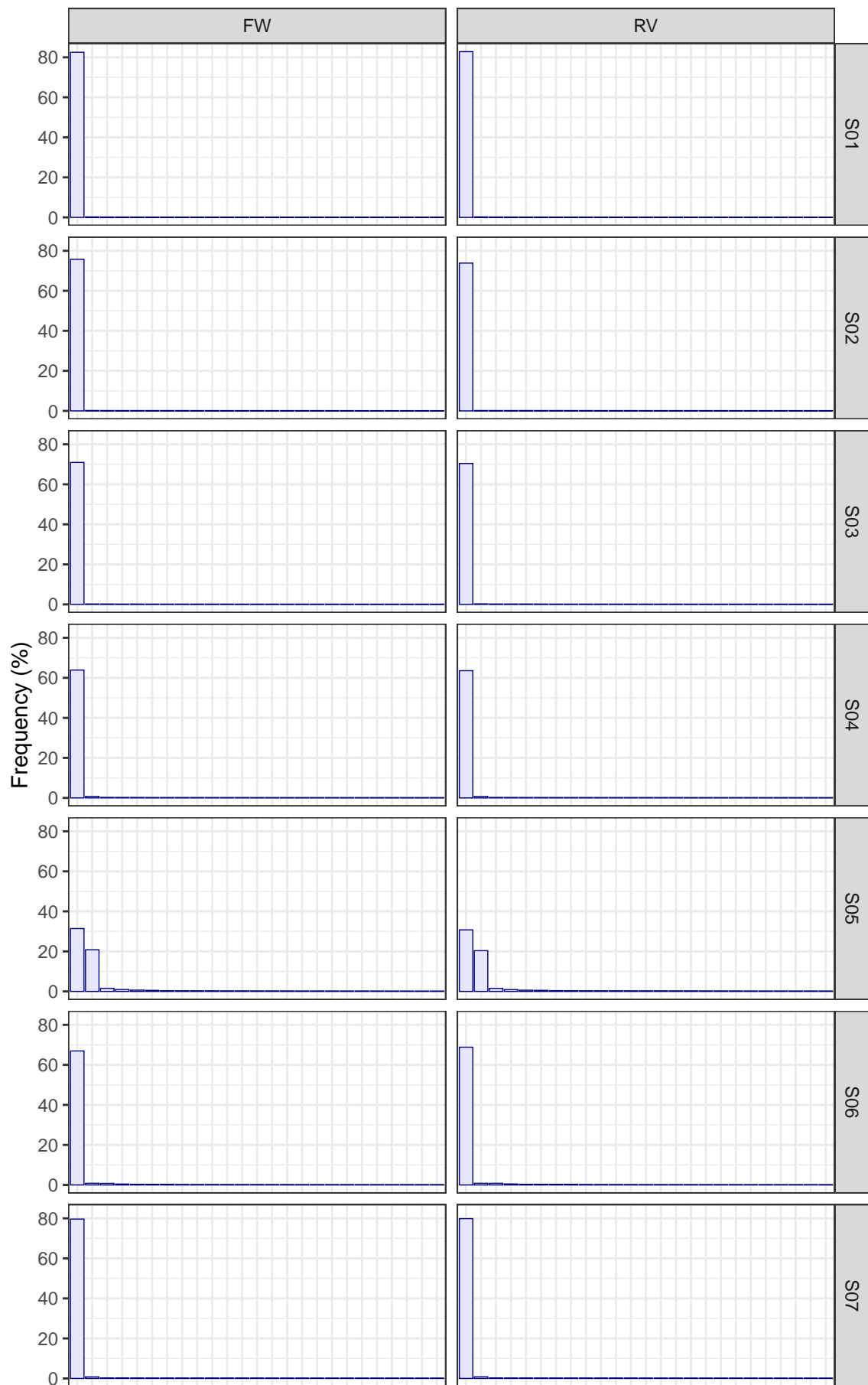

Supplement: Supplementary file 1 [file microorganisms-12-01011-s001.zip › Supplementary-File S1/SupplFig07_Top25_HaplotypeFreqs-1.pdf]

# Top 25 haplotypes

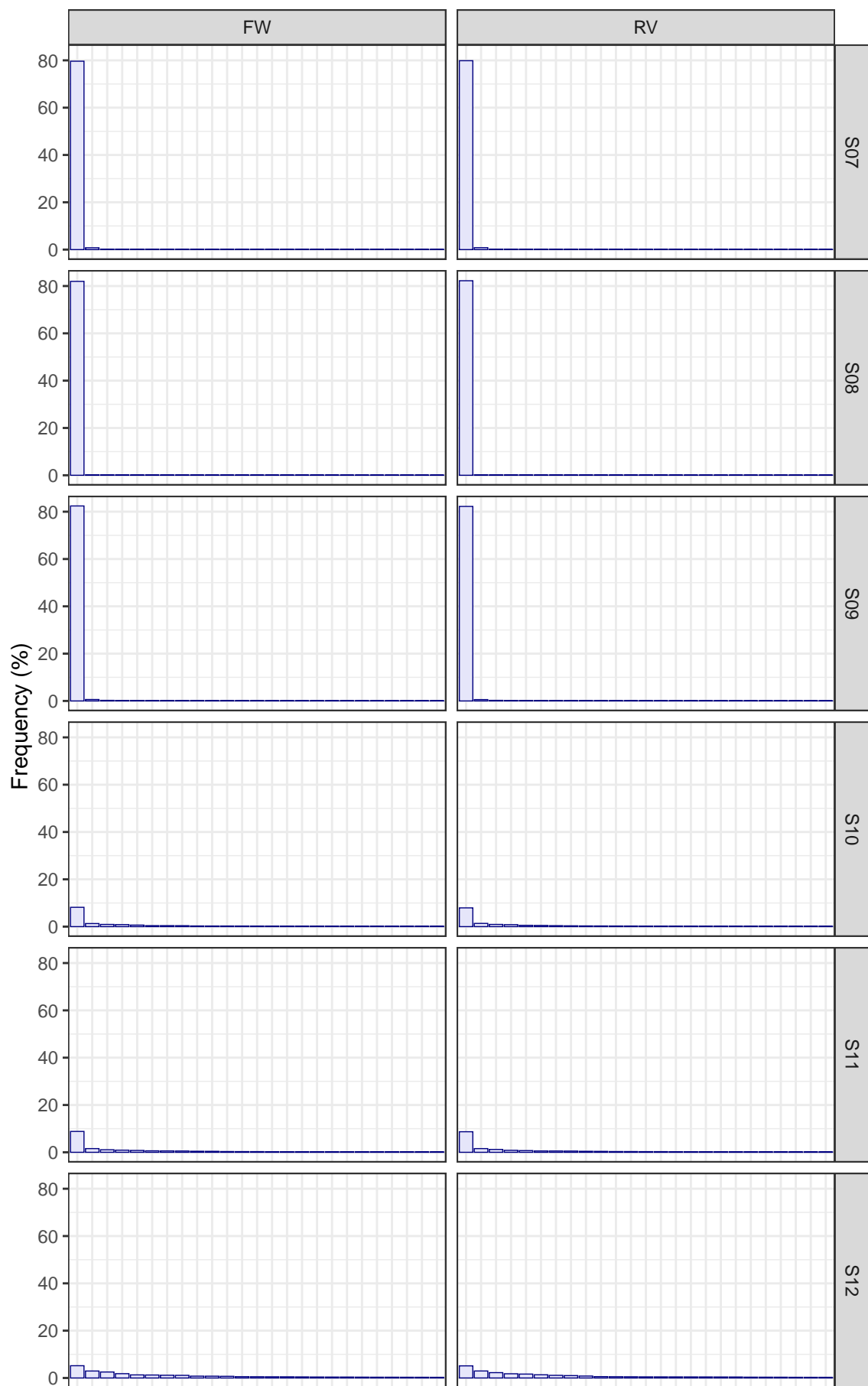

Supplement: Supplementary file 1 [file microorganisms-12-01011-s001.zip › Supplementary-File S1/SupplFig07_Top25_HaplotypeFreqs-2.pdf]

## Top 25 haplotypes

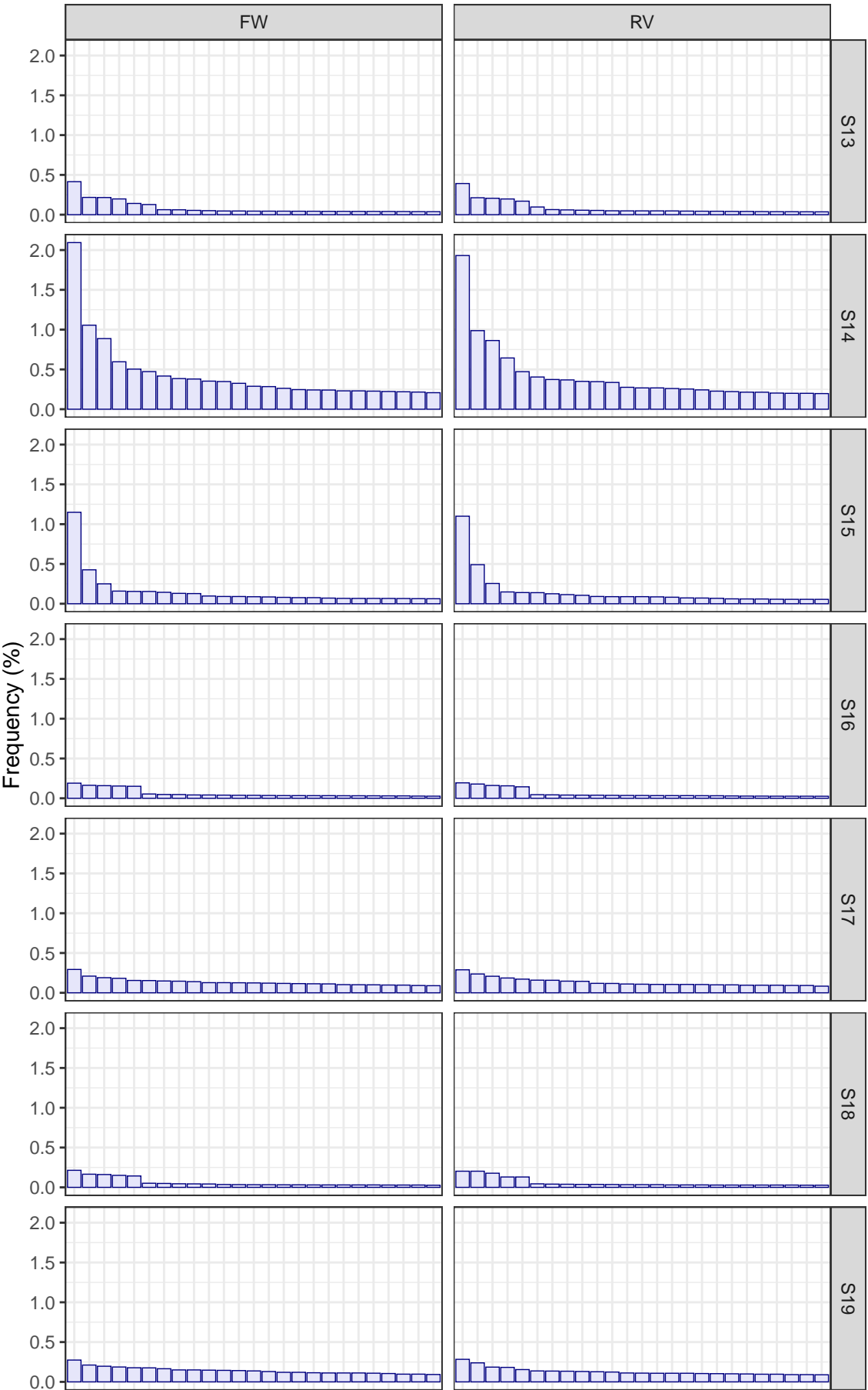

Supplement: Supplementary file 1 [file microorganisms-12-01011-s001.zip › Supplementary-File S1/SupplFig07_Top25_HaplotypeFreqs-3.pdf]

## Top 25 haplotypes

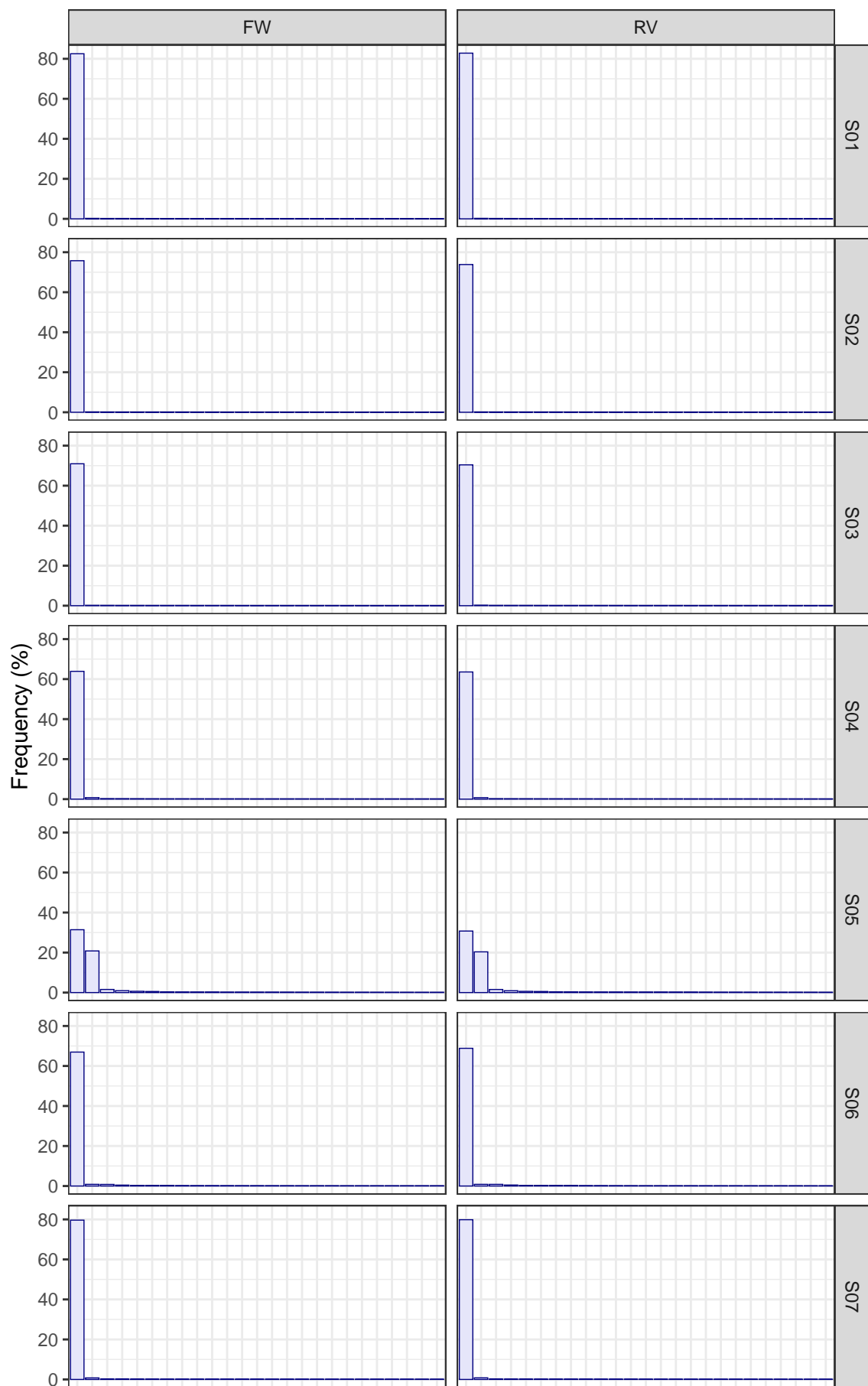

Top 25 haplotypes

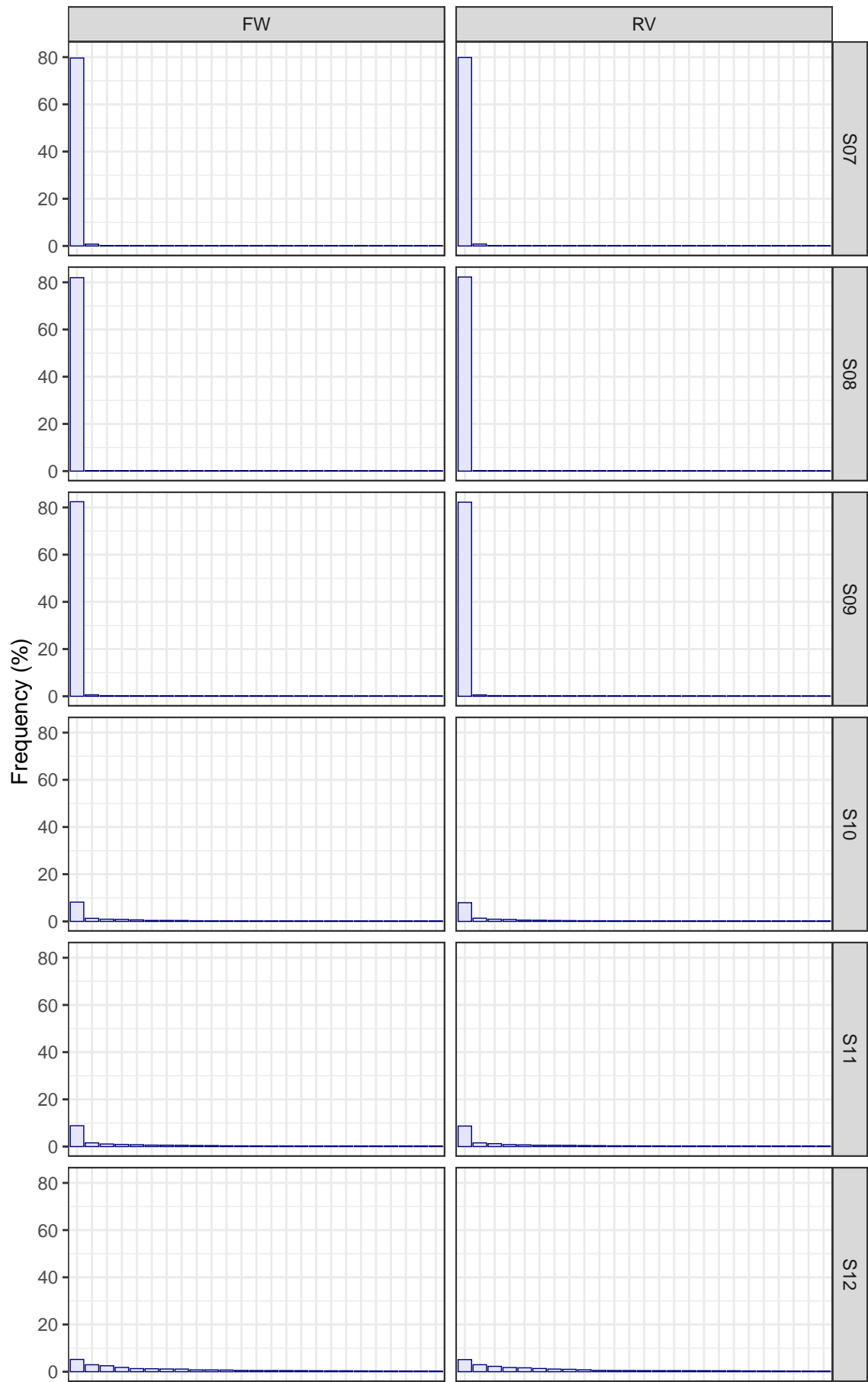

Top 25 haplotypes

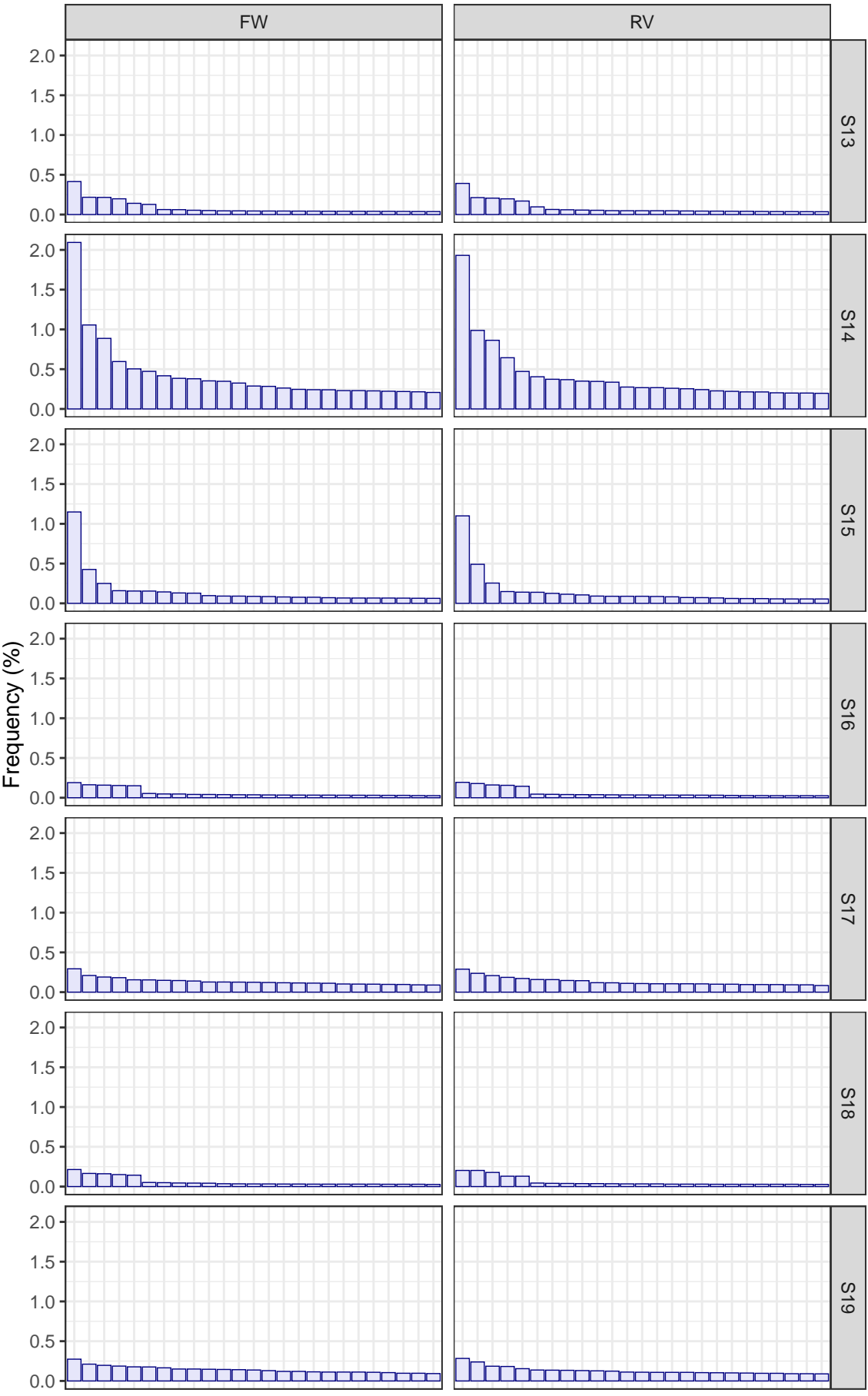

Supplement: Supplementary file 1 [file microorganisms-12-01011-s001.zip › Supplementary-File S1/SupplFig07_Top25_HaplotypeFreqs.pdf]

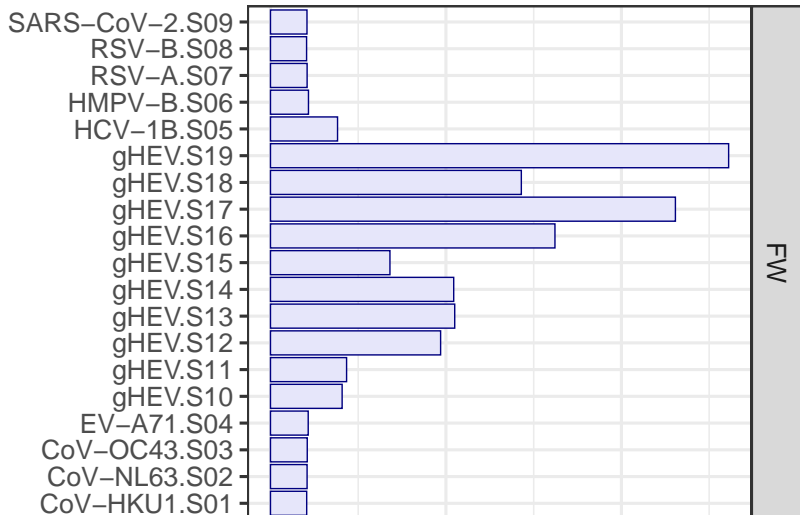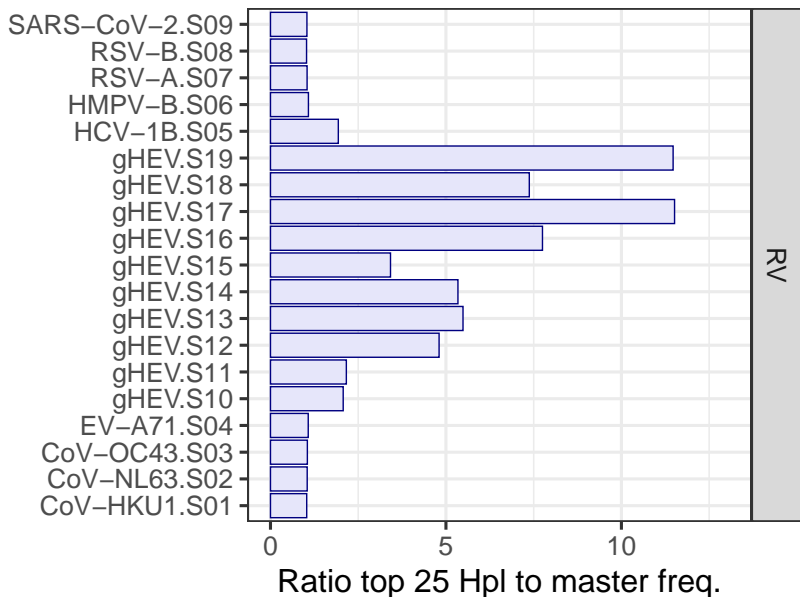

Supplement: Supplementary file 1 [file microorganisms-12-01011-s001.zip › Supplementary-File S1/SupplFig08_Top25HplRatio.pdf]

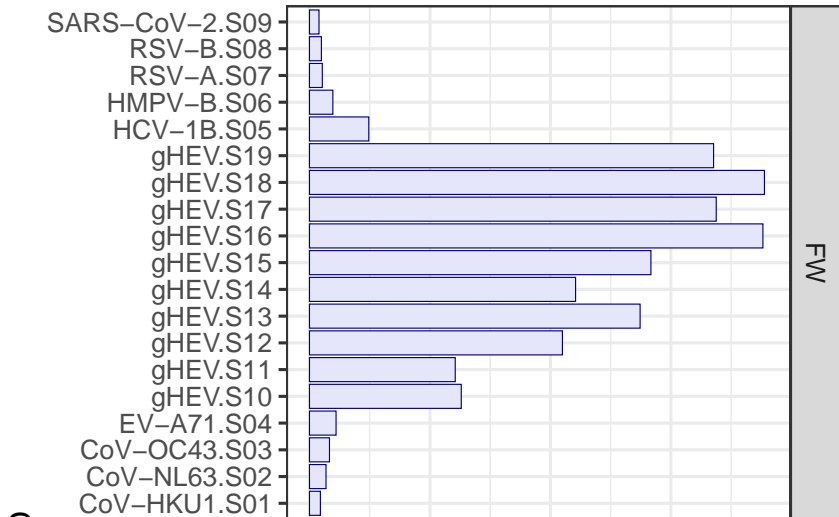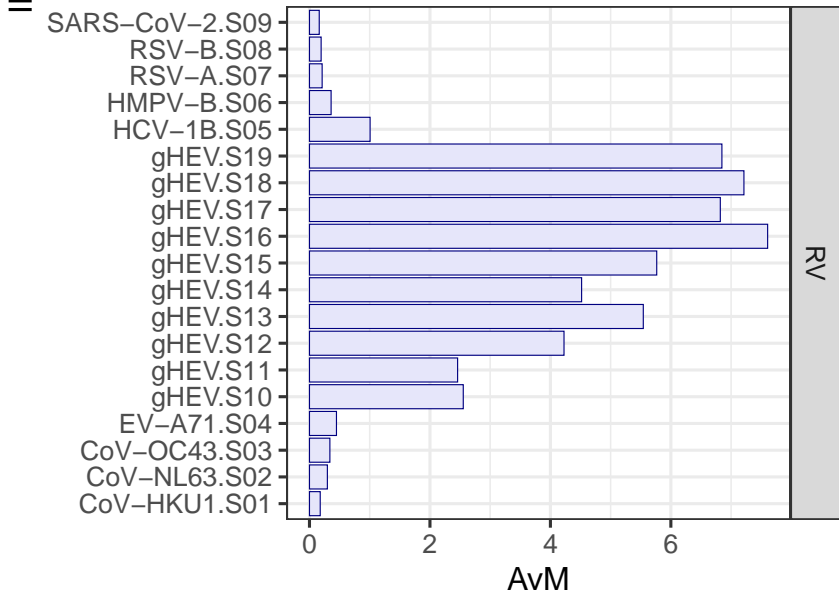

Supplement: Supplementary file 1 [file microorganisms-12-01011-s001.zip › Supplementary-File S1/SupplFig09_AverageSubstByRead.pdf]

# Subsampling Hill numbers

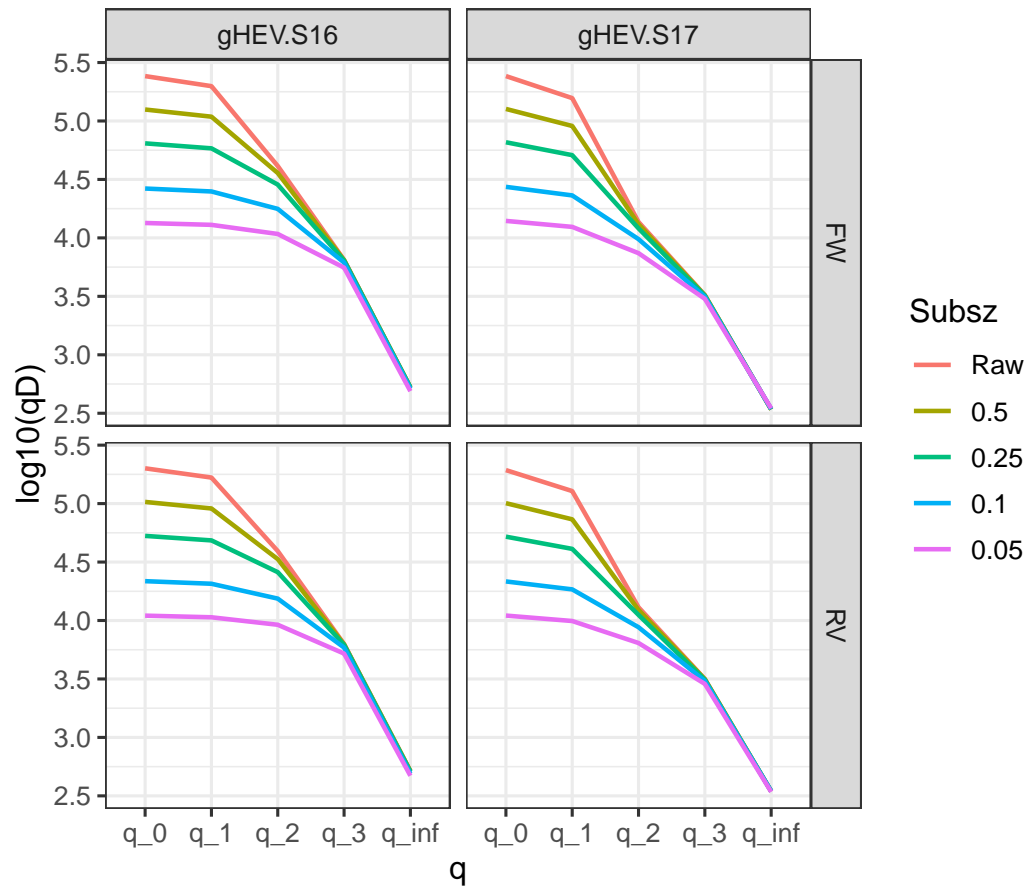

Supplement: Supplementary file 1 [file microorganisms-12-01011-s001.zip › Supplementary-File S1/SupplFig10_NewID.NoRplc_SubsamplingHillNumbers.pdf]

# Quasispecies fitness fractions (rarefied)

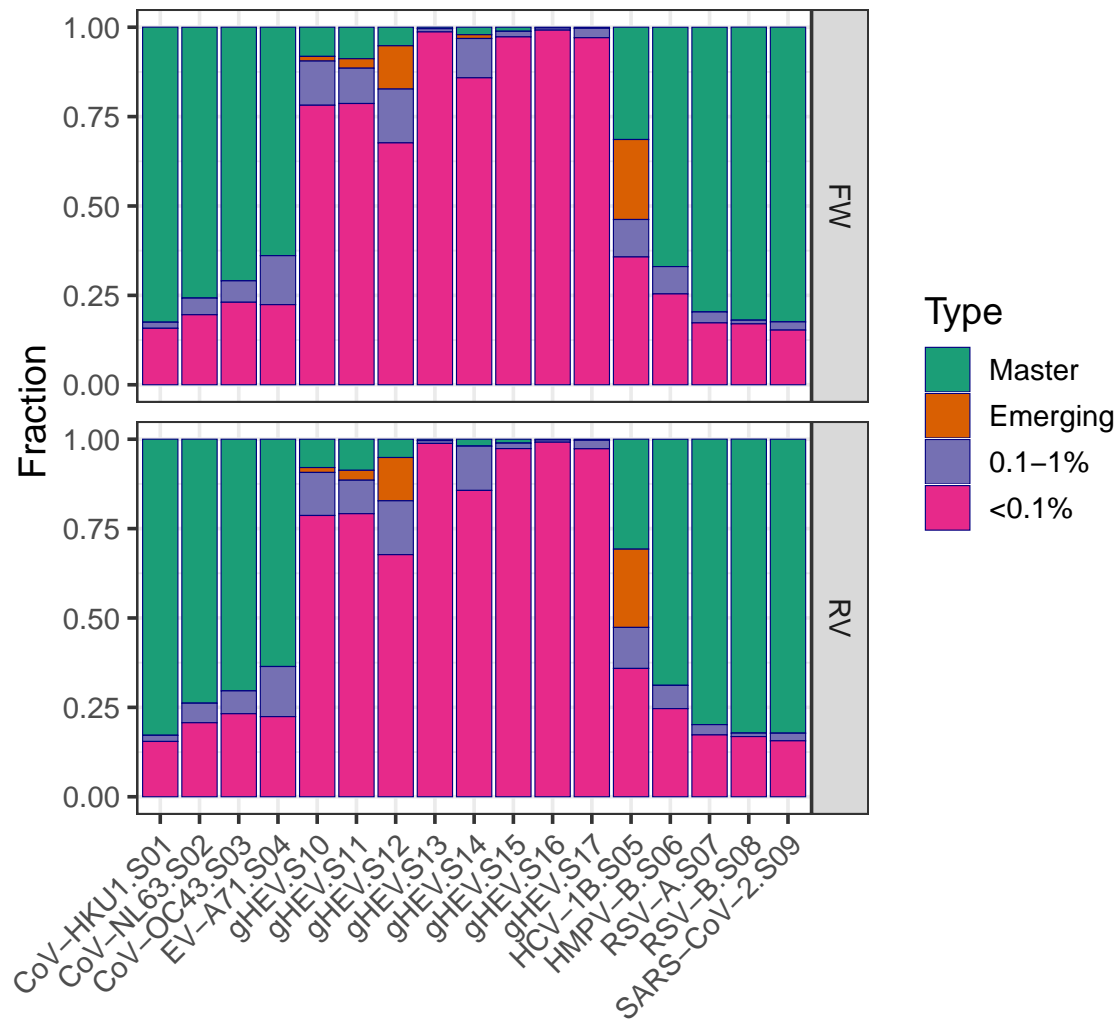

Supplement: Supplementary file 1 [file microorganisms-12-01011-s001.zip › Supplementary-File S1/SupplFig12_RarefiedQFF_FW&RV.pdf]

# Hill numbers profile

Rarefied to 194,000 reads

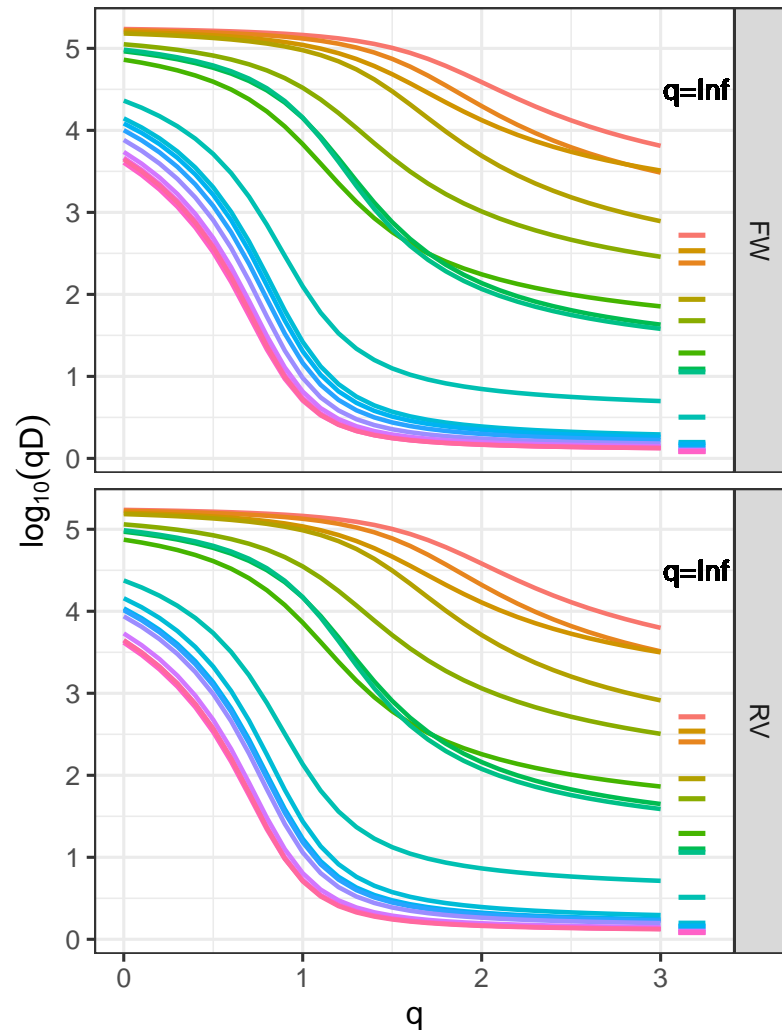

Supplement: Supplementary file 1 [file microorganisms-12-01011-s001.zip › Supplementary-File S1/SupplFig13_FW&RV_RarefiedHillNumbersProfile.pdf]

# Rarefaction impact on evenness

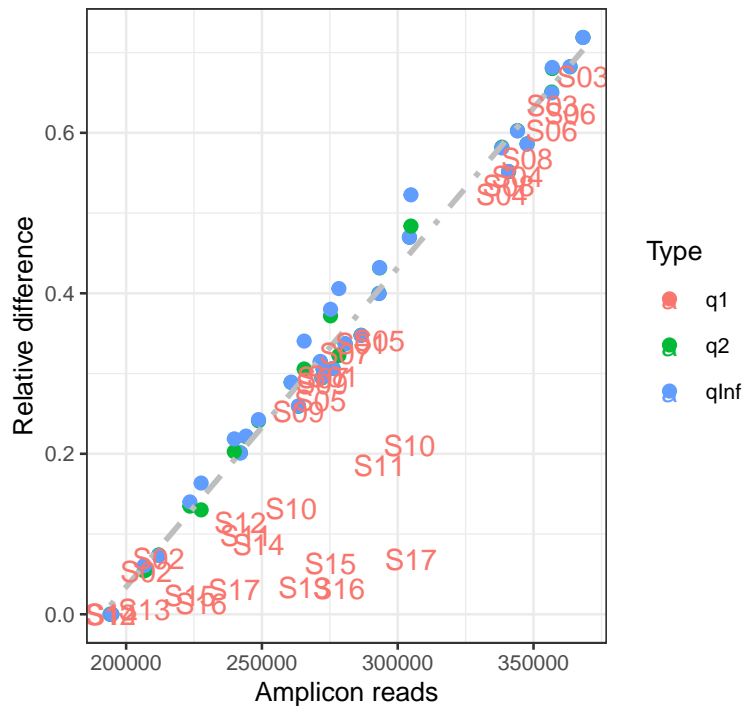

Supplement: Supplementary file 1 [file microorganisms-12-01011-s001.zip › Supplementary-File S1/SupplFig15_RarefactionImpactOnEvenness_ReadsVsRelDiff-2.pdf]

ID

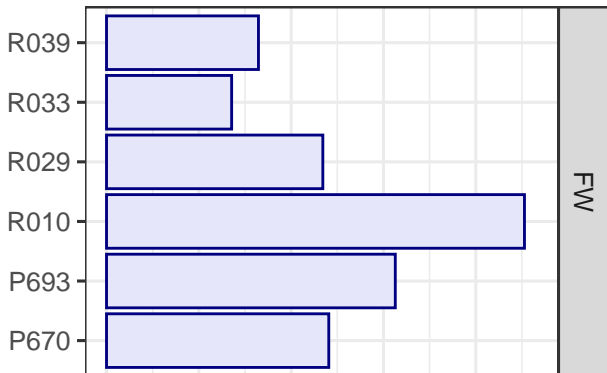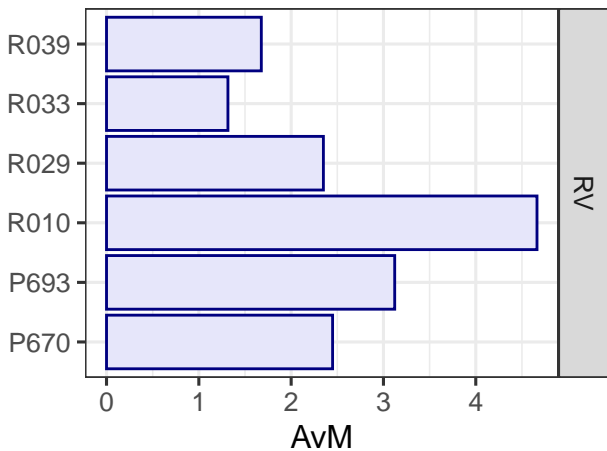

Supplement: Supplementary file 1 [file microorganisms-12-01011-s001.zip › Supplementary-File S2/AverageSubstByRead.pdf]

Fraction of reads for singletons (FW)

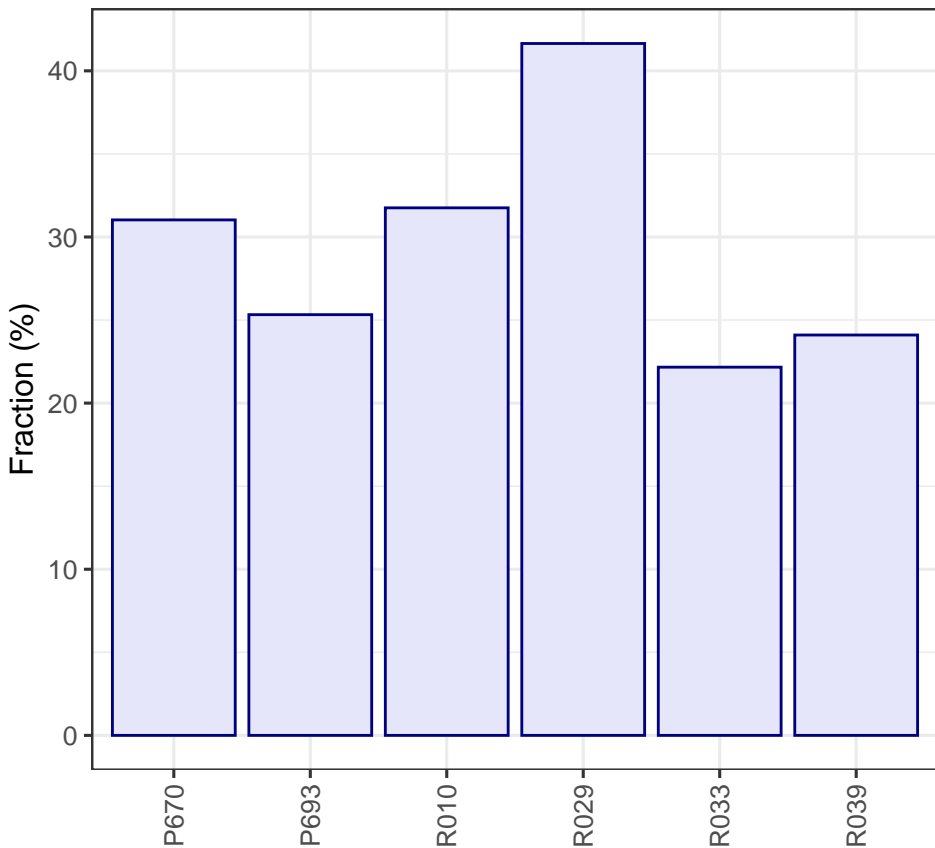

Supplement: Supplementary file 1 [file microorganisms-12-01011-s001.zip › Supplementary-File S2/DeepView_SingletonsFraction.pdf]

Full coverage by strand (reads)

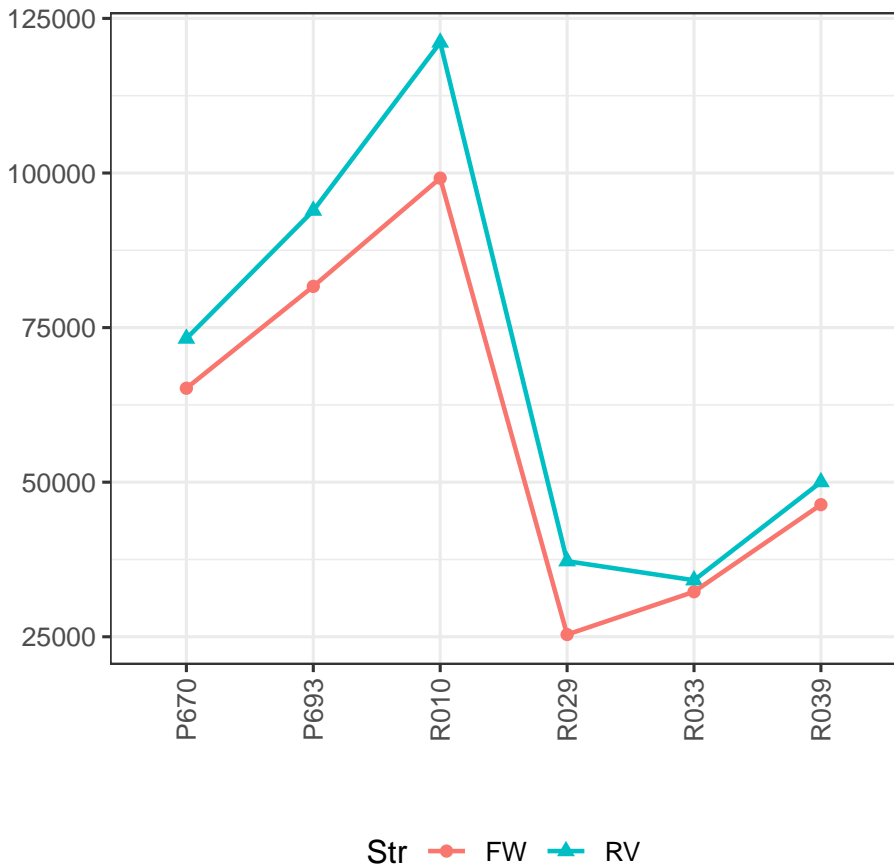

Supplement: Supplementary file 1 [file microorganisms-12-01011-s001.zip › Supplementary-File S2/FullCoverageByStrand.pdf]

Index of flatness at  $q=1$

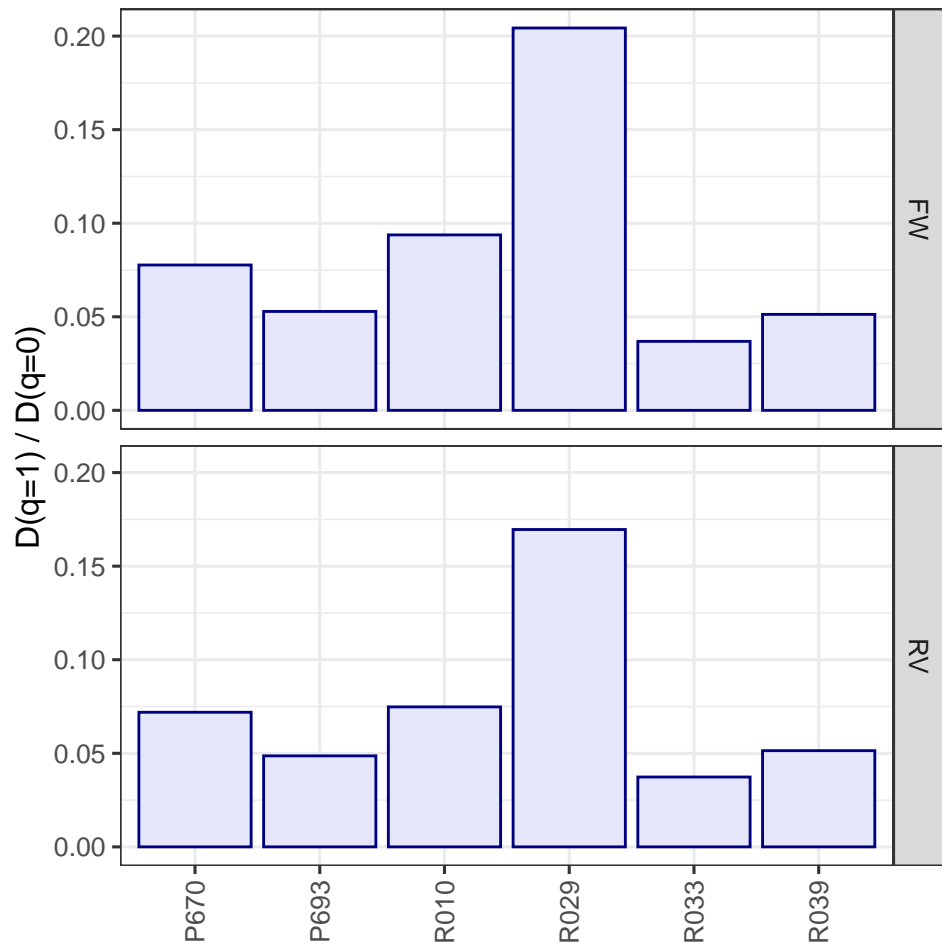

Supplement: Supplementary file 1 [file microorganisms-12-01011-s001.zip › Supplementary-File S2/FW&RV_FlatnessIndex-1.pdf]

Index of flatness at  $q=\text{Inf}$

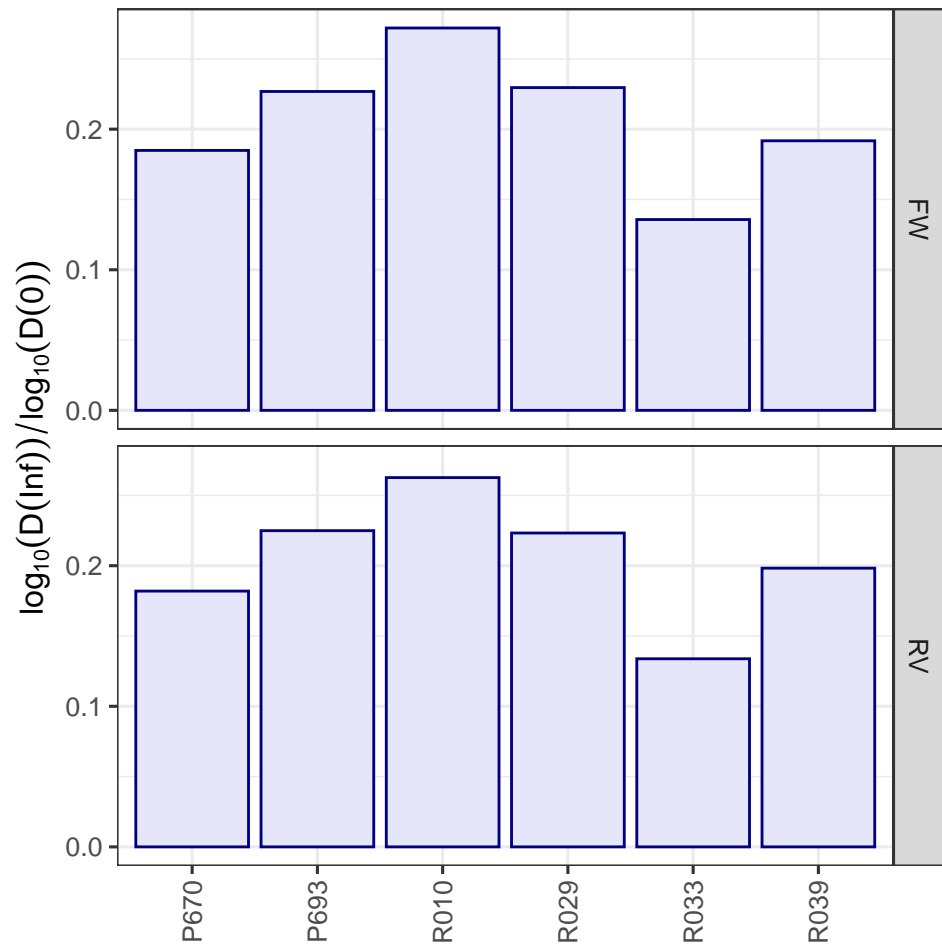

Supplement: Supplementary file 1 [file microorganisms-12-01011-s001.zip › Supplementary-File S2/FW&RV_FlatnessIndex-3.pdf]

Percentage of total reads

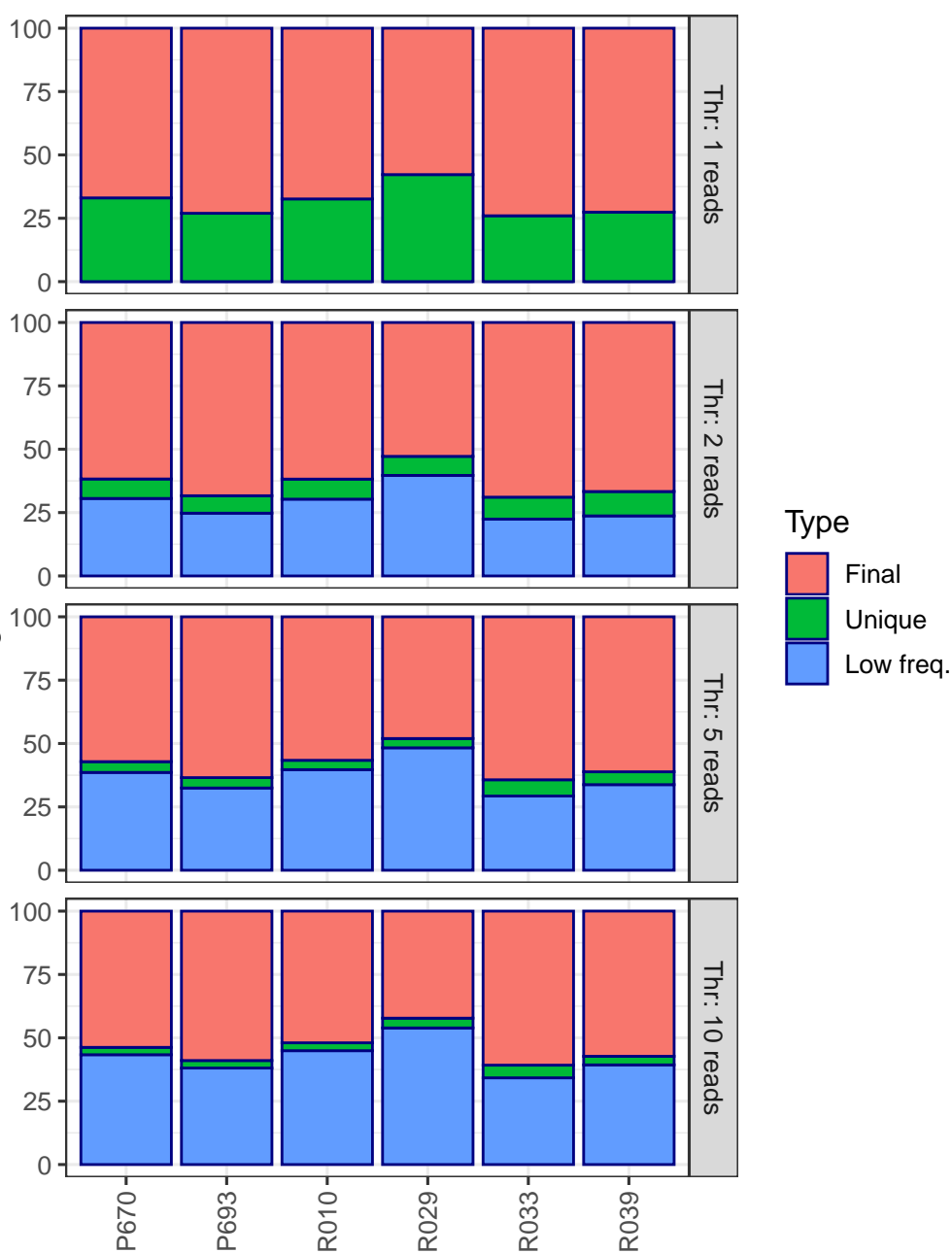

Supplement: Supplementary file 1 [file microorganisms-12-01011-s001.zip › Supplementary-File S2/Intersects_ReadsImpact_AtThresholds-1.pdf]

# Impact of freq. filter + strain intersection

Reads

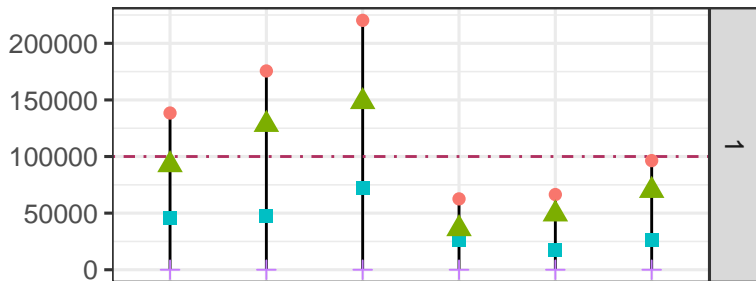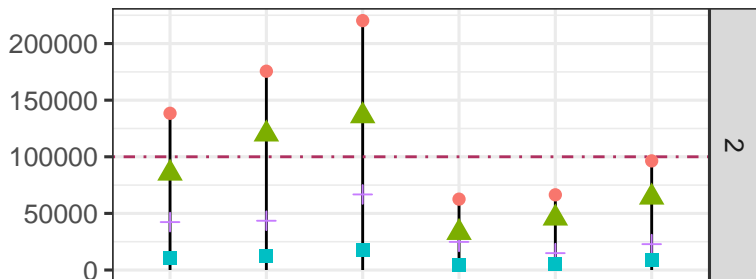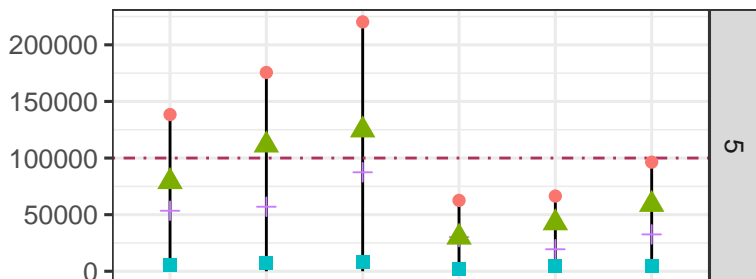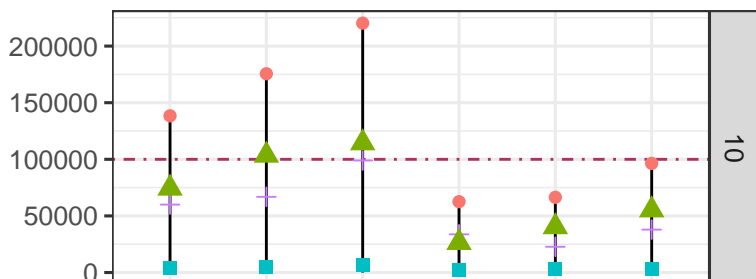

Type

- All
- Final
- Unique
- Low freq.

Supplement: Supplementary file 1 [file microorganisms-12-01011-s001.zip › Supplementary-File S2/Intersects_ReadsImpact_AtThresholds-2.pdf]

# Quasispecies fitness fractions

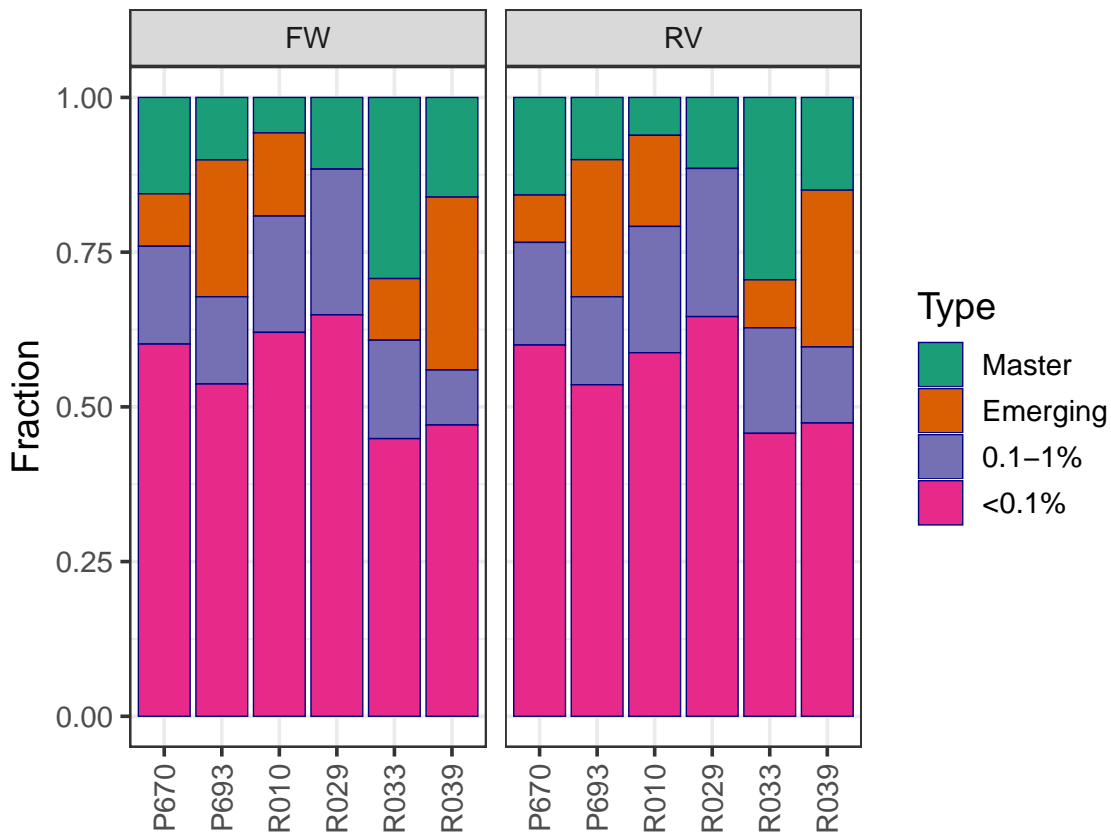

Supplement: Supplementary file 1 [file microorganisms-12-01011-s001.zip › Supplementary-File S2/QFF_FW&RV_hz.pdf]

# Hill numbers profile

Rarefied to 25,000 reads

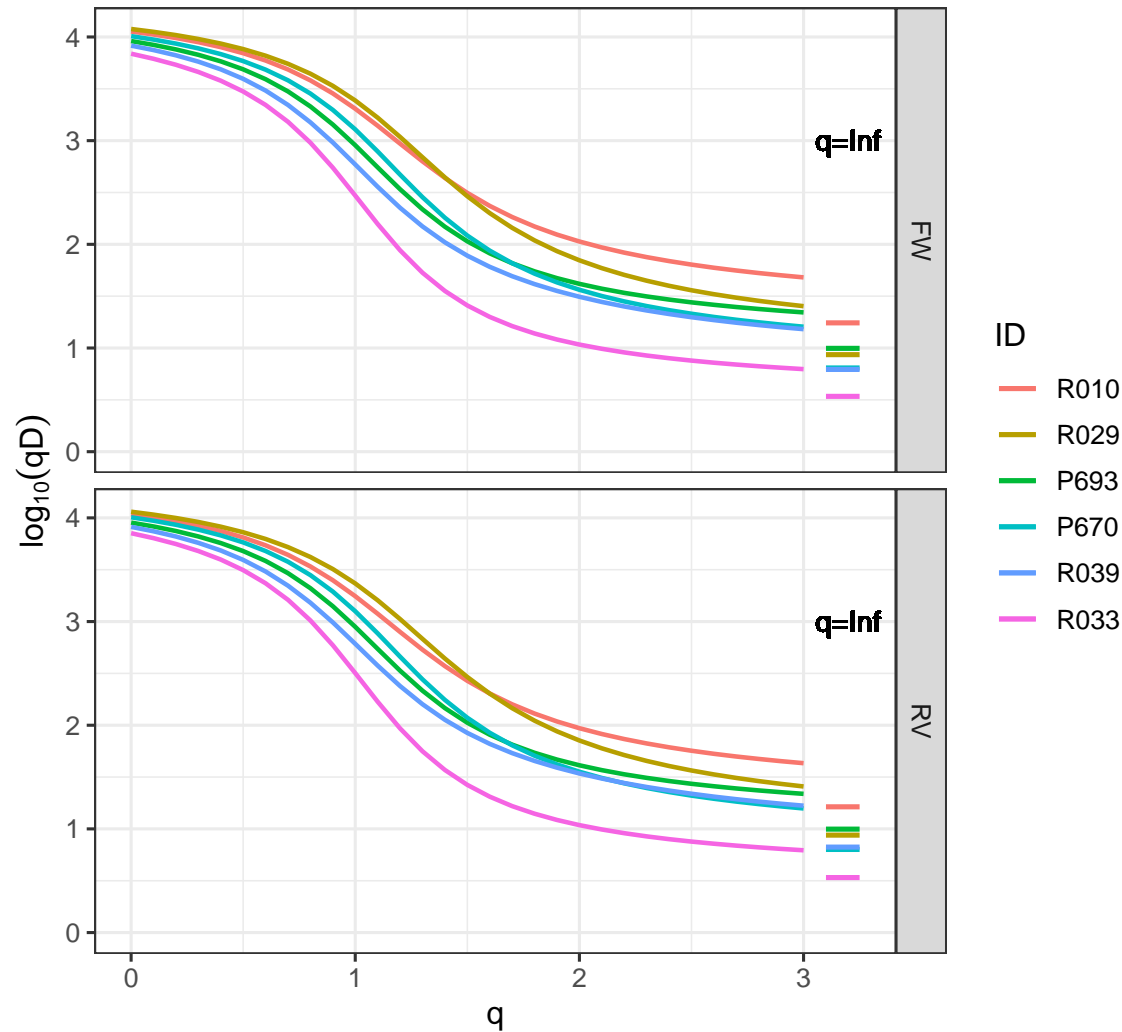

Supplement: Supplementary file 1 [file microorganisms-12-01011-s001.zip › Supplementary-File S2/Rarefied_HN_RE_RI_EvennessProfile-1.pdf]

# Relative logarithmic evenness

Rarefied to 25,000 reads

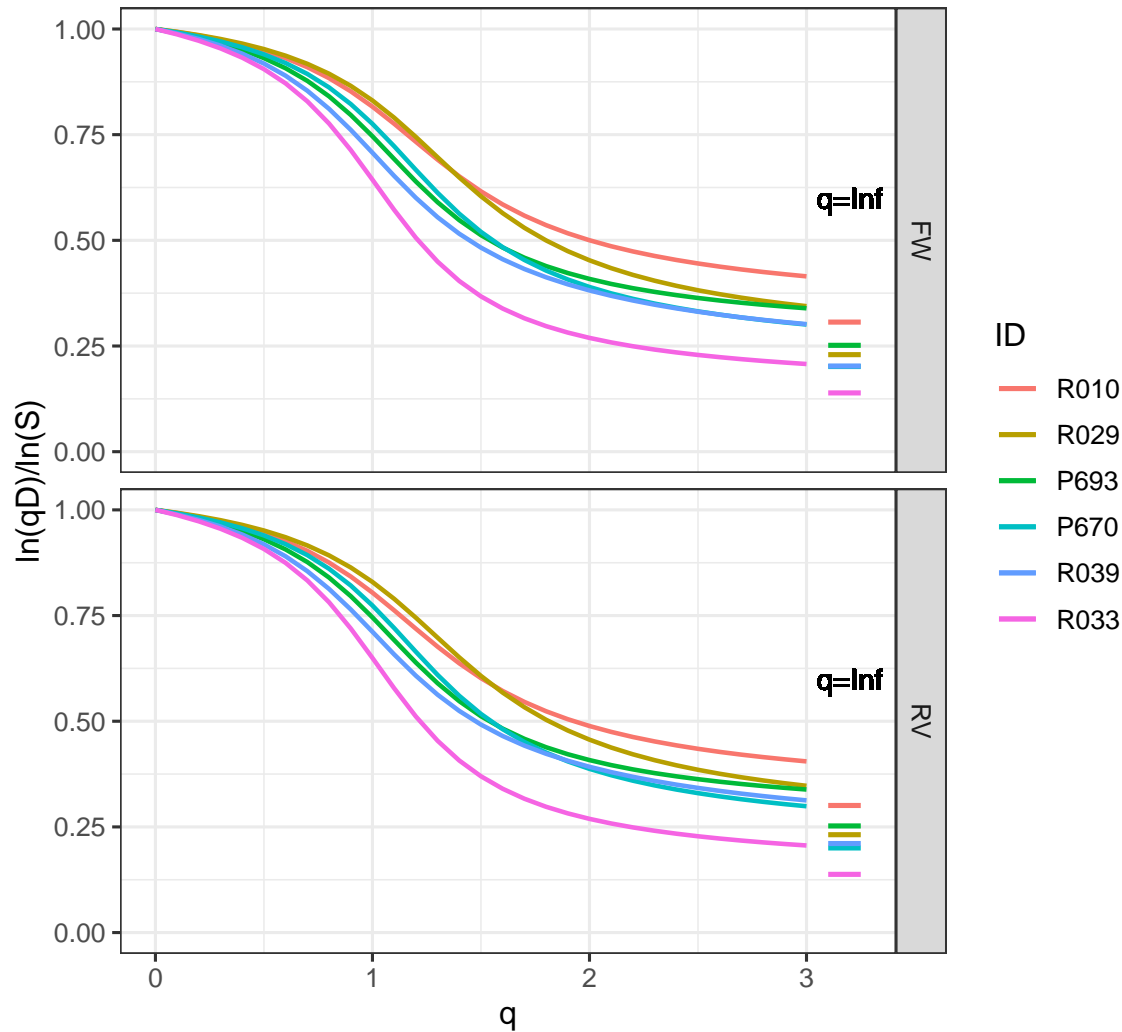

Supplement: Supplementary file 1 [file microorganisms-12-01011-s001.zip › Supplementary-File S2/Rarefied_HN_RE_RI_EvennessProfile-3.pdf]

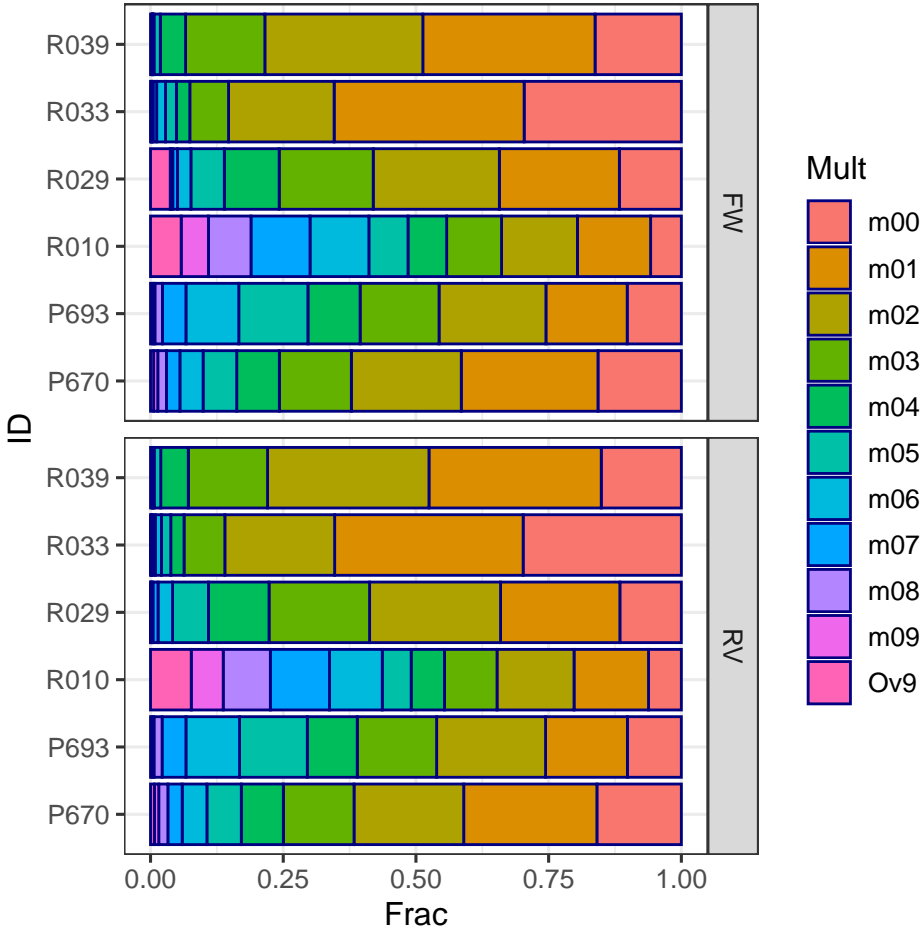

Supplement: Supplementary file 1 [file microorganisms-12-01011-s001.zip › Supplementary-File S2/ReadsByNSubst.pdf]

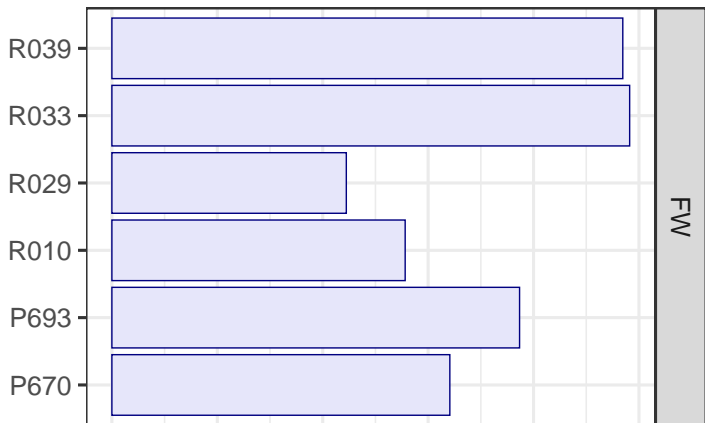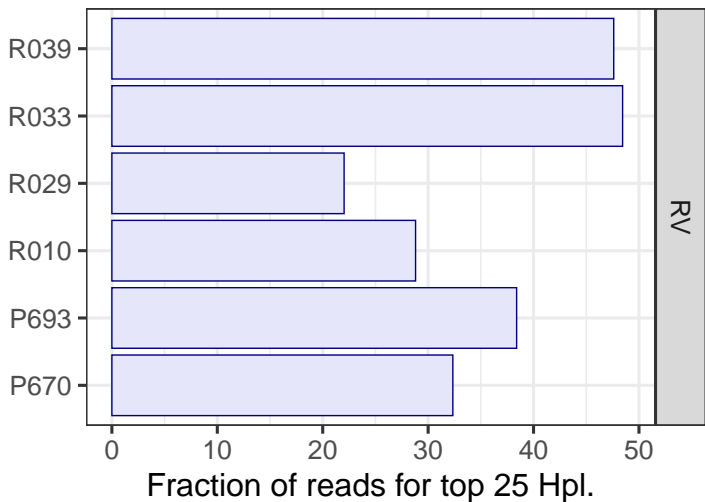

Supplement: Supplementary file 1 [file microorganisms-12-01011-s001.zip › Supplementary-File S2/Top25HplFraction.pdf]

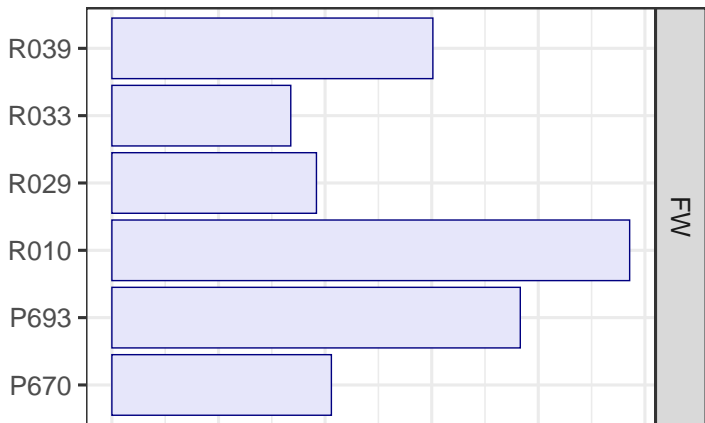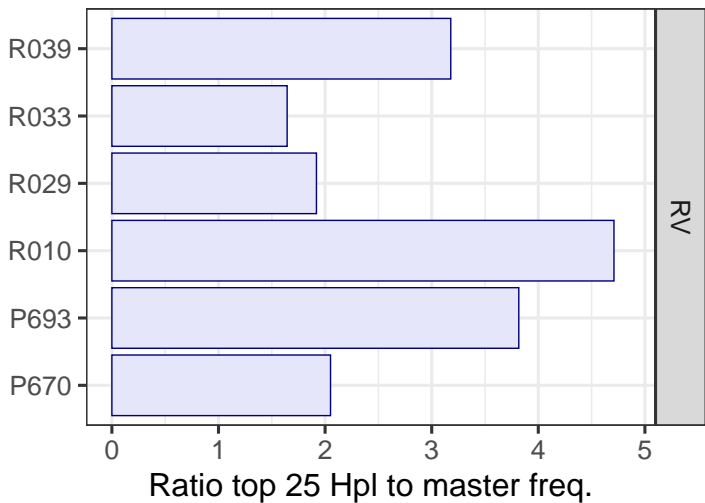

Supplement: Supplementary file 1 [file microorganisms-12-01011-s001.zip › Supplementary-File S2/Top25HplRatio.pdf]

# Top 25 haplotypes

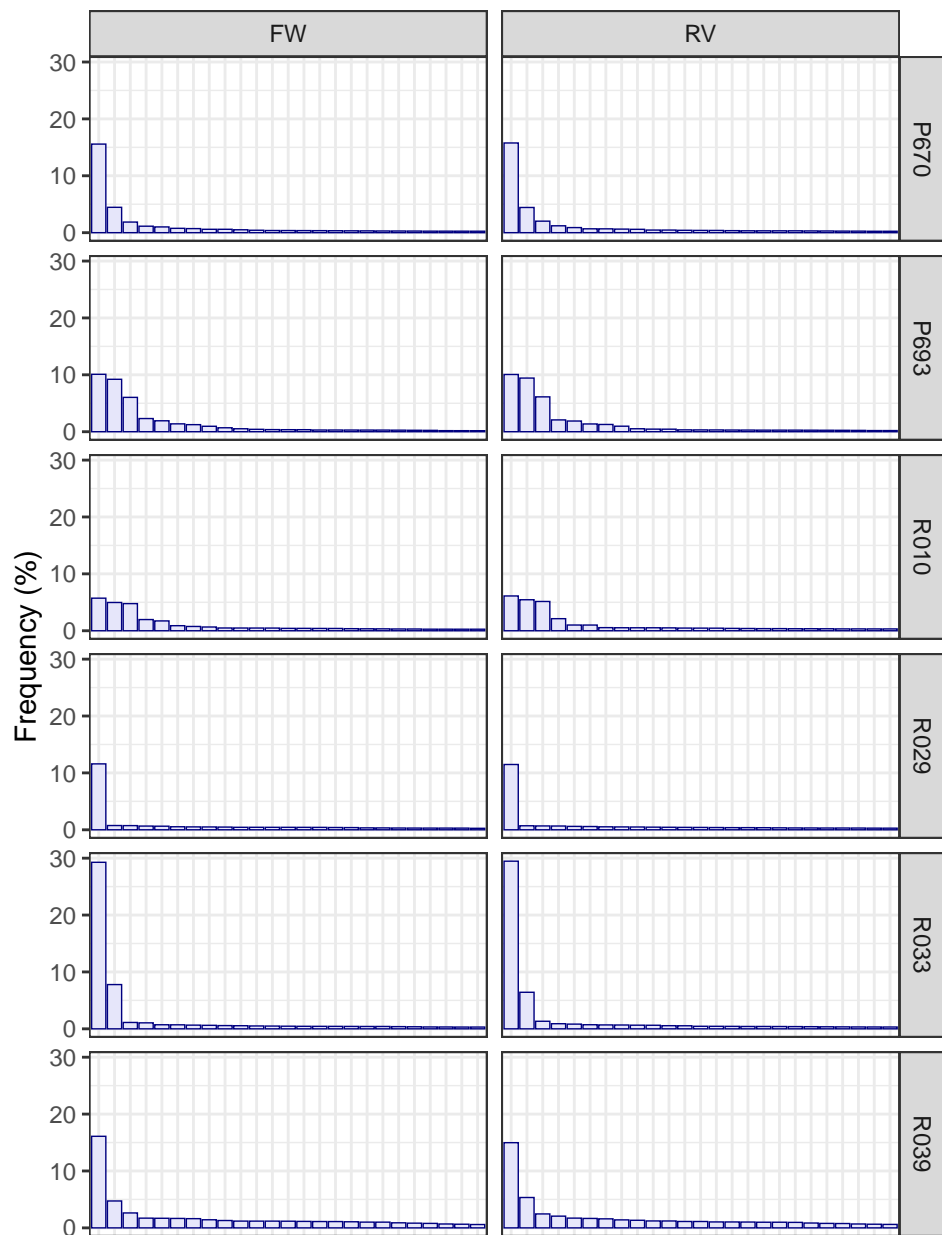

Supplement: Supplementary file 1 [file microorganisms-12-01011-s001.zip › Supplementary-File S2/Top25_HaplotypeFreqs.pdf]
